# Medusa’s gaze: Cell traces and fibrils but no collagen in permineralized Jurassic ichthyosaur bone

**DOI:** 10.1016/j.isci.2024.111523

**Published:** 2024-12-03

**Authors:** René-Paul Eustache, Alan Boyde, Xavier Jaurand, P. Martin Sander

**Affiliations:** 1 27 rue de la Forge, 27170 Combon, France; 2 Dental Physical Sciences Unit, Queen Mary University of London, Mile End Campus, London E1 4NS, UK; 3 Centre Technologique des Microstructures, Université Claude Bernard Lyon 1, 5 rue Raphael Dubois, Villeurbanne, 69622 Cedex, France; 4 Abteilung Paläontologie, Bonner Institut für Organismische Biologie, Universität Bonn, 53115 Bonn, Germany; 5 The Dinosaur Institute, Natural History Museum of Los Angeles County, Los Angeles, CA 90007, USA

**Keywords:** zoology, evolutionary biology, paleobiology, paleobiochemistry

## Abstract

Bone is formed by specialized cells whose activity allows bone to grow, change shape, and repair itself. Its composite structure of collagen fibrils and bioapatite nanocrystals gives bone exceptional mechanical strength. Using scanning electron microscopy, we show in fossil ichthyosaurs, 150 to 200 million years old, from the Jurassic of France and the UK, abundant and direct evidence of cellular activity on the fossilized forming, resting, and resorbing surfaces of bone trabeculae, as well as bone fibrils, Sharpey fibers, and cartilage fibers. These features are identical to those observed in fresh deproteinized mammalian bone, including human bone. Despite the striking similarity of the fibrils to those in modern bone, we found no evidence of collagen preservation. Fossilization removed non-mineralized components and exposed trabecular surfaces at the mineralization front. Cellular activity in skeletal tissue, familiar to any medical student, is preserved for >200 million years, and probably longer in vertebrate fossils.

## Highlights

• Jurassic ichthyosaur bone preserves internal surfaces at the nanostructural level
• SEM reveals forming, resting, and resorption surfaces as in modern deproteinized bone
• Ichthyosaur bone and cartilage biology were the same as in extant amniotes
• TEM and SEM show no evidence of collagen preserved in the fossil bone fibrils

## Graphical abstract

**Figure:**
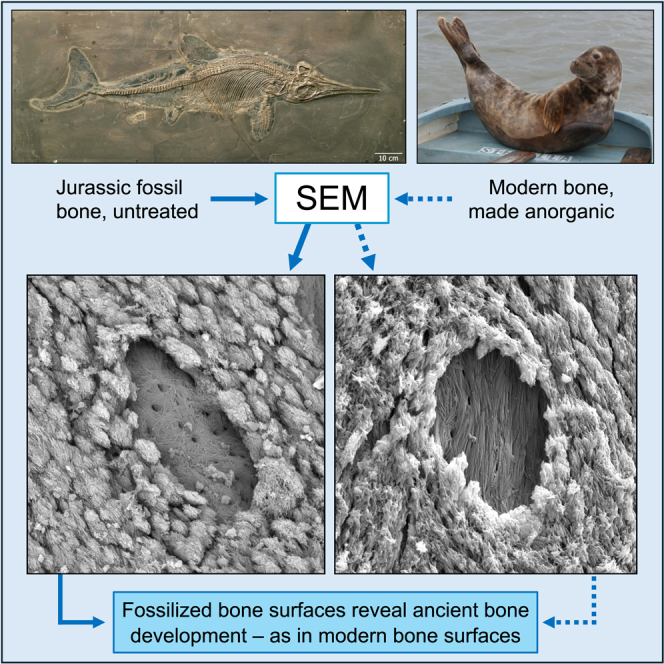

## Introduction

**Figure 1:**
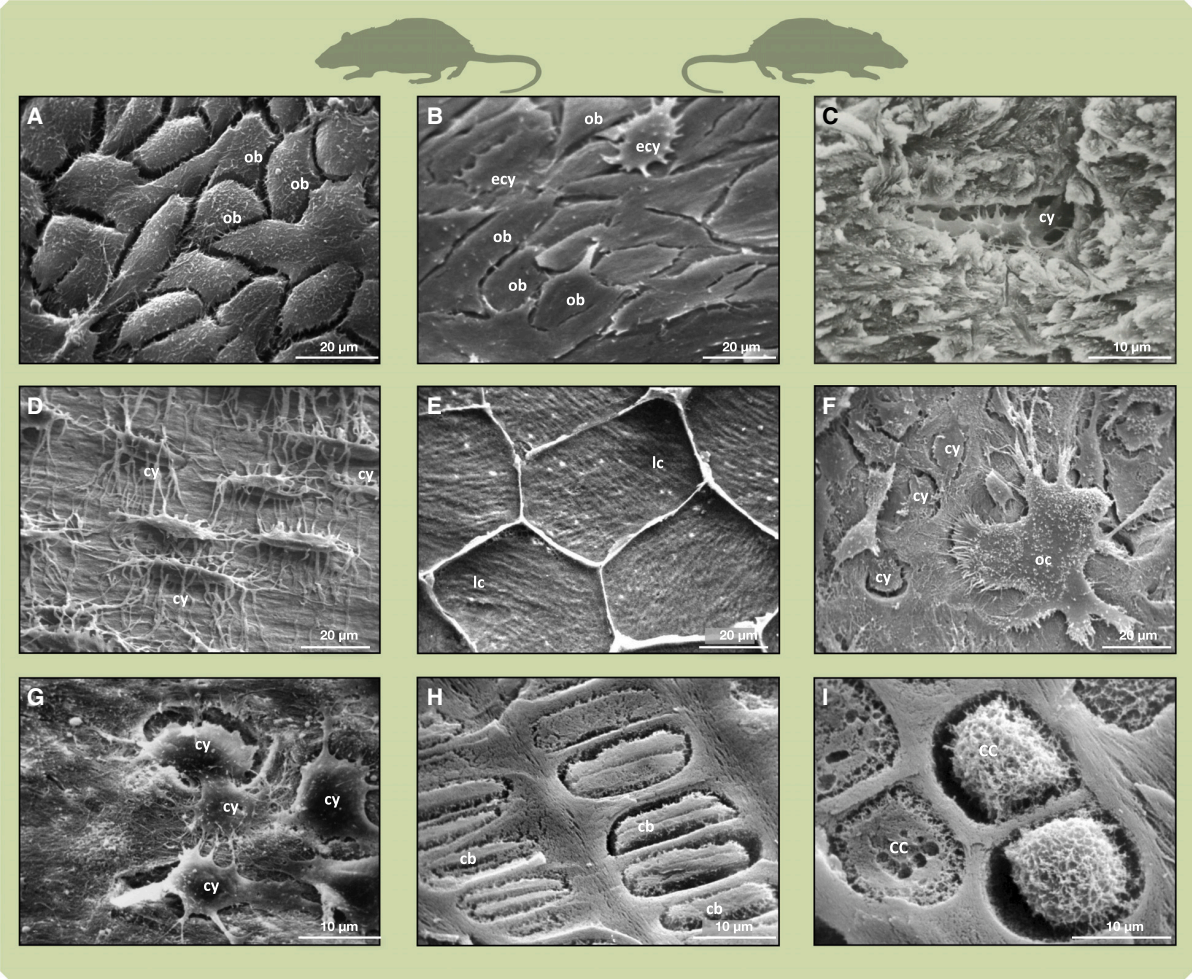
SEM micrographs of cell types involved in bone and cartilage formation and resorption Images (A) to (G) are arranged following the bone formation—bone resorption cycle. (A) Rat osteoblasts *in situ*. The spaces between cells are preparative shrinkage artifacts. (B) Rat osteoblast layer *in situ*, seen from the secretory surface. Two osteoblasts are seen in stages of differentiation into osteocytes. (C) Monkey osteocyte in lacuna exposed by fracturing. (D) PMMA (polymethyl methacrylate) cast of human osteocytes and canaliculi. (E) Human adipocytes as lining cells on bone resting surface. Note bone fibril arrangement visible through the bottom of cells. (F) Rat osteoclast *in situ*. The osteoclast is actively resorbing in several places, exposing osteocytes. (G) Rat osteocytes liberated by osteoclastic resorption migrating across the resorbed surface. (H) Rat chondroblasts in proliferating zone of growth plate. (I) Rat chondrocytes in hypertrophic zone of growth plate. Abbreviations: cb, chondroblast; cc, chondrocyte; cy, osteocyte; ecy, early-stage osteocyte; lc, lining cell; ob, osteoblast; oc, osteoclast. Scale bars in (A), (B), and (D–F) equal 20 μm; scale bars in (C) and (G–I) equal 10 μm. See Table 2 for sample preparation.

Bone is the main mineralized tissues of the vertebrate skeleton. It is a living tissue formed by specialized cells (Figure 1) whose activity allows bone to grow, change shape, and repair itself. Bone is composed of collagen fibrils and bioapatite nanocrystals, representing a composite structure that gives bone exceptional mechanical strength.^1^^,^^2^^,^^3^ Although bone older than a few million years appears fully mineralized by fossilization processes,^1^ replacing the collagen with apatite mineral, a growing number of reports suggest that collagen can also be preserved in fossil bones from deep time.^4^^,^^5^^,^^6^^,^^7^

In skeletal tissue biology of mammals, including humans, cellular activity and products are often studied by deproteinization of external and internal bone surfaces.^8^^,^^9^^,^^10^^,^^11^ Deproteinization removes the non-mineralized matrix components,^10^^,^^12^^,^^13^^,^^14^ revealing the traces and products that the cells leave behind on those surfaces to be studied by microscopic techniques such as scanning electron microscopy (SEM).

**Figure 2:**
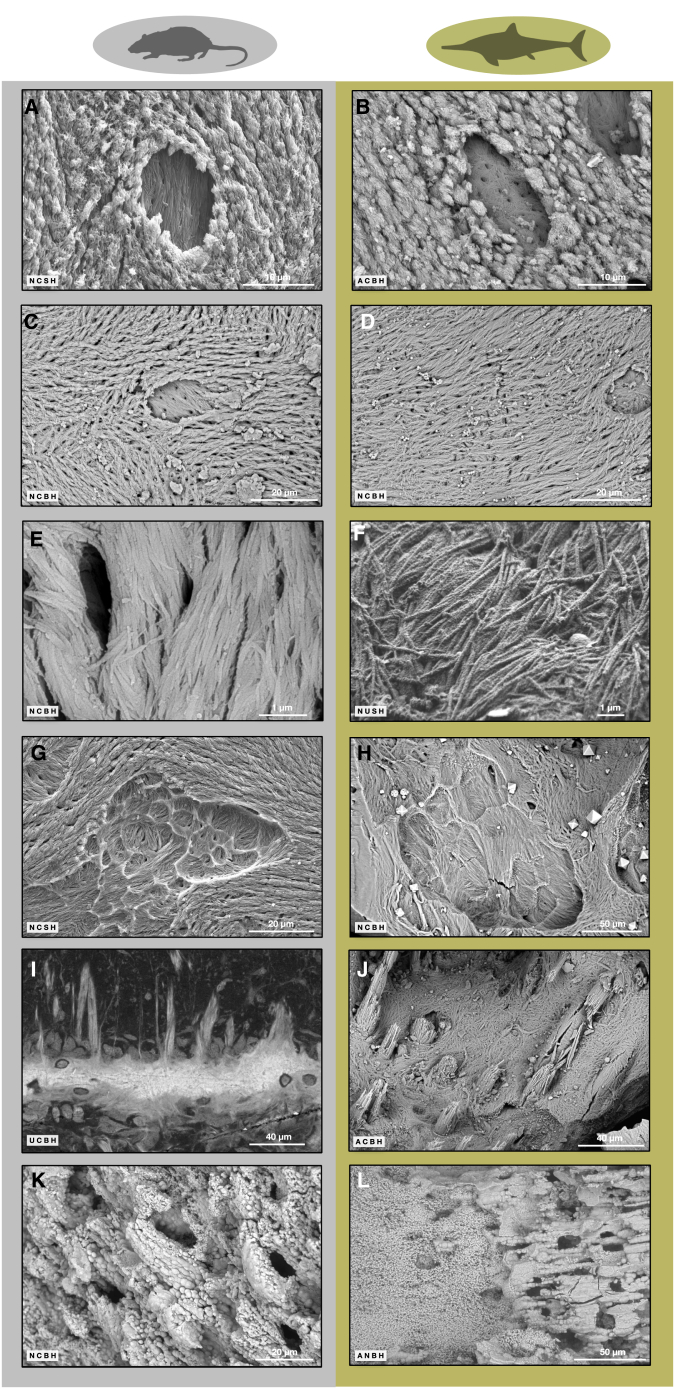
Comparison of modern mammal deproteinized bone and cartilage made anorganic and Jurassic ichthyosaur bone and cartilage internal surfaces Rat symbolizes all mammals. (A) Porpoise forming surface. (B) OV2 forming surface. (C) Porpoise resting surface. (D) OV1 resting surface. (E) Porpoise bone fibrils. (F) OV1 bone fibrils. (G) Porpoise resorbed surface. (H) OV1 resorbed surface. (I) Deer antler Sharpey fibers, PMMA embedded, cut and polished surface, iodine vapour stained. (J) OV1 Sharpey (extrinsic) fibers crossing open space. (K) Porpoise cartilage. (L) AB1 cartilage. Scale bars in (A) and (B) equal 10 μm; scale bars in (C), (D), (G), and (K) equal 20 μm; scale bars in (E) and (F) equal 1 μm; scale bars in (H) and (L) equal 50 μm; scale bars in (I) and (J) equal 40 μm.

Bone tissue in amniotes is formed in two basic ways, either by direct secretion by specialized cells (osteoblasts) on bone surfaces as periosteal bone or by deposition on and in cartilage as a precursor tissue (endochondral bone). Osteoblasts (Figures 1A and 1B) are secretory cells that produce mineralizable matrix, called osteoid, allowing the precipitation of hydroxyapatite. When osteoblasts secrete on an existing surface (scaffold), they produce collagen fibrils and bundles in patches of uniform orientation (domains) determined by osteoblast movement and orientation. The result is lamellar or parallel-fibered bone matrix mineralizing in forming surfaces (Figures 1A, 2A, and 2B). Where the matrix is secreted between cells without pre-existing scaffold, there is no movement, and the result is an irregular fibril arrangement—woven-fibered bone matrix.^9^,^11^^,^^15^^,^^16^^,^^17^^,^^18^^,^^19^^,^^20^^,^^21^ Here, a note on terminology is in order since the terms “fibril” and “fiber” are used inconsistently and sometimes interchangeably in the bone biological literature. We call the smallest structure observable in the SEM a “fibril”, the diameter of which is in the range of 100 to 200 nm. Such fibrils then may be arranged into fibril bundles.

Mineralization of the osteoid including the bone fibrils is also controlled by the osteoblasts through precipitation of hydroxyapatite nanocrystals both within and outside the fibrils.^22^^,^^23^ Fully mineralized bone consists of ca. 70% mineral by weight and ca. 30% collagen type I as well as of other organic constituents. Cartilage only mineralizes (called calcification) as a precursor to bone formation. Cartilage contains type II collagen fibrils. Mineralized regions, called calcospherites, in cartilage result from the initiation of an “infectious” mineralization process by matrix vesicles that originate from the surface of the chondrocytes.^24^

Some osteoblasts become incorporated into the mineralizing bone matrix as osteocytes (Figures 1C and 2A). Osteocytes stay in contact with each other through their canalicular processes (filopodia) (Figure 1D) and may live for decades. After matrix secretion ceases, resting surfaces (Figures 1E, 2C, and 2D) are covered by very thin lining cells that differentiate from osteoblasts and/or adipocytes (Figure 1E). Lining cells leave no traces on the mineralized bone surfaces.^1^^,^^25^

On resorption surfaces (Figures 1F, 2G, and 2H), osteoclasts (Figure 1F) secrete protons to dissolve the bone mineral, followed by enzymes to degrade the organic matrix, leaving characteristic clusters of resorption pits (Howship’s lacunae). Resorption liberates live, formerly entombed osteocytes, which form part of the cell population on the resorbed surface^17^ (Figures 1F and 1G). Other cells, probably macrophages, arrive to tidy up remnants of the surviving protein matrix.

Cellular differentiation processes are also central to endochondral bone formation where calcified cartilage matrix serves as the rapidly produced scaffold for bone (Figures 2K and 2L). Here, cartilage-forming cells hypertrophy (chondroblasts, Figures 1H and 1I) and may later dedifferentiate, mitose, and turn into osteoblasts.^26^^,^^27^^,^^28^

Critical to studying the surfaces of modern bone by SEM (Figures 2A, 2C, 2E, 2G, 2K, and S1) was the development of methods for removing cells from the matrix and unmineralized matrix from that already mineralized - subsumed under the terms “making bone anorganic” or “deproteinization.” The main methods are (1) inorganic oxidative treatment with bleach, i.e., sodium hypochlorite or hydrogen peroxide, (2) chemical and biological enzymatic treatment, i.e., maceration, (3) heat treatment by pyrolysis, and (4) low-temperature plasma ashing.^10^^,^^12^^,^^13^^,^^14^

We discovered that bone fossils from deep time may preserve all the same types of bone surfaces previously observed in modern bone made anorganic, as well as cartilage. Specifically, we investigated vertebrae from four different Jurassic ichthyosaurs (samples LR1, OV1, OV2, and AB1) that represent four different species and three different ontogenetic stages, from early juvenile to skeletally mature adult (STAR Methods and Table 1). Our ichthyosaur sample covers the whole range of periosteal and endochondral bone tissue types seen in amniotes (Figures S2–S33). For SEM observations of the fossils (STAR Methods), we accessed internal surfaces by sectioning because the outer bone surfaces were generally compromised by attack by organisms such as microbes, fungi, grazing invertebrates, etc., or by physical and chemical attack during fossilization^29^ (Figure S2).

**Table 1:** The fossil sample

Specimen	LR1	OV1	OV2	AB1
Taxon	cf. *Temnodontosaurus*	Ophthalmosauridae indet. sp. 1	Ophthalmosauridae indet. sp. 2	Ophthalmosauridae indet. sp. 3
Accession number	MPV 2024.1.1	MPV 2024.1.2	MPV 2024.1.3	MPV 2024.1.4
Locality	Lyme Regis, UK	Octeville, France	Octeville, France	Abingdon, UK
Geological age (Ma)	201–191	153	153	157–152
Ontogenetic stage	early juvenile	late juvenile	skeletally mature	late juvenile
Material	isolated centrum	partial skeleton	partial skeleton	isolated centrum
Position of sampled centrum	middle caudal	cervical	posterior dorsal	posterior dorsal
Length of sampled centrum (mm)	13	20	19	33
Transverse diameter of sampled centrum (mm)	35	56	40	83
Planes of section	parasagittal	sagittal, transverse	parasagittal	subtransverse

Four different Jurassic ichthyosaur specimens, derived from four different individuals from three different geological formations and localities, are listed here according to geological age. In case the exact geologic horizon of origin of the specimen is unknown, an age range is given based on the rocks exposed at the locality.

One of the French samples (OV1) shows open intertrabecular porosity, and trabecular surfaces could be directly investigated with the SEM without any further sample preparation (Figures 4A, 6A, S2, and S3). In the other fossils, the diagenetic calcite fill of the intertrabecular spaces formed during fossilization was dissolved with acetic acid (STAR Methods). To observe the continuity of the structures between the trabecular surfaces and the trabecular interior exposed by sectioning, some sections were first etched with 20% phosphoric acid while the intertrabecular spaces were still protected by the diagenetic calcite (STAR Methods and Figure S4).

## Results

### Forming, resting, and resorption surfaces in ichthyosaur trabecular surfaces

**Figure 3:**
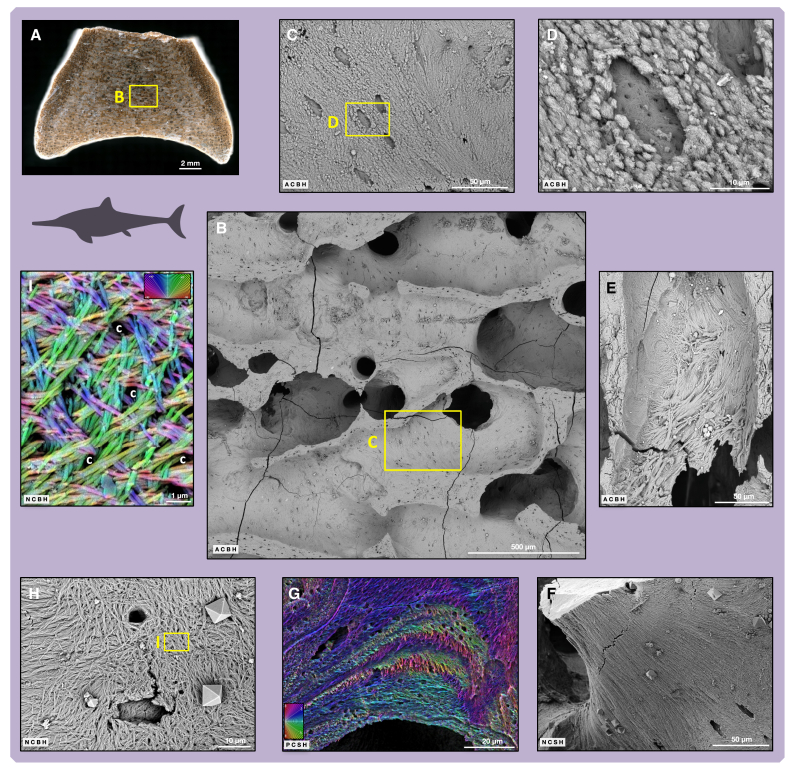
SEM investigations of ichthyosaur fossil internal bone surfaces: forming and resting surfaces For a Figure360 author presentation of Figure 3, see https://doi.org/10.1016/j.isci.2024.111523#mmc2. (A) OV2, overview image of partial sagittal section before diagenetic calcite dissolution with acetic acid. (B) OV2, overview of trabecular bone in the periosteal territory. (C) OV2, enlargement of (B), view into trabecular space with internal trabecular surface in periosteal territory. Note the open, half-formed osteocyte lacunae inside the bone forming surface representing the mineralizing front. (D) OV2, enlargement of (C), bone forming surface around an open osteocyte lacuna. (E) OV1, view into trabecular space with resting surface of large intrinsic fibril bundles deposited in a zone of resorption. (F) OV1, resting surfaces with parallel fibrils. Note open osteocyte lacunae and the octahedral pyrite crystals on the surface precipitated during fossilization. (G) OV2, phosphoric acid etched section of trabecula exposing layers of bone fibrils with different orientationvisualized in false color image. (H) OV1, resting surfaces of 2D fibrils with differing fibril directions. Note the octahedral pyrite crystals. (I) OV1, enlargement of (H), resting surface, fibril orientation is visualized by the false color image. Abbreviation: c, canaliculi. *Ichthyosaurus communis* silhouette by Scott Hartmann. Scale bar in (A) equals 2 mm; scale bar in (B) equals 500 μm; scale bars in (C), (E), and (F) equal 50 μm; scale bars in (D) and (H) equal 10 μm; scale bar in (G) equals 20 μm; scale bar in (I) equals 1 μm.

We observed forming surfaces consisting of the mineralized parts of collagen fibril bundles (often lozenge-shaped aggregates) (Figures 2B, 3C, 3D, and S5–S7). These are similar to those observed in forming surfaces at the mineralization front of modern lamellar and parallel-fibered bone (Figures 2A and S1A). There, the onset of osteoid mineralization is fibril/bundle-centered, in contrast to the mineralization of woven bone.^10^^,^^11^^,^^12^ In the ichthyosaurs, this resemblance indicates that we observe a fossilized bone surface whose morphology has been preserved at the level of the mineralization front (Figures 2B, 3C, and 3D) and not at the level of the osteoid surface as such. We therefore see the fossilized trace of a bone surface under formation.

Open osteocyte lacunae are embedded in the forming surfaces, and there are fully mineralized fibrils in the back walls of the lacunae (Figures 2B, 3D, and S7). As in modern bone, new forming surfaces in the fossils are not limited to previously resorbed areas but are also found overlying resting surfaces (Figure S7), indicating that osteoblast activity commenced on resting surfaces as well as on resorbed surfaces.^30^

In all samples, we observed large areas of continuous fibril orientation (domains) representing resting surfaces (Figures 2D, 3H, 3F, and S8–S15). The domains have a variable degree of anisotropy, depending on the specimen and location. Ordered domains with parallel fibrils (Figures 3F, S8, and S9A) and less ordered domains with non-parallel fibrils (Figures 3H, 3I, S11A, S12, and S13A) coexist in a juxtaposed and/or superimposed manner on surfaces of specimen OV1 (Figure S11B). After phosphoric acid etching of section surfaces, OV2 shows the abrupt change in fibril orientation characteristic of some lamellar bone deposits and the discontinuities in fibril orientation across cementing lines (Figures 3G and S9B). The fibrils may be grouped into parallel to 2D randomly oriented bundles as observed in modern bone. In OV1, some domains consist of a random entanglement of fibrils that are slightly undulating (Figure S11A). These domains were deposited on the surface of an underlying domain, forming layers, and are not to be confused with random fibrils in woven bone tissue deposited without a pre-existing scaffold. Large fibril bundles sometimes extend beyond the bone trabecular surface (Figures 3E, S13B, and S14) and can have quite large diameters, up to several micrometers. The large fibril bundles may represent the initiation of a scaffold for future trabecular connections. Large fibril bundles running from the subchondral marrow into the bone trabeculae have been reported, but rarely, in modern bone. However, they appear to be different from those observed here.^31^

Osteocyte lacunae are observed on forming surfaces, resting surfaces, and resorbed surfaces of the bone trabeculae but also on fracture surfaces (Figures 3C, 3D, 4D–4F, and S15–S17A). The lack of diagenetic mineral infill in OV1 extends to open osteocyte lacunae and even canaliculi (Figure S16), but open lacunae are also seen in the other samples on trabecular surfaces. In LR1, osteocyte lacunae inside the bone tissue retain some internal fill that becomes visible on etching of the internal surfaces (Figures 4G, S17B, S18, and S19). This internal fill, which either represents natural casts of osteocyte lacunae or of osteocytes themselves, consists of diagenetic mineral precipitates as indicated by its crystalline nature and energy dispersive X-ray (EDX) analysis (Figures S19 and S34–S55; Tables S1–S4), specifically apatite.

**Figure 4:**
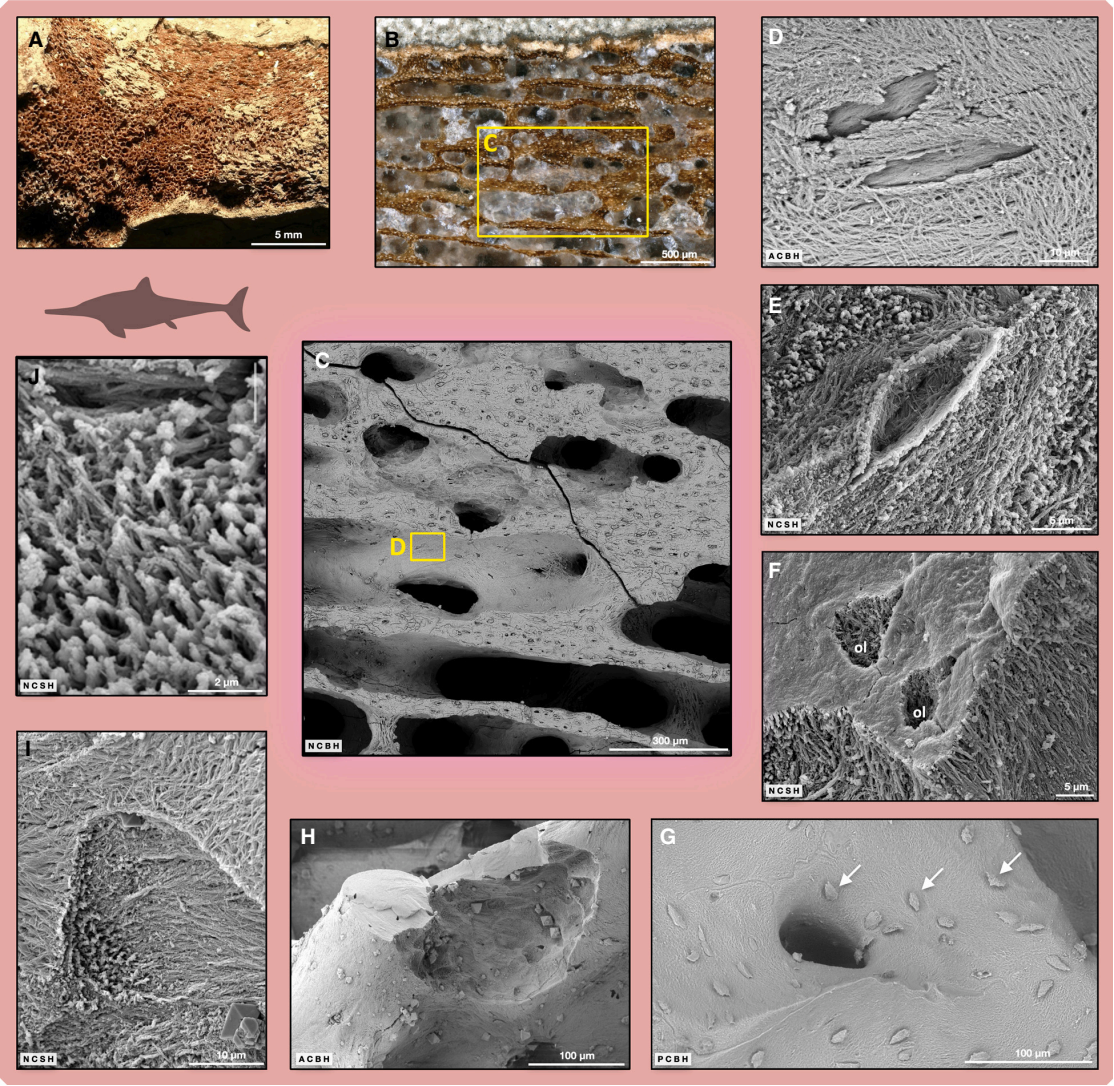
SEM investigations of ichthyosaur fossil internal bone surfaces: osteocyte lacunae and resorption surfaces (A) Photograph of fossil vertebra OV1 with open porosity. (B) OV1, incident light microscope image of section before calcite dissolution with acetic acid. (C) OV1, SEM overview of periosteal territory. (D) OV1, enlargement of (C), resting surface with open osteocyte lacunae. (E) OV1, osteocyte lacunae exposed by osteoclastic resorption during the animal’s lifetime. The osteocyte may have been released by this process. (F) OV1, fracture surface exposing open osteocyte lacunae with bone fibrils inside. (G) LR1, mineralized infill of osteocyte lacunae (arrows) revealed by etching. (H) OV2, resorbing surface (Howship’s lacunae) on trabecular surface exposing different fibril orientations. (I) OV1, higher magnification image similar to (H). (J) Close-up of bone fibrils truncated by osteoclast activity. Abbreviation: ol, osteocyte lacuna. Scale bar in (A) equals 5 mm; scale bar in (B) equals 500 μm; scale bar in (C) equals 300 μm; scale bars in (D) and (I) equal 10 μm; scale bars in (E) and (F) equal 5 μm; scale bars in (G) and (H) equal 100 μm; scale bar in (J) equals 2 μm.

Different shapes of osteocyte lacunae relate to the organization of the surrounding collagen matrix. If the matrix is made of parallel fibrils, as in slow-growing primary bone, osteocytes are elongated in parallel with the surrounding fibrils. If the matrix is more disorganized or “woven,” the lacunae have a stellate morphology and are much more numerous (seen in etched section in LR1) (Figure S18A). Lacunae embedded in trabecular surfaces have a flat floor (Figures 3D, 4D, and S15), similar to osteocytes undergoing entrapment observed with SEM in modern bone.^11^^,^^32^^,^^33^

Howship’s lacunae covering resorption surfaces are well imaged in all samples (Figures 2H, 4H, 4I, and S20–S22). In the sectioned surfaces, prior resorption surfaces are visible as reversal lines (Figure S4A). The characteristic shape of the Howship’s lacunae with scalloped borders records prior osteoclastic activity, identical to that observed in fresh bone (Figures 2G and 2H). Howship’s lacunae cannot be confused with any postmortem boring activity by microorganisms such as tunneling (Figure S2). Each of these small adjacent cavities excavated by the osteoclasts exposes superimposed fibril domains with contrasting orientations (Figure S20) where the fibrils were cut by osteoclast activity (Figures 4I and 4J). In the samples from France (OV1 and OV2), osteocyte lacunae opened up by resorption (Figures 4E, S17A, and S21) are empty. As is typical for osteoclast activity, it neither discriminates between preexisting structures nor different mineralized tissue types, i.e., bone vs. cartilage (Figures S22 and S24B). Resting resorbed surfaces^17^ were not observed in this study.

### Differences between ichthyosaur samples reflect differences in bone cell activity

A qualitative assessment of the different cellular activity states of bone surfaces associated with bone remodeling at a given time can be made from the amount, morphology, and distribution of forming, resting, and resorption surfaces (see supplemental information). In the four ichthyosaur specimens examined, resting surfaces occupy the greatest area of the bone trabeculae. Forming and resorption surfaces are well represented in OV2, AB1, and LR1. Surprisingly, in the late juvenile OV1, the forming surfaces are present only in trace amounts, in contrast to the numerous resting and resorption surfaces that are present. The ichthyosaur samples also differ in the arrangement of the fibrils on the bone surfaces. OV2 has a majority of ordered fibril domains, whereas OV1 has a greater number of domains with less ordered fibril arrangement in 2D. LR1 and AB1 show fibril arrangements intermediate between OV1 and OV2. The large bundles of fibrils that sometimes detach from the surface (Figures 3E, S13B, and S14) are frequent in OV1 and are present in traces in the other three specimens.

### Ichthyosaur cartilage and its transformation to endochondral bone

**Figure 5:**
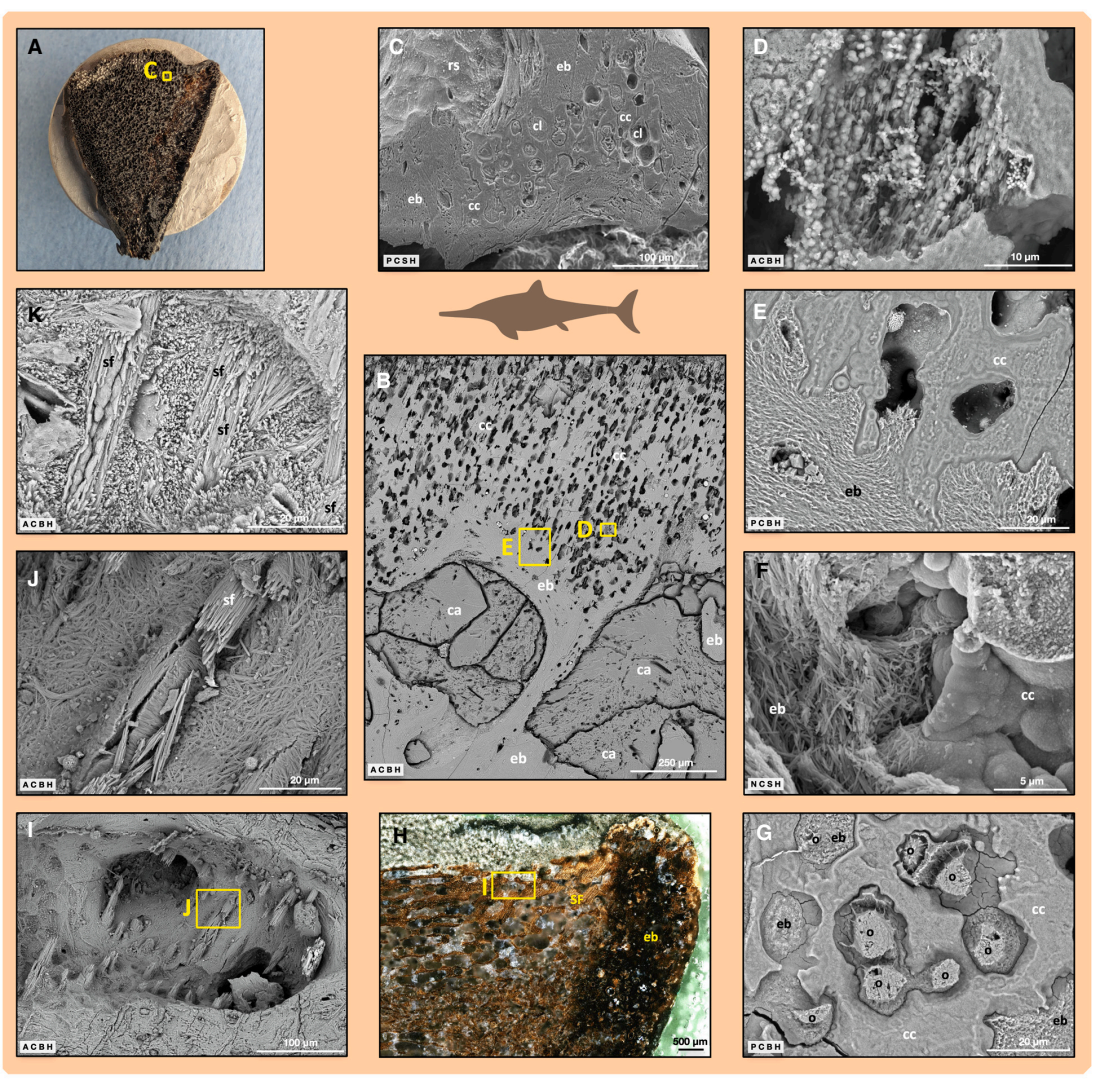
SEM investigations of ichthyosaur fossil internal bone surfaces and sections: cartilage, endochondral bone, and extrinsic fibers (A) OV1, photograph of transverse section on SEM stub following calcite dissolution. (B) LR1, columns of large chondrocyte lacunae in calcified cartilage (top) being replaced by endochondral bone. (C) OV1, overview of cartilage on rib articular surface. (D) LR1, enlargement of (B), mineralized cartilage fibers with calcospherites. (E) LR1, enlargement of (B), phosphoric acid etched surface revealing bone fibrils (lower left) and mineralized cartilage with Liesegang rings (upper right). (F) OV1, subchondral bone (left) next to mineralized cartilage. (G) LR1, chondrocyte lacunae with osteocyte casts and bone matrix inside. The osteocyte casts represent osteocytes that differentiated from chondrocytes. (H) OV1, incident light microscope image of parasagittal section showing location of periosteal bone with Sharpey fibers of the intervertebral joint capsule insertion. (I) OV1, Sharpey fibers protruding into trabecular space. (J) Close-up of (I) showing the bone fibrils enwrapping the Sharpey fibers. (K) AB1, high-magnification view of Sharpey fibers exposed by osteoclast activity showing the partially mineralized fiber interiors. This is evidence that Sharpey fibers biomineralize from the outside in. Abbreviations: ca, calcite pore fill; cc, calcified cartilage matrix; cl, chondrocyte lacuna; eb, endochondral bone; o, osteocyte lacunae; rs, resorption surface; sf, Sharpey fibers. Scale bar in (A) equals 10 mm; scale bar in (B) equals 250 μm; scale bars in (C) and (I) equal 100 μm; scale bar in (D) equals 10 μm; scale bars in (E), (G), (J), and (K) equal 20 μm; scale bar in (F) equals 5 μm; scale bar in (H) equals 500 μm.

In principle, fossil cartilage (Figures 2L, 5B–5G, and S24) is more difficult to interpret than fossil bone tissue because unmineralized cartilage may also fossilize as well as the already (i.e., *in vivo*) mineralized (calcified) cartilage.^34^^,^^35^ The cartilage layer on the anterior and posterior articular surface of the centra is well represented in OV1 and AB1, but particularly in LR1 because of its early ontogenetic stage (Figure 5B). Cartilage is also present at the rib articular facets of centra in the transverse sections (Figure S24).

In the ichthyosaur fossils, we observed a broad sequence of endochondral ossification, starting with parallel columns of large chondrocyte lacunae that are perpendicular or oblique to the articular surface (isogenic groups of chondrocytes) (Figure 5B). The chondrocyte columns are separated by a homogeneous matrix which, by phosphoric acid etching as well as by fossil osteoclast activity, can be shown to contain mineralized collagen fiber traces (Figures S22B and S33). Further evidence of mineralization of the cartilage matrix consists of numerous calcospherites (roughly spherical mineralized regions within cartilage matrix). Mineralized cartilage collagen fibers also extend into open spaces associated with the calcospheritic mineralization (Figures 5D and S23B). Calcospherites are aligned in parallel to the columns of chondrocyte lacunae and also to the mineralized collagen fibers and are usually seen in the walls of the lacunae. The initiation of bone formation (osteoid deposition and subsequent mineralization of fibers) is observed on internal cartilage surfaces (Figures 5E, 5F, and S23A). Calcospherites can be seen in OV1 and LR1 in the residual cartilage within the isolated calcified cartilage islands remaining within the bone trabeculae. On etched sections, these calcospherites show concentric Liesegang banding (rhythmic changes in crystallite size and composition; Figure S25A).

Unmineralized chondrocytes in the fossils are, as in modern bone, very much larger than the osteocytes and are preserved as spherical to ovoid lacunae (Figures 5B and S24). In calcified cartilage islands, the chondrocyte lacunae are frequently connected by gaps or fenestrae. We also observed chondrocyte lacunae filled with bone and, in some cases, one or more osteocyte lacunae or lacunar casts surrounded by bone fibrils within the chondrocyte lacuna (Figures 5G, S26, and S27). This observation supports the conclusions of Shapiro and Boyde, Roach, and Ricqlès^26^^,^^27^^,^^36^ that some chondrocytes dedifferentiate and re-differentiate into osteoblasts.

### Ichthyosaur extrinsic (Sharpey) fibers and patterns of their mineralization

We observed extrinsic Sharpey fibers in all fossil samples except for OV2, in which internal remodeling and postmortem destruction of the superficial bone apparently had obliterated them. In sagittal sections of OV1 and AB1, Sharpey fibers occur in primary trabecular bone on the ventral side of the centrum near the anterior and posterior borders of the periosteal territory (Figures 5H–5J). The fibers generally parallel the territory border, representing the insertion of the joint capsule surrounding the intervertebral disk.^34^ Sharpey fibers were also observed in a transverse section of AB1 revealing the insertion of the rib joint capsule (Figure 5K).

The Sharpey fibers of the intervertebral joint capsule are often preserved in a peculiar way as “stumps,” protruding from the trabeculae into the intertrabecular pore (bone marrow) space and having a matching projection on the other side of the space (Figures 5I and S28). The “stumps” imply that when the trabeculae and the marrow spaces in between them were formed, they incorporated the pre-existing, i.e., extrinsic, Sharpey fibers. In modern bone, this phenomenon was described by Boyde and Jones.^8^^,^^12^

Light microscopy reveals Sharpey fibers inside the primary periosteal bone at the same locations. Osteoclast activity as well as etching reveals that the periphery of the Sharpey fibers is well mineralized but not the fiber cores (Figures 5K and S29). This is in agreement with what has been observed in modern bones, resulting from Sharpey fiber mineralization from the outside in.^8^

### Structure of fossil bone fibrils and cartilage fibers does not reveal collagen

**Figure 6:**
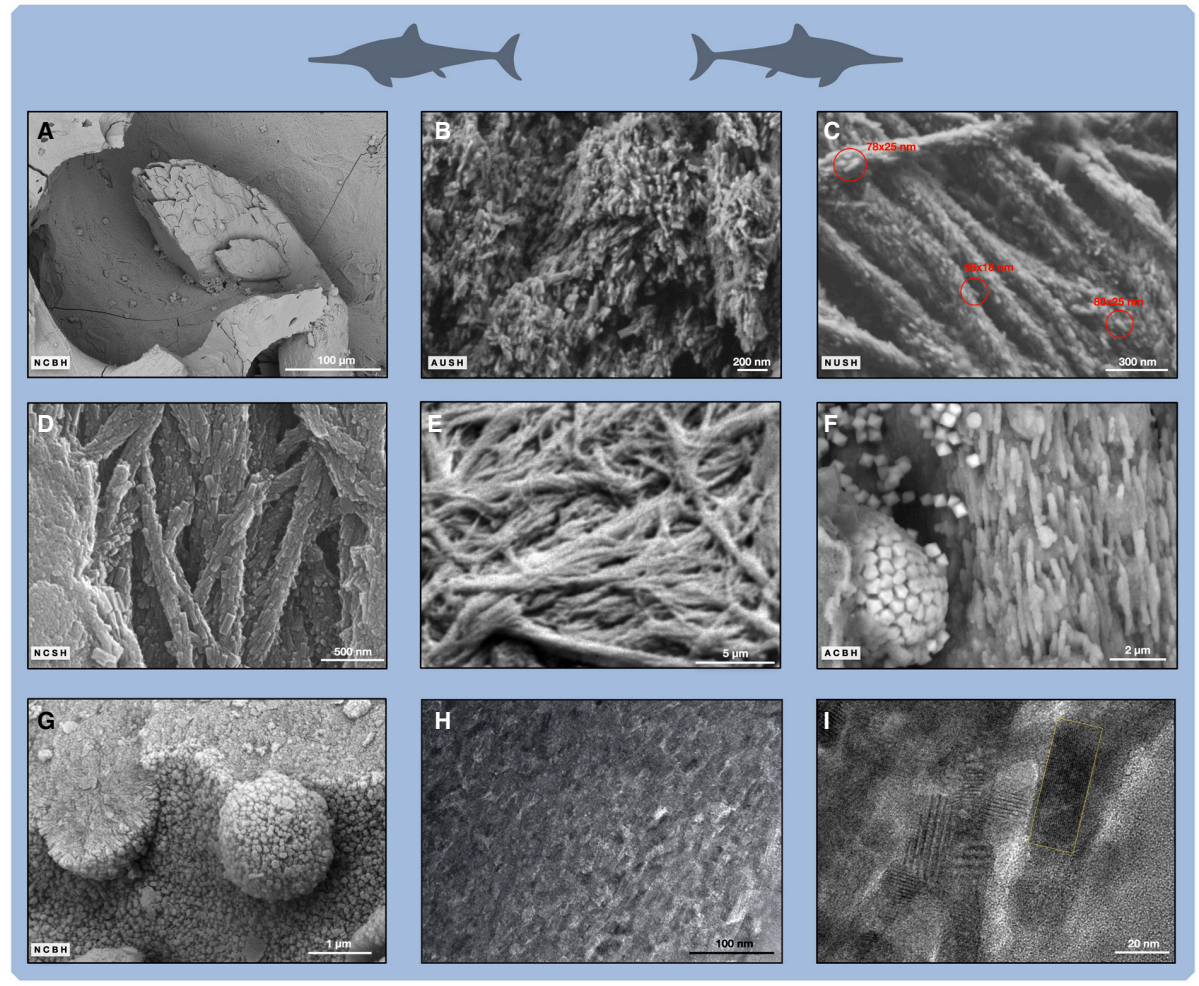
SEM and TEM images of ichthyosaur fossil bone and cartilage fibrils (A) OV1, open trabecular space with idiomorphic calcite crystal. (B) OV2, forming surface with platelet-shaped nanocrystals. (C) OV1, small crystals on fibrils located on surface in open porosities. The uncoated sample avoids possible coating artifacts, which might obscure or change the apparent dimensions of the nanocrystals. (D) OV1, larger nanocrystals on fibrils from trabeculae that collapsed during fossilization. (E) Human bone fibrils made anorganic with NaOCl. Note that the fibril surface is smooth. (F) LR1, mineralized cartilage fibrils and pyrite framboid precipitated during fossilization. (G) OV1, nanocrystals in cartilage area precipitated during fossilization. Nanocrystal size is similar to (D). Note the hexagonal shape of the nanocrystals. (H) OV2, TEM image of FIB section of fossil bone apatite crystallites. (I) OV2, TEM image of FIB section, close-up of fossil bone apatite crystallite (yellow rectangle). Parallel lines are the crystal lattice planes. Scale bar in (A) equals 100 μm; scale bar in (B) equals 200 nm; scale bar in (C) equals 300 nm; scale bar in (D) equals 500 nm; scale bar in (E) equals 5 μm; scale bar in (F) equals 2 μm; scale bar in (G) equals 1 μm; scale bar in (H) equals 100 nm; scale bar in (I) equals 20 nm.

At low SEM magnification, untreated and uncoated samples of OV1 (Figures 6A and S3) show fossilized bone fibrils with a rough surface and sometimes an apparent d-band periodicity (Figure S30A). This periodicity of 64–67 nm is characteristic of collagen and observed in modern bone fibrils, both in transmission electron microscopy (TEM) sections and in SEM investigations of bone made anorganic.^37^

At high magnification in the SEM, the fossilized fibrils appear to consist of arrays of nano-sized elongated apatite crystals whose mean size is around 65 × 20 nm, with an aspect ratio of around 3 (Figures 6C and S30B). These small elongated apatite crystals are more or less outwardly directed, but with a general mean orientation well aligned with the main fibril axis. Nano-sized apatite crystals in OV1 vary in size: well-preserved inner bone surfaces with open porosity have roughly half the crystal size (mean: 66 × 19 nm) than those from inner surfaces of millimeter-sized trabecular fragments resulting from trabecular collapse. There, the mean crystal size is 124 × 40 nm (Figures 6D and S31A). Both types of preservation show the same aspect ratio of 3.

In the larger elongated nanocrystals covering the fibrils, a hexagonal cross-section can be observed (Figure S30A). On the forming surfaces, there are also nanocrystals, but with a platy crystal habit (Figures 6B and S32). These elongated nanocrystals are quite different in shape and larger than the 2-nm to 5-nm-thick mineral phase often described from modern bones.^38^^,^^39^^,^^40^^,^^41^

In focused ion beam (FIB) sections of trabecular bone tissue of OV2 examined with the TEM, we observed a homogeneous mass of nanocrystals 50 × 20 nm in size (Figures 6H and 6I). This size is somewhat smaller than the nanocrystals on the outside of the fibrils but similar to what has been reported in TEM sections of modern bone.^42^^,^^43^ No space between crystals formerly taken up by collagen is apparent. TEM sections of fossil dinosaur bone^42^^,^^43^ also showed the lack of collagen but larger nanocrystals. These were the size of the largest nanocrystals observed by SEM in this study. The small and uniform size of the nanocrystals in OV2, is thus consistent with its excellent preservation in the SEM.

The preservation of the mineralized collagen fibers in the ichthyosaur cartilage (Figures 5D, 6F, S23B, and S33) raises similar questions as to their material nature. The collagen fibers appear to have been replaced by apatite in calcified areas but seem to have disappeared in uncalcified areas. EDX in the SEM shows a significant F content, also indicating a carbonate-rich fluorapatite composition (Figures S34–S37; Tables S3 and S4). In living cartilage, the matrix between the collagen fibers (mineralized as hydroxyapatite and unmineralized) is filled by proteoglycans with a high water content. In the fossils, and in the calcified cartilage of living species, this matrix space is mineralized by apatite as well (Figures 6G and S38–S43; Tables S1 and S2).

## Discussion

### Implications of differences in bone cell activity in the four ichthyosaur samples

In the trabecular bone surfaces of human lumbar vertebrae,^44^ the simultaneous presence of forming, resting, and resorption surfaces with a coupling of forming and resorption surfaces, a preponderance of resting surfaces, and an organization of fibrils into ordered domains characterizes the bone (re)modeling that occurs in healthy young adults, where bone mass is maintained during (re)modeling. However, in older people with osteoporosis, the proportion of resting surfaces is reduced and the majority of surfaces are either resorption surfaces or arrested mineralization front surfaces. There are also areas where the fibrils are more disorganized in 2D.^30^^,^^31^^,^^44^

If we were to compare the bone cell activity present in the four ichthyosaur specimens with what is known about remodeling in mammals, and in particular in humans, we would conclude that OV2, AB1, and LR1 exhibit the classic remodeling activity characteristic of relatively young and healthy animals, albeit with reservations given the significant phylogenetic gap separating these two groups. This is not the case for ichthyosaur OV1, whose remodeling activity appears atypical. The presence of numerous domains with disorganized fibrils and the imbalance between forming and resorption surfaces with the notable absence of forming surfaces are partly similar to features seen in older osteoporotic humans. Such approaches and conclusions were previously not possible because trabecular surfaces cannot be observed in the light microscope. Our SEM observation on OV1 could indicate either a sick animal whose bone remodeling had stopped, or a biological feature specific to ichthyosaurs, such as a seasonal cessation of bone remodeling. In the latter case, such patterns could be used to determine the season of death.

### No collagen in bone fibrils but preservation of the mineral skeleton

Despite the abundance of bone in the fossil record of the last 470 Ma,^45^ the process of its fossilization is poorly understood. Observation of petrographic thin sections in polarized light reveals that bone histology is almost always well preserved, independently of the age of the fossil.^1^ Also, by careful digestion with weak acids, fossil bone tissue up to 290 Ma old will commonly release osteocytes, blood vessels, and extracellular matrix, albeit in a degraded state.^46^^,^^47^ This preservation of organics in bone tissue may have inspired the interpretation that fossil fibrils represent the mineralized collagen fibrils,^4^^,^^6^^,^^7^ retaining some or all of the collagen, either as the preserved macromolecule or in a degraded organic form. We question this interpretation and hypothesize instead that the fossil fibrils only correspond to the mineral skeleton of bone fibrils without any organic remains. Future work using methods of molecular paleontology^4^^,^^5^^,^^6^^,^^7^^,^^35^^,^^46^^,^^47^ will test our hypothesis.

Based on our extensive SEM-based observations, we also question whether any of the fossil fibrils represent originally unmineralized collagen fibrils that were mineralized during fossilization. Fibrils originally not embedded in a mineralized matrix are not preserved in our samples: thus, osteoid collagen fibrils are not preserved (see the similarity of the fossil forming surfaces and the modern deproteinized ones (Figures 2A and 2B)), nor are segments of Sharpey fibers crossing cavities originally filled with bone marrow between two bone trabeculae (Figures 2I and 2J). In addition, no unmineralized collagen fibrils are preserved in the fossil cartilage, unless they are associated with calcospherites (Figures 2L and 5D). We observed fibril preservation only in or near mineralized cartilage matrix but not in matrix where the cartilage was not mineralized *in vivo* (Figures 2L, 5D, 5E, and S22–S24). Whereas our interpretation disagrees with previous interpretations emphasizing the exceptional preservation of collagen,^4^^,^^5^^,^^6^^,^^7^ it offers an explanation for the enhanced birefringence in thin sections in polarized light of fossil bone compared to modern bone.^1^

### Cells, their secretions, and activity traces inform on the evolution of bone

Although the principle of uniformitarianism and, more specifically, phylogenetic inference,^48^^,^^49^ would suggest that the cellular mechanisms of bone formation are ancient, direct proof had been lacking. Our observations confirm that the cellular mechanisms of periosteal and endochondral bone formation and resorption, i.e., the basics of bone growth and turnover, were exactly the same 200 million years ago as they are in living amniotes. We posit that the underlying molecular mechanisms were the same in the Jurassic as they are today. Given that fossil bone preservation has varied little since the origin of bone as a tissue 460 million years ago, our work opens up perspectives on a deeper understanding of the evolutionary origin of amniote osteogenesis and vertebrate osteogenesis in general in the Silurian and Devonian.^50^^,^^51^ In this study, we provide a paleohistological approach useful for the comparative study of ancient fossils, which complements both traditional light microscopy techniques and emerging 3D observation methods. Our approach allows in-depth study of the fibrillar organization and cellular activity of fossil bones and offers a direct link between bone from deep time and the vast field of biomedical research on bone.

### Limitations of the study

Given the great temporal spread and different geological backgrounds of our samples (Table 1), we suggest that the kind of preservation we document is widespread, if not the norm, in fossil bone from deep time. However, testing this hypothesis requires broad sampling across the vertebrate fossil record, which we yet have to conduct. Whereas we have gained a clear comparative understanding of the naturally deproteinized inner bone surfaces (forming, resting, and resorbing) down to the bone fibrils of the fossils, we do not yet fully understand the interior structure of the fossil bone fibrils and of the fossil bone tissue below the fibril level of integration. We and others have imaged fluorapatite crystallites via TEM inside fossil bone tissue,^42^^,^^43^ but we do not know what the relationship of these crystallites is with the bone fibrils and the space in between fibrils. Given the ubiquitous observations in polarized light microscopy of fibril preservation in fossil bone,^1^ we must assume that the apatite crystallites are somehow related to the original bone fibrils. We now have the techniques to address this question with spatially controlled FIB-SEM sampling followed by TEM investigation. Although fossil cartilage has consistently been observed with the light microscope across the amniote tree and deep time,^34^ it is clear that cartilage fossilization must be more complex than bone fossilization because of the more variable degree of mineralization of cartilage vs. bone. The conclusions we have drawn from the differences in bone cell activity are currently limited and need to be confirmed. This is due to the paucity of data in the literature, requiring more SEM observations of systematically and temporally diverse fossil specimens. Furthermore, our observations are currently of a qualitative nature. Quantification will employ automated SEM imaging to cover large fields of view with sufficient resolution to detect fibril organization combined with automated image analysis.

## Resource availability

### Lead contact

Further information and requests for resources relating to this manuscript should be directed to and will be fulfilled by the lead contact, P. Martin Sander (m.sander@uni-bonn.de).

### Materials availability

The fossil specimens investigated in this study as well as the sections and SEM stubs are accessioned to the vertebrate paleontology collections of Paleospace Museum in Villers, Normandie, France (see Table 1). All comparative modern samples are kept in the lab of A.B. (see Table 2). All other data are available in the manuscript or the supplemental information.

**Table 2:** Preparation parameters of SEM samples of modern skeletal tissues

Figure	Cleaning	Drying method	Species & bone	Subject matter
Figure 1A	wet dissection	critical point CO_2_	rat calvarium	osteoblasts *in situ*, side away from bone matrix
Figure 1B	dry stripping on adhesive tape	already dry	rat calvarium	osteoblasts *in situ*, side facing bone matrix
Figure 1C	none, fractured	air dried from amyl acetate	monkey femur	osteocyte in lacuna
Figure 1D	PMMA cast exposed by HCl and NaOCl	air dried	human incus	osteocyte lacunae and canaliculi
Figure 1E	cells removed by air blasting when dry	critical point CO_2_	human iliac crest biopsy	adipocytes as bone lining cells
Figure 1F	wet dissection	critical point CO_2_	rat calvarium	osteoclast *in situ*
Figure 1G	wet dissection	critical point CO_2_	rat calvarium	liberated osteocytes moving out of their lacunae
Figure 1H	ethanol freeze fracture	critical point CO_2_	rat femur growth plate proliferative zone	chondroblasts, mitotic twins
Figure 1I	ethanol freeze fracture	critical point CO_2_	rat femur growth plate hypertrophic zone	hypertrophic chondrocytes
Figures 2A, 2C, 2E, and 2G	made anorganic with 7% NaOCl for 5 days followed by boiling in distilled water for 10 min.	bone recovered on the coast and bleached by the sea and the sun for an indeterminate time	porpoise vertebra	forming, resting, and resorption surfaces, bone fibrils
Figure 2I	tissue sample embedded in PMMA, cut and polished, surface stained with iodine vapor	tissue fixed in buffered glutaraldehyde, dehydrated with ethanol, substituted with methyl methacrylate monomer, then polymerized to PMMA	deer antler	Sharpey fibers inserting on inner surface of outermost bony trabecula
Figure 2K	made anorganic with 7% NaOCl buffered by a saturated NaHCO_3_ solution for 14 days	bone recovered on the coast and bleached by the sea and the sun for an indeterminate time	porpoise vertebra	cartilage calcospherites
Figure 6E	made anorganic with NaOCl	air dried from ethanol	human	bone fibrils at high magnification
Figure S2A	made anorganic with NaOCl	air dried from ethanol	mouse	forming surface
Figure S2B	not anorganic, cleaned with Water Pik	air dried from ethanol	human	bone fibrils on the surface of trabecula

A comparative sample set of modern skeletal tissues was figured in our study as listed.

### Data and code availability

• The datasets generated and analyzed related to this paper are available from the lead contact on reasonable request.
• This paper does not report original code.
• Any additional information required to reanalyze the data reported in this paper is available from the lead contact upon request.

## Acknowledgments

We thank members of the DFG Research Unit FOR 2685 “Fossilization” for discussion. Comments by Timothy Bromage (New York City) on an earlier version of the manuscript greatly improved it. Formal reviews by Paul Ullmann (Glassboro, NJ) and an anonymous reviewer are much appreciated. R.-P.E. wishes to thank ARKEMA for allowing him to combine his professional work with his passion for paleontology through an 80% position. This is contribution no. 63 of DFG Research Unit FOR 2685 “Fossilization.” This study was funded by 10.13039/501100001659German Research Foundation grant Sa 469/54-1 as part of FOR 2685 (P.M.S.), 10.13039/501100001659German Research Foundation grant Sa 469/55-2 as part of FOR 2685 (P.M.S. and PIs of FOR 2685), 10.13039/501100000266Science Research Council UK (A.B.), 10.13039/501100000265Medical Research Council UK (A.B.), 10.13039/501100000266Science and Engineering Research Council UK (A.B.), 10.13039/100010269Wellcome Trust UK (A.B.), and Veterinary Advisory Committee of the Horserace Betting Levy Board (A.B.).

## Author contributions

Conceptualization, R.-P.E., A.B., and P.M.S.; methodology, R.-P.E., A.B., X.J., and P.M.S.; investigation, R.-P.E., P.M.S., A.B., and X.J.; visualization, R.-P.E., P.M.S., A.B., and X.J.; funding acquisition, P.M.S. and A.B.; project administration, P.M.S.; supervision, P.M.S., A.B., and R.-P.E.; writing – original draft, P.M.S. and R.-P.E.; writing – review and editing, P.M.S., R.-P.E., A.B., and X.J.

## Declaration of interests

The authors declare no competing interests.

## STAR★Methods

### Key resources table

**Table:** 

REAGENT or RESOURCE	SOURCE	IDENTIFIER
**Biological samples**		

Amniote modern skeletal tissues	Table 2	N/A

**Chemicals, peptides, and recombinant proteins**		

10% phosphoric acid	STAR Methods, section "acid treatment protocol for fossils"	N/A
10% phosphoric acid	STAR Methods, section "acid treatment protocol for fossils"	N/A
8% acetic acid solution	STAR Methods, section "acid treatment protocol for fossils"	N/A
Ethanol	Table 2	N/A
HCl	Table 2	N/A
7% NaOCl buffered by a saturated NaHCO_3_ solution	Table 2	N/A
Polymethyl methacrylate (PMMA)	Table 2	N/A

**Deposited data**		

Jurassic ichthyosaur fossils	Table 1	LR1 (MPV 2024.1.1), OV1 (MPV 2024.1.2), OV2 (MPV 2024.1.3), AB1 (MPV 2024.1.4)

**Software and algorithms**		

Software "Orientation J"	Sage, D. (2023). OrientationJ. A series of ImageJ plugins for directional image analysis (Lausanne: Biomedical Image Group (BIG), EPFL, Switzerland).	N/A

### Experimental model and study participant details

This study did not use experimental model animals, experimental *in vivo* animals, human participants, plants, microbe strains, cell lines, or primary cell cultures.

### Method details

#### Specimens

We here investigate vertebral centra from four different ichthyosaur individuals that also represent four different species and three different ontogenetic stages, from early juvenile to skeletally mature adult (Table 1). The ichthyosaur fossils were collected at the Normandy coast of France and the Dorset coast and Oxfordshire in England (see below). All fossils derive from dark impermeable mudstone. The specimens investigated in this study as well as the mounted SEM samples are accessioned to the vertebrate paleontology collections of the Paleospace Museum in Villers, Normandie, France (see https://www.normandie-cabourg-paysdauge-tourisme.fr/en/patrimoine-culturel/le-paleospace-musee-de-france/).

The ontogenetically youngest (but geologically oldest) specimen is **LR1**, which is from the famous Early Jurassic locality of Lyme Regis, Dorset, UK. The specimen derives either from the Hettangian-Sinemurian Blue Lias Formation or the overlying Charmouth Mudstone Formation of entirely Sinemurian age. LR1 consists of an isolated middle caudal vertebral centrum lacking the neural arch. The neurocentral suture is always open in ichthyosaurs, leading to frequent taphonomic loss of the arch.^52^ The caudal position is indicated by the ventral facets for the hemapophyses and the single rib articular facet. In Jurassic ichthyosaurs, all presacral vertebrae have double rib articular facets.^52^ Given the relatively large size for an early juvenile and the middle caudal position, the centrum probably does not belong to *Ichthyosaurus* but the larger *Temnodontosaurus*. However, assignment to any of the many named species is currently not possible. LR1 was obtained from a fossil dealer in the UK (Margaret Kate Lawlor, Cirencester. https://www.ebay.de/str/numismatist48).

The two French Late Jurassic (Kimmeridgian) fossils (**OV1** and **OV2**) derive from two partial ophthalmosaurid ichthyosaur skeletons pertaining to two different species based on size in conjunction with ontogenetic stage of the vertebral centra. The two specimens were collected and prepared by one of the authors (R.P.E.). The locality is the coastal outcrops at Octeville near Le Havre, Normandie, France (see below for details).

**OV1** is a disarticulated, fragmentary skeleton of a medium-sized ichthyosaur. Given its Late Jurassic geologic age, the specimen very likely pertains to an ophthalmosaurid.^53^ The specimen consists of various disarticulated skull bones, including both lower jaws, and a few vertebral centra from the cervical to the posterior dorsal region. Additional bones may have been lost to weathering on the beach or may still be covered by overburden. For this study, we used one cervical vertebral centrum of this individual. The position of this centrum in the column is indicated as cervical by the contact of the diapophysis (the dorsal of the two rib articular facets) with the neural arch.^54^ Histological examination indicates the individual was still actively growing at the time of death (see below).

The second specimen (**OV2**) is similarly a fragmentary associated skeleton of a presumed ophthalmosaurid, albeit of markedly smaller body size. Only a series of dorsal to anteriormost caudal centra was recovered from this skeleton. Its stratigraphic horizon is slightly older than that of OV1 (see below). The single vertebra studied here is a posterior dorsal as indicated by the positions of the diapophysis and parapophysis.^54^ Histological examination of OV2 indicates that it had reached skeletal maturity (see below).

For the size comparison of the two French specimens, we used the posterior dorsals. Determination of the location in the column is based on the position of the diapophysis being located just above the dorsoventral midline on the side of the centrum. The dimensions of the single preserved anterior dorsal centrum (diapophysis just above midline) of OV1 are as follows: transverse width 57 mm, dorsoventral height 51 mm, and anteroposterior length 20 mm. Note that these are approximate values because the centrum is slightly deformed. As noted, OV2 is markedly smaller (Table 1), and the dimensions of an anterior dorsal centrum (diapophysis just above midline) of similar shape (but not sampled) to that of OV1 are as follows: transverse width 39 mm, dorsoventral height 36 mm, and anteroposterior length 19.5 mm. Thus the transverse width and the height of the centrum of OV1 is about 1.45 times larger than that of OV2, but the difference in length is much less marked, indicating different proportions of the OV1 and OV2 anterior dorsal vertebral centra.

The marked difference in size of OV1 and OV2, which would even have been greater if OV1 had reached skeletal maturity, combined with the difference in proportion and the difference in geologic age, require a biological explanation. The most likely is that the two individuals represent different species of ophthalmosaurids, slightly separated in time. Sexual dimorphism is unlikely as an explanation, because none has been detected in any Jurassic ichthyosaur species despite the large number of gravid females known, and because both the size difference and the proportional difference are too pronounced for sexual morphs. Because of the proportional differences, we can also rule out developmental plasticity and two populations of the same species separated in time. Because of the incompleteness of the material, we refrain from assigning the two specimens to any named species of ophthalmosaurid.

The fourth specimen (**AB1**) is a relatively large posterior dorsal centrum from the Kimmeridge Clay Formation of Abingdon, Oxfordshire, UK. The posterior dorsal position of the centrum is based on the position of the diapophysis being located just above the dorsoventral midline on the side of the centrum.^54^ The centrum is well preserved and undeformed. Based on its large size and ontogenetic stage (late juvenile, see below), it cannot represent the same species as either of the French vertebrae (OV1 and OV2). Also, Ophthalmosauridae are virtually the only Kimmeridgian clade of ichthyosaurs,^53^ hence the assignment to this clade, and it is highly unlikely that the material represents any other kind of ichthyosaur. AB1 was obtained from a fossil dealer in the UK (Megalodon and More Fossils Ltd., Buxted. https://fossilsforsaleuk.co.uk/).

#### Macroscopic preservation of the fossils

LR1 is little deformed by compaction but is abraded, which either may have happened before burial or after exposure on the beach at Lyme Regis. Sectioning revealed that diagenetic pyrite filled some of the porosity, whereas other parts were filled by calcite. Pyrite is a common pore space filling component in Mesozoic marine reptile bones^55^ as the first generation of diagenetic mineral precipitation during fossilization.

In OV1, bone preservation at the macroscopic level is variable across the skeleton and even within a single bone. Bones, in particular centra of OV1, may have completely or partially open porosity (Figure 4A). In this case, the centra are deformed by sediment compaction in the region of the open porosity. Other parts of the centra, including the sampled one, have the porosity filled in by calcite (Figures 3A, 4B, and 5H). The bones of the lower jaw also have some open porosity, but much of the pore space is filled in by diagenetic calcite. Microscopic observation, both in petrographic thin sections and SEM sections, reveals some trabecular collapse to be associated with the deformation (Figure 5H). In an advanced stage of collapse, the centra appear deformed and compressed from the outside. The inside of these bones is filled with loose bone powder which represents broken-up trabeculae. In petrographic thin sections (see below), it can be seen that at least some of the calcite precipitation occurred after trabecular collapse, indicating relatively late cementation.

Although one might hypothesize that the open porosity in the fossils is the result of recent weathering, this is unlikely because the fossils are embedded in an impermeable mudstone and exhibit the open porosity in bones that were not naturally exposed. Recent weathering as the cause for the open porosity also is unlikely because the collapse occurred before exposure by shore face erosion and in particular before the diagenetic calcite precipitation. In addition, some open porosity shows well-formed idiomorphic calcite crystals in the SEM, extending into the open space (Figures 6A and S3), also indicating that the porosity is primary. All the centra of OV2, on the other hand, are not deformed macroscopically, and the porosity is always filled with calcite (Figure 3A). Similarly, AB1 is undeformed with good bone surface preservation. It, too, shows complete infill of the porosity with calcite.

#### Osteohistology and ontogeny of ichthyosaur centra

As in many fully marine tetrapods, ichthyosaur vertebral centra entirely consist of cancellous bone^56^^,^^57^ but preserve most bone matrix types and bone tissue types known from other amniotes, both from the periosteal and endochondral territories.^56^^,^^57^ Osteohistological terminology follows.^21^

As a background for understanding ichthyosaur centrum microanatomy and histology, a review of centrum morphology is in order. Ichthyosaur vertebral centra are typically very short and disk-like, their transverse diameter commonly being at least twice their anteroposterior length. The centra are deeply amphicoelous but not notochordal in adults. Dorsally, the centra preserve the floor of the neural canal flanked by the facets for the neural arch. One or two rib articular facets are found laterally on the centrum. Facet number and arrangement are of taxonomic and positional value.

Both sagittal and transverse sections are needed to understand the morphogenesis of the centra from the osteohistological perspective and the contributions of the periosteal and endochondral developmental territories to bone tissue formation.^56^^,^^57^ In the periosteal territory, bone matrix is directly deposited by osteoblasts, whereas growth in the endochondral territory is accomplished by cartilage deposition, which then is replaced by bone tissue.^58^ Sagittal sections of ichthyosaur centra have an hour-glass shape and reveal the mechanisms of centrum growth both in anteroposterior and dorsoventral directions and the nature of the articular surfaces between the centra.^34^^,^^56^^,^^57^ Transverse sections reveal the contribution of each developmental territory to the rib articular facets, the floor of the neural canal, and the facets for the neural arch.

In sagittal section, it can be seen that dorsoventral growth is accomplished by periosteal bone deposition, and that the increase in diameter of the centrum is accompanied by an isometric increase in anteroposterior length (Figures 3A and 5A). Bone tissue formation in the endochondral territory is much slower and lags behind periosteal growth, leading to the double-funnel shape of the centrum. Importantly, all primary bone tissue is cancellous, and the cancellous nature thus is not the result of remodeling, unlike in terrestrial amniotes (including humans). Primary cancellous bone of the periosteal territory can be seen in three of our specimens, but its amount differs. In LR1, it is the dominant tissue whereas in OV1 and AB1, only the outer few millimeters below the bone surface are primary (Figure 4A), and remodeling had transformed most of the tissue in the periosteal territory to secondary trabecular bone. OV2 has few traces of primary trabecular bone left in the periosteal territory. LR1 thus is ontogenetically youngest and OV2 is oldest. This pattern is best understood by applying the Three Front Model.^59^

The primary trabecular bone of LR1, OV1, and AB1 is made up of woven-fibered bone tissue with some lamellar bone lining the trabecular spaces. In effect, the primary trabecular bone of these (and other) ichthyosaurs thus is a woven-parallel complex,^56^^,^^57^ a combination of non-scaffolded woven bone plus scaffolded bone.^21^

In the endochondral territory, the specimens differ in the thickness of the columnar cartilage layer, i.e., the amount of cartilage that has not yet been transformed to bone. Again, there is a gradient from LR1 with much cartilage (i.e., many cells per column) (Figure 5B) via OV1 and AB1 (Figure S24) to OV2 which has little cartilage. In comparison with ichthyosaur vertebral centra of known ontogenetic stage,^56^^,^^57^ LR1 is an early juvenile, possibly even neonatal or fetal) and OV1 and AB1 were late juveniles, still actively growing at the time of death. In OV2, growth had stopped, indicating it to be a mature individual. However, ichthyosaurs are different from other amniotes in that the neurocentral suture never fused, representing a case of extreme pedomorphosis. Even in adult and old ichthyosaurs, the neural arches are seen to separate from the centrum postmortem.^52^^,^^54^

In the transverse sections, the close link between territories and morphogenesis is also apparent. Bone tissue of the periosteal territory is sharply set off from that of the endochondral territories even in the adult, OV2. This is typical for ichthyosaurs^56^ and reflects the preservation of territorial identity despite extensive remodeling. Starting at the growth center, the tissue of endochondral origin appears as four wedges, two of which extend dorsally and underlie the facets for the neural arch. Those two wedges are separated by a wedge of periosteal bone which underlies the floor of the neural canal. The other two wedges of endochondral bone extend out laterally from the growth center and underlie the rib articular facets^56^, specifically the diapophysis. Note that the endochondral tissue present in the four wedges grows at the same rate as the adjacent periosteal tissue, and therefore much faster than the endochondral tissue growing on the anterior and posterior faces of the centra. Cartilage of various thickness is preserved at the surface of all four wedges. Far from the outer bone surface, islands of mineralized cartilage that escaped remodeling are present in the endochondral bone tissue, mainly in areas of rapid cartilage growth in the four wedges of endochondral origin of juvenile specimens.

Especially ventrally, but also dorsally, there are Sharpey fibers in the primary periosteal bone close to the border with the endochondral territory. There are no fibers away from the border, toward the middle of the periosteal surface (Figure 5H). The fibers parallel this border and represent the insertion of the joint capsule into the periosteal bone^34^ (Figure 5H). These fibers are destroyed by remodeling activity and are not seen in the secondary trabecular bone.

Our ichthyosaur sample thus covers the whole range of periosteal and endochondral bone tissue types and other features seen in amniotes. This is important because it gave us the opportunity to study all of those tissues and features in the SEM.

#### Geological setting

The foreshore coastal locality of discovery of OV1 and OV2 is below the village of Octeville near Le Havre (Normandy, France). The deposit of origin is the Kimmeridgian Argiles d’Octeville Formation,^60^ and the fossils come from two different horizons within this formation. Both of these are black claystone incorporating numerous flattened and crushed ammonites and bivalves. OV1 comes from higher in the section than OV2, from the *Membre Supérieur des Argiles d’Ecqueville*, in the *Eudoxus* Zone. In this horizon, we observed molluscs, in particular ammonites, preserved with iridescent nacreous shells. The horizon of OV2 lacks such superb preservation and very probably correlates with the *Membre Supérieur des Argiles du Croquet* in the upper part of the *Mutabilis* Zone. The two discovery units are separated by the *Membre Median des Argiles d’Ecqueville* and the *Membre Inferieur des Argiles d’Ecqueville*. Both discovery units are of late Kimmeridgian age.^60^^,^^61^ The numerical age of the fossils is thus about 153 million years.

Details of the geological setting of the two UK specimens obtained from fossil dealers are not known. This is not crucial, however, because both the localities of Lyme Regis and Abingdon are well known in general. LR1 derives from the early part of the early Jurassic, either from Hettangian or Sinemurian dark mudstones, both of which crop out on the foreshore and in the cliffs around Lyme Regis and Charmouth, immediately to the east.^62^ Abingdon, on the other hand, is a well known locality for fossils of the Kimmeridge Clay Formation, also of Kimmeridgian age,^63^ but the horizon of origin cannot be constrained further for AB1.

#### Sample sectioning

To allow observations of the internal bone tissue by SEM, the vertebrae had to be sectioned in different planes. The sectioned plane, however, was not the main focus of observation, but the naturally occurring internal trabecular surfaces. Because of the remarkable preservation of the Octeville ichthyosaur bones, only limited sample preparation was necessary. Initially, a few millimeter-sized fragments with open porosity were taken from the broken parts of the vertebral centra or were intentionally broken from larger pieces at defined locations, but it was soon recognized that controlled planes of sections were needed to properly understand bone tissues. Thus, all vertebral centra of OV1 and OV2, LR1 and AB1 were sectioned with a rock saw and then ground and polished with 1000 grit to obtain a smooth surface. However, before sectioning, the regions to be cut from the vertebra of OV1 were embedded with green epoxy putty (Kulzer Technovit) to protect the edge of the sections (e.g., Figure 5H). This was not necessary in the more indurated specimens OV2, LR1 and AB1. OV1 was sectioned with a Buehler automatic rock saw. OV2, on the other hand, was sectioned with a water cooled Buehler Isomet low-speed saw, which also minimized damage to the edges. Finally, LR1 and AB1 were sectioned with a standard lapidary saw (Hi-Tech diamond 4’’/5″ Trim Saw) operating at a speed of 800 rpm.

Planes of section were parasagittal in the case of the vertebral centra of OV1 (Figure 5H) and OV2 (Figure 3A), with an additional transverse section through an articular facet in the case of OV1 (Figure 5A). Centrum LR1 was also sectioned parasagittally through one facet for the neural arch, and centrum AB1 was sectioned sub-transversely (slightly diagonally to the transverse plane) to intersect one rib articular facet. We chose parasagittal sections of the centra because such sections (Figure 3A) intersect both the periosteal and endochondral territories. Intersecting a rib articular facet as part of a transverse section reveals the cartilage of the rib joint and the underlying endochondral bone. Because of the deeply amphicoelous nature of ichthyosaur vertebral centra, sections need to be placed exactly in the sagittal plane to avoid incomplete tissue coverage.^34^ However, because we wanted to obtain more than one sagittal section from some centra, the second section plane will necessarily have incomplete coverage.

To cross-check the SEM observations with polarized light microscopy, the standard technique in paleohistology, petrographic thin sections were produced from OV1 from the other half of the sampled vertebrae not sectioned for SEM. The petrographic thin sectioning technique generally followed reference.^64^

#### Acid treatment protocol for fossils

Initial SEM observations were done without any further treatment in open-porosity samples of OV1 (e.g., Figures 4A, 6A, S2, and S3). However, since some regions, like the endochondral area on the ventral side of the parasagittal section of OV1 (Figures 3A and 5H), showed a diagenetic calcite infill, its removal appeared advisable. Calcite removal was achieved with a 8% solution of acid acetic in water overnight and was found to remove the calcite from the pore space and reveal the same surface features with the same quality as in the areas with the primary open porosity (e.g., Figures 3H and 3I). The sampled vertebra of OV2 had a complete calcite infill of its porosity which also was removed with 8% acetic acid overnight. This method was also applied to LR1 and AB1. Because of the abundant pyrite in the former, less trabecular surface could be exposed, the pyrite completely covering some internal bone surfaces. Removal of the calcite also exposed sparse diagenetic pyrite octahedra and framboids that were precipitated on the trabecular surfaces during fossilization (e.g., Figures 2H, 3F, 3H, 4H, 5J, and 6F). The pyrite thus serves as a convenient reminder that we are looking at fossils and not at modern bone.

The 8% acetic acid solution does not attack the bone apatite on the polished sections intersecting the bone microanatomy, such as the bone trabeculae. These polished cutting planes, like the fracture surfaces, remained featureless in the SEM except for open osteocyte lacunae (e.g., Figures 4F, S10, and S16). In order to visualize fibril orientation on these section planes, we tried etching with dilute phosphoric acid (20% for 2 to 6 s and 10% for 3 s). During immersion in the phosphoric acid, the sample was shaken, and then rinsed very quickly under running water to limit salt redeposition on its surface. While this worked well, the phosphoric acid also attacked the internal bone surfaces. To avoid this problem, we developed a two-step protocol for specimens with a calcite porosity fill in which the calcite fill temporarily shields the internal bone surface from the phosphoric acid. Therefore, after cutting and polishing, we first etched the section surface with phosphoric acid as detailed above, then removed the remaining calcite that filled the pores with 8% acetic acid overnight to reveal the internal bone surfaces. This way, we were able to observe fibril patterns as a continuum from the sectioned surfaces to the inner bone surfaces (e.g., Figure S4).

The areas of naturally open porosity in OV1 had to be observed before any phosphoric acid etching because its etching also affected the internal trabecular surfaces. Future protocol improvements should involve a removable artificial compound for filling the naturally open porosity before phosphoric acid etching and removing the protective compound before SEM observation. The type of acid treatment is indicated by the first position of the image code (see below) accompanying each SEM micrograph.

#### Effects of phosphoric acid etching on sectioned surfaces

At medium magnification in the SEM of the fossil ichthyosaurs, fibril structures are imaged that are continuous from the internal bone surfaces to phosphoric acid-etched polished and fracture surfaces (Figure S4). These fibril structures can be related to transmitted light observation of thin sections. Features brought out by etching are bone fibrils, but also reversal and cementing lines, i.e., resorption surfaces preserved inside the bone tissue (Figures 3G, S4A, and S9A).

Bone fibrils are not as clearly visible on the etched surfaces as are the fibrils on the free inner surfaces (Figure S4A). Also, the fibril arrangement is more difficult to detect on the etched surfaces. There are several reasons for this: first, fibril diameter after etching is smaller than that of the fibrils observed directly on the internal trabecular surfaces without etching (Figure S3A). So far, in the SEM we have only observed the fibrils on etched surfaces after Au/Pd coating, and we were not able to resolve their fine structure. Second, depending on the angle with which the fibrils intersect the fracture or polished section surface, they may appear as short fibril segments or as small ‘dots’ rather than as continuous features (Figures S4A and S9B). Third, etching with strong acid may result in undesirable local phosphate mineral reprecipitation during or at the termination of the etching process. This reprecipitation can obscure the surfaces to be studied. In addition, etching leads to pit artifacts that enlarge existing holes and, in particular, cracks (Figure S4A).

Etching also reveals the interface between islands of mineralized cartilage and bone in etched sections (Figures 5C, 5E, S4, S24, and S26). If an internal trabecular surface was accidentally exposed to phosphoric acid, the fibrils also appear continuous, but with a similarly reduced diameter, as in the polished and then etched sections.

In conclusion, since the internal structure of the bone and cartilage matrix is not apparent in natural fracture surfaces and section surfaces, phosphoric acid may be used to reveal them. However, as in any etching process of mineralized tissues, artifacts always need to be taken into account, unlike in the preserved natural internal surfaces that in the case of the open bone porosity do not require any treatment for SEM observation at all.

#### SEM observations and EDX analysis of fossils

Our SEM work encompassed the generation of over 1000 images at different magnifications for a comprehensive coverage. Some of the samples were left uncoated but most were coated with ∼10 nm of Au/Pd. Coated samples mainly were observed at medium magnification under high vacuum with a Quanta 250 field emission (FEG) SEM manufactured by FEI, a subsidiary of Thermo Fisher Scientific. The FEI Quanta 250 SEM is equipped with two detectors, an Everhart-Thornley secondary electron (SE) detector and three backscattered electron (BSE) detectors, all arranged in an annular configuration. The different BSE detectors are abbreviated as ABS, CBS, and GAD in the SEM image data bar. Samples were observed at accelerating voltages between 10 and 20 keV.

Uncoated samples of bone fibrils were imaged by SEM at high magnification under high vacuum with a Zeiss Merlin FEG SEM equipped with an in-lens duo detector and operated at an accelerating voltage of ∼2 keV. Uncoated samples were also imaged at lower magnifications and under low vacuum with the FEI Quanta 250 SEM at an accelerating voltage of 10 keV and a working distance of ∼10 mm.

To each SEM micrograph found in the main text and Figures S1–S33, we added an ‘image code’ in the lower left corner to summarize the imaging parameters discussed above. The code works as follows:

1st position, acid treatment: N/A/P, **N**o treatment/**A**cetic acid treatment/**P**hosphoric acid etching.

2nd position, sputter coating: U/C, **U**ncoated/**C**oated.

3rd position, SEM detector type: S/B, **S**econdary electron/**B**ackscattered electron.

4th position, vacuum: H/L, **H**igh vacuum (e.g., 1.8x10^−3^ Pa)/**L**ow vacuum (e.g., 80 Pa).

Thus, for example ‘N C B H’ indicates an image obtained from an untreated, but sputter-coated sample with a BSE detector at high vacuum.

Dimensions of the nano-sized crystals seen on the bone fibrils at high magnification in OV1 were measured manually with the ImageJ software using the straight line measurement tool (Figure 6C). The selected dimensions then were saved in ROI (region of interest) set files that are linked to the SEM images selected for these measurements. Approximately 20 length measurements and 30 width measurements were made on small crystals, selecting those with the best defined contours. The measurements are only approximate because they were not corrected for possible projection effects.

In order to better understand fibril orientation, SEM images were analyzed and colorized using the ImageJ plugin, OrientationJ^61^ (e.g., Figures 3G, 3I, S6, S8–S10, S12, S13, S15, and S19). This plugin gives a qualitative and quantitative analysis of the directionality of objects, here the fossilized mineralized collagen fibrils, in a digital image. The program computes the local orientation and the local coherency and creates a color map in the HSB model, where (H)ue is orientation, (S)aturation is coherency, and (B)rightness is the source image.^65^

EDX analysis in the SEM was done with an Oxford detector installed on both the FEI Quanta 250 and the Zeiss Merlin microscopes at 8 to 20 kV (see Figure S34–S53 and Tables S1–S4). Although most samples were coated with Au/Pd, this coating does not compromise the EDX results because of the elements we targeted (C, O, F, Na, P, S, Ca) and the low Au and Pd peaks in our spectra (see Figure S34–S53). It should be noted that the relative atomic compositions (or weight percentages) and relative intensities of EDX peaks are affected by the relief of the sample. Furthermore, the X-ray emission zones, particularly those of the K alpha lines of calcium and phosphorus, correspond to much larger areas both on the surface and at depth than the emission zone of the secondary or backscattered electrons, which is the origin of the SEM image. The EDX spatial resolution thus is lower than that of the SEM image.

#### TEM, EDS, and electron diffraction analyses of fossils

Samples for transmission electron microscopy (TEM) (Figures 6H and 6I) were obtained from OV1 and OV2 with a ZEISS Crossbeam 550L large chamber FIB-SEM workstation at the IEMN (Institute of Electronics, Microelectronics and Nanotechnology) at the University of Lille. The FIB lamellae were observed on a JEOL JEM 2100F TEM operating at 200 kV and equipped with a Gatan Ultrascan 1000 camera and an Oxford EDS spectrometer. Data were acquired in C-TEM, HR-TEM and Selected Area Electron Diffraction modes. The TEM is installed at the Centre Technologique des Microstructures of the University of Lyon.

#### SEM observations of modern skeletal tissues

The SEM images of modern skeletal tissues and cell types in Figure 1 and modern bone made anorganic in Figures 1A and 6E were produced in the lab of author A.B. using Cambridge Instrument Co. Stereoscan Mark I or S4-10 SEMs. All samples were coated with ∼10–20 nm gold by vacuum evaporation or sputtering. All images shown were recorded with 10 kV accelerating voltage using a biased scintillator Everhart-Thornley secondary electron detector.

Porpoise (*Phocoena phocoena*) vertebrae were recovered from the beach near Octeville, Normandie, France, by author R.P.E. The bones had been bleached by the sea and the sun for an indeterminate time before recovery. Porpoise samples were made anorganic (Table 2) and coated with ∼10 nm Au/Pd. SEM images were produced at 10kV accelerating voltage and a working distance of ∼10 mm with either the SE detector or one of the BSE detectors on the FEI Quanta 250 FEG SEM under high vacuum (e.g., 1.8 × 10^−3^ Pa).

#### Polarized light microscopy

The petrographic thin sections of the vertebral centra were observed in different standard research-grade polarized light microscopes, including a Zeiss Axiomat and a Leica DMLP. Observation took place in normal transmitted light, cross-polarized transmitted light, and with the addition of a half wavelength retardation plate (lambda filter) to the latter. Photomicrographs were collected with different digital cameras mounted on the microscopes.

#### Incident light microscopy

We used both analog and digital dark-field incident light microscopy on the samples before SEM observation to locate regions of interest at magnifications between 5x and 700x (e.g., Figures 4B and 5H). Samples were prepared as polished sections and covered with nail polish. Analog incident light microscopy was performed with a vintage Leitz microscope equipped with Ultropak objectives and a modern LED light source. Digital incident light microscopy was performed with a Keyence VHX 6000 system. Images were produced with the Keyence microscope in reflection mode by image stitching. Dark-field incident light microscopy will image structures from the sample surface to a few tens of micrometers below the plane of section if the material is translucent, which is the case with fossil bone. In particular, unmineralized parts of the tissue, or voids resulting from fossilization, appear light in color whereas the fossil bone matrix is dark brown. Light yellow color characterizes osteocyte lacunae as well as Sharpey fibers, at least part of which were not mineralized in life (Figures 4A and 4H).

### Quantification and statistical analysis

No quantification and statistical analysis was performed in this study. SEM images in Figures 3G, 3I, S6B, S8B, S9B, S10B, S12B, S13A, S15B, and S20B were analyzed and colorized using the ImageJ plugin, OrientationJ.^61^

## Supplemental information

Document S1. Figures S1–S52, Tables S1–S4, and Data S1

Figure360. An author presentation of Figure 3
