# Supplementary material for: Medusa’s gaze: Cell traces and fibrils but no collagen in permineralized Jurassic ichthyosaur bone
Source: iScience. 2024 Dec 3;28(1):111523. doi: 10.1016/j.isci.2024.111523 (PMC11732707; doi:10.1016/j.isci.2024.111523)
Supplement: Document S1. Figures S1–S52, Tables S1–S4, and Data S1 [file mmc1.pdf]

iScience, Volume *28*

Supplemental information

Medusa's gaze: Cell traces and fibrils but no collagen in permineralized Jurassic ichthyosaur bone

René-Paul Eustache, Alan Boyde, Xavier Jaurand, and P. Martin Sander

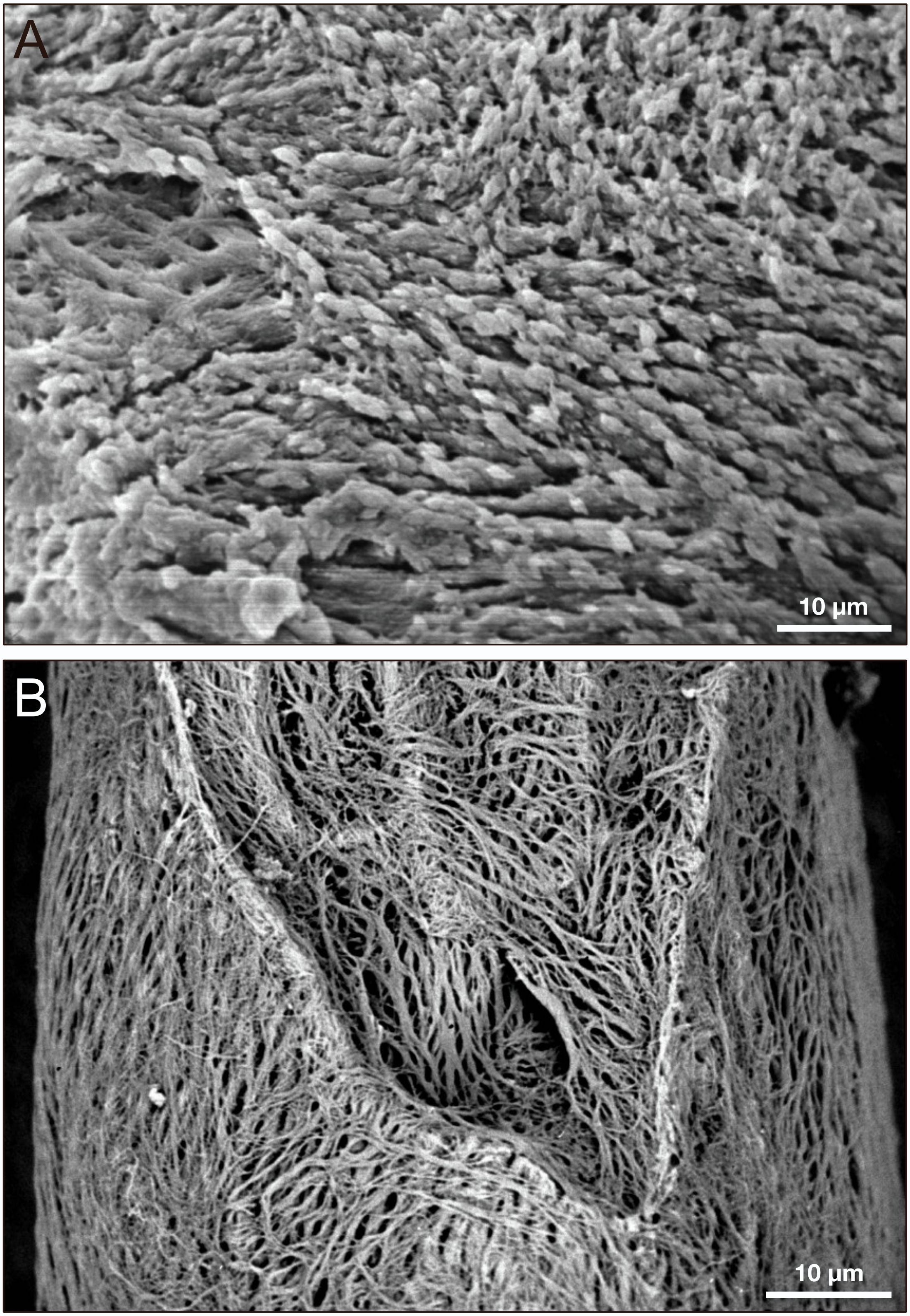

Figure S1. Modern bone, related to Figure 1 and 2
(A) Forming surface in mouse bone made anorganic with NaOCl: mineralizing front with mineralization complete in the back wall of the forming osteocyte lacuna (left). Note the characteristic (lozenge) shape of the mineralized portions of the collagen fiber bundles.
(B) Bone fibril bundles on the surface of a trabecula in human bone cleaned with Water Pik. Both samples were Au coated and imaged at 10kV at high vacuum using an SE detector.
Scale bars in A, B equal 10 µm.

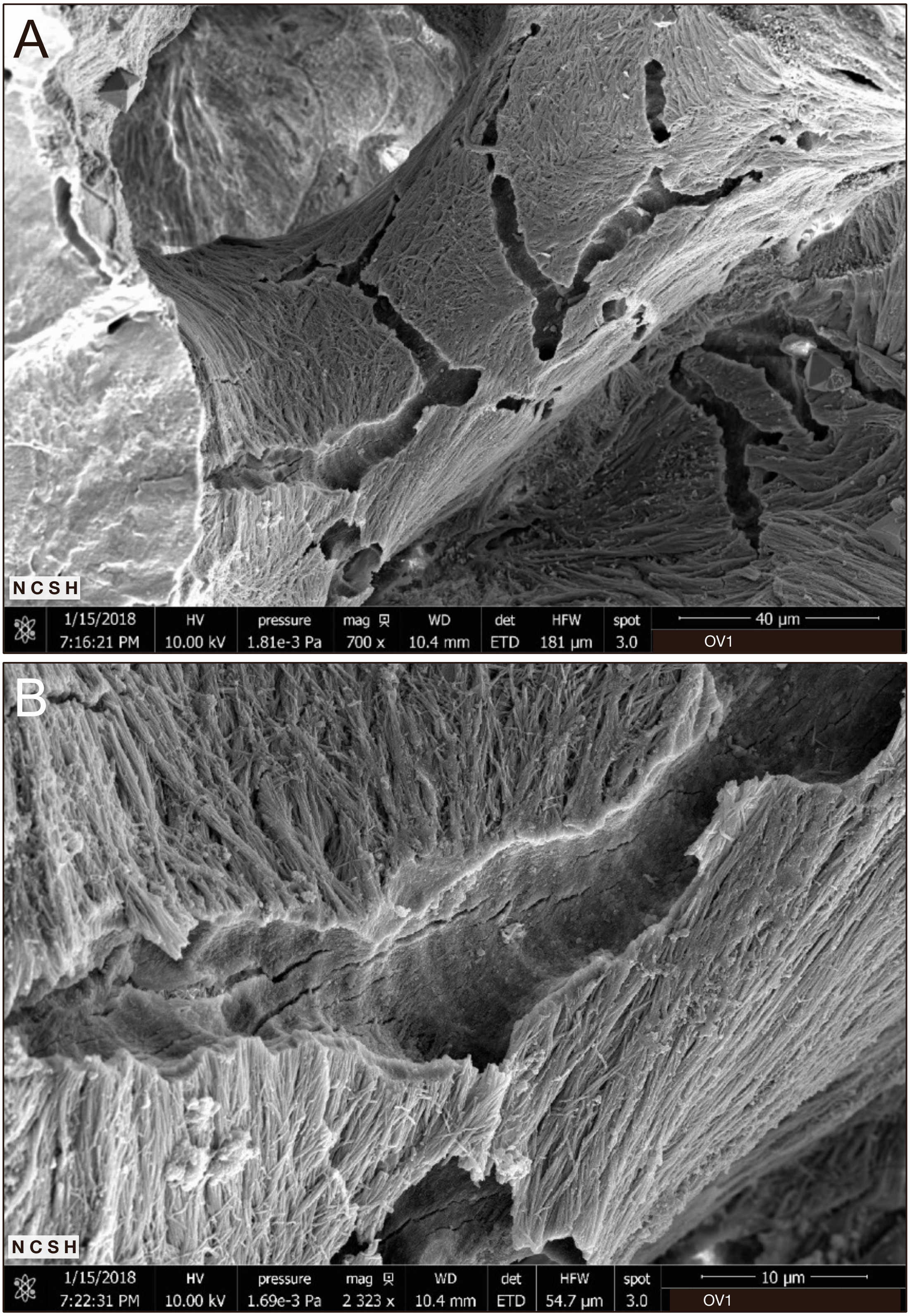

Figure S2. Trabecular bone attacked by microbial borings, OV2, related to Figure 1
(A) Tunnels at the interface between a trabecular resting surface and the bone marrow.
(B) Close-up of a tunnel. Note the annulated nature of the tunnel. SEM image code in lower left, valid for this and all the following figures: acid treatment: N/A/P, No treatment/Acetic acid treatment/Phosphoric acid etching; coating: U/C, Uncoated/Coated with Au/PD; SEM detector type: S/B, Secondary electron/Backscattered electron; vacuum: H/L, High vacuum (e.g., $1.8x10^{-3}$ Pa) / Low vacuum (e.g., 80 Pa).
Scale bar in A equals 40 µm; scale bar in B equals 10 µm.

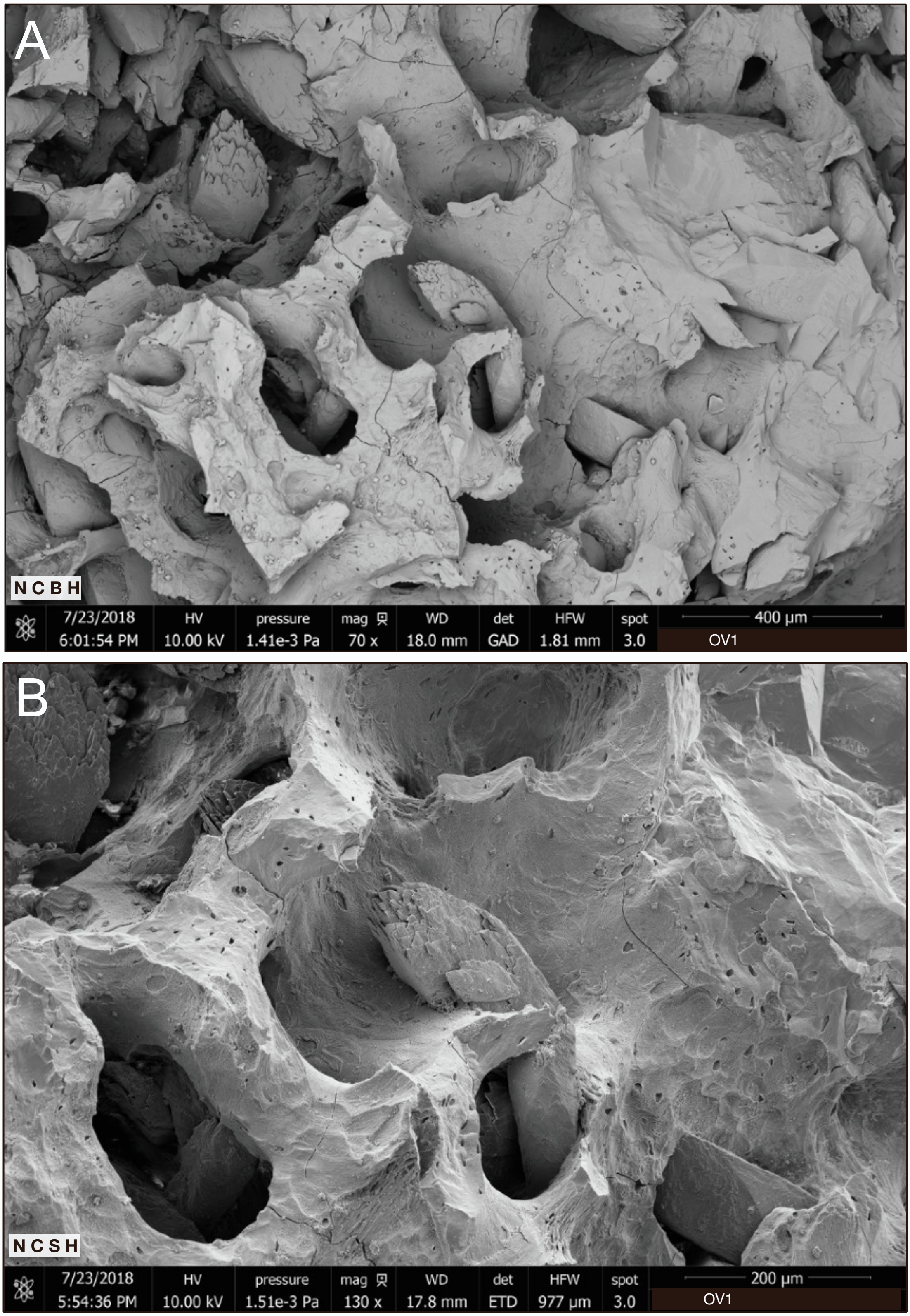

Figure S3. Idiomorphic calcite crystals which have grown into open trabecular spaces, OV1, related to Figures 2 and 6

(A) Overview showing several large idiomorphic crystals. Their continued growth would have resulted in complete closure of the primary porosity. Such crystals document the primary nature of the porosity, preserved from the Jurassic.

(B) Close-up of central area in (A). Calcite crystal grew on a bone resting surface. Other types of surfaces, such as resorption surfaces, are also visible, as are the open osteocyte lacunae.

Scale bar in A equals 400 µm; scale bar in B equals 200 µm.

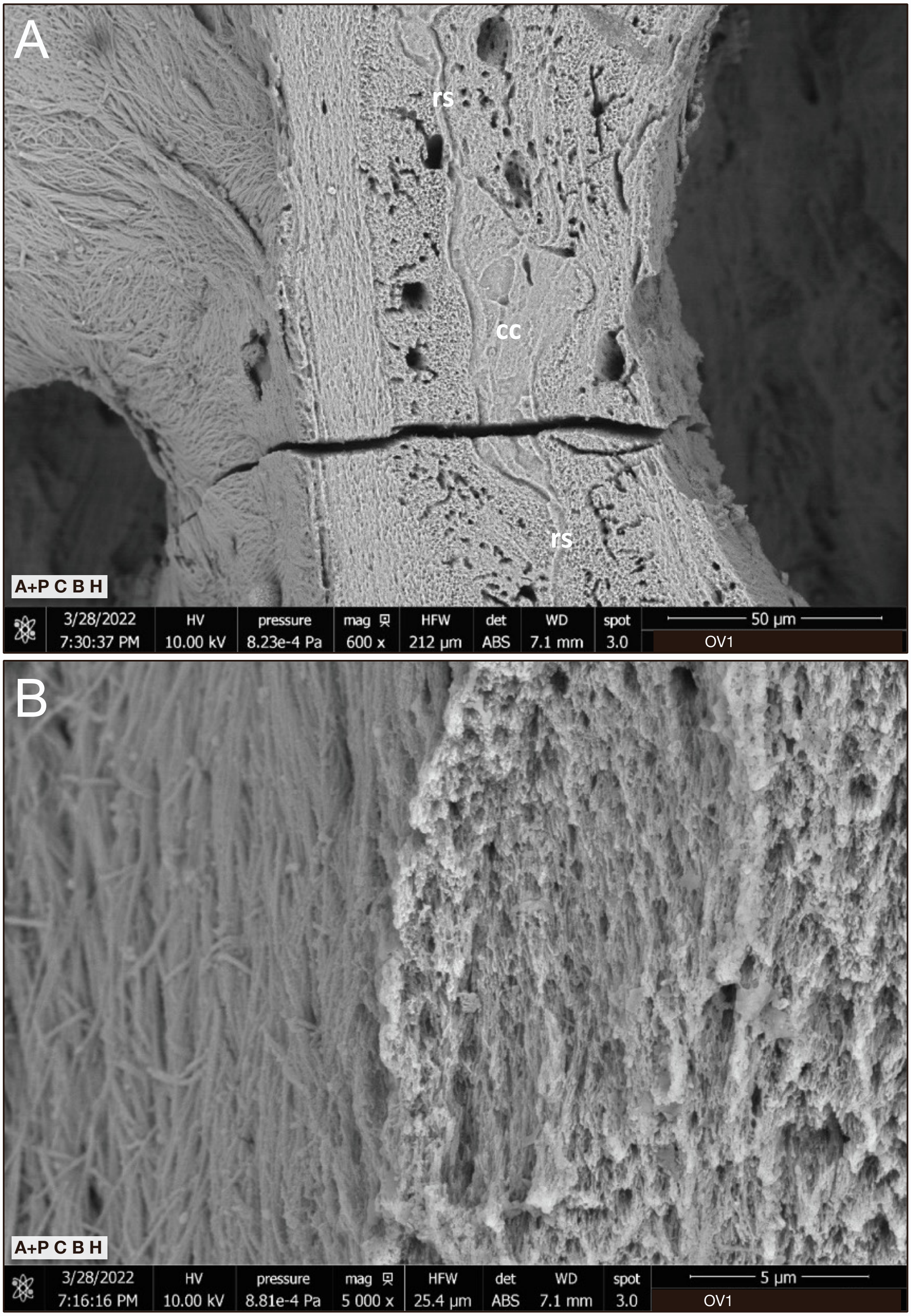

Figure S4. Phosphoric acid etching reveals bone fibrils in section planes, OV1, related to STAR Methods
(A) Etched section of endochondral trabecula with different fiber bundle orientation typical of lamellar bone. Smooth area in center is a residual calcified cartilage island. Fibers on surface have a larger diameter than sectioned fibers. Etching brings out reversal lines.
(B) Difference of fiber diameter between trabecular surface fibers (left) and sectioned etched fibers (right). Abbreviations: cc, calcified cartilage matrix; rs, resorption line.
Scale bar in A equals 50 µm; scale bar in B equals 5 µm.

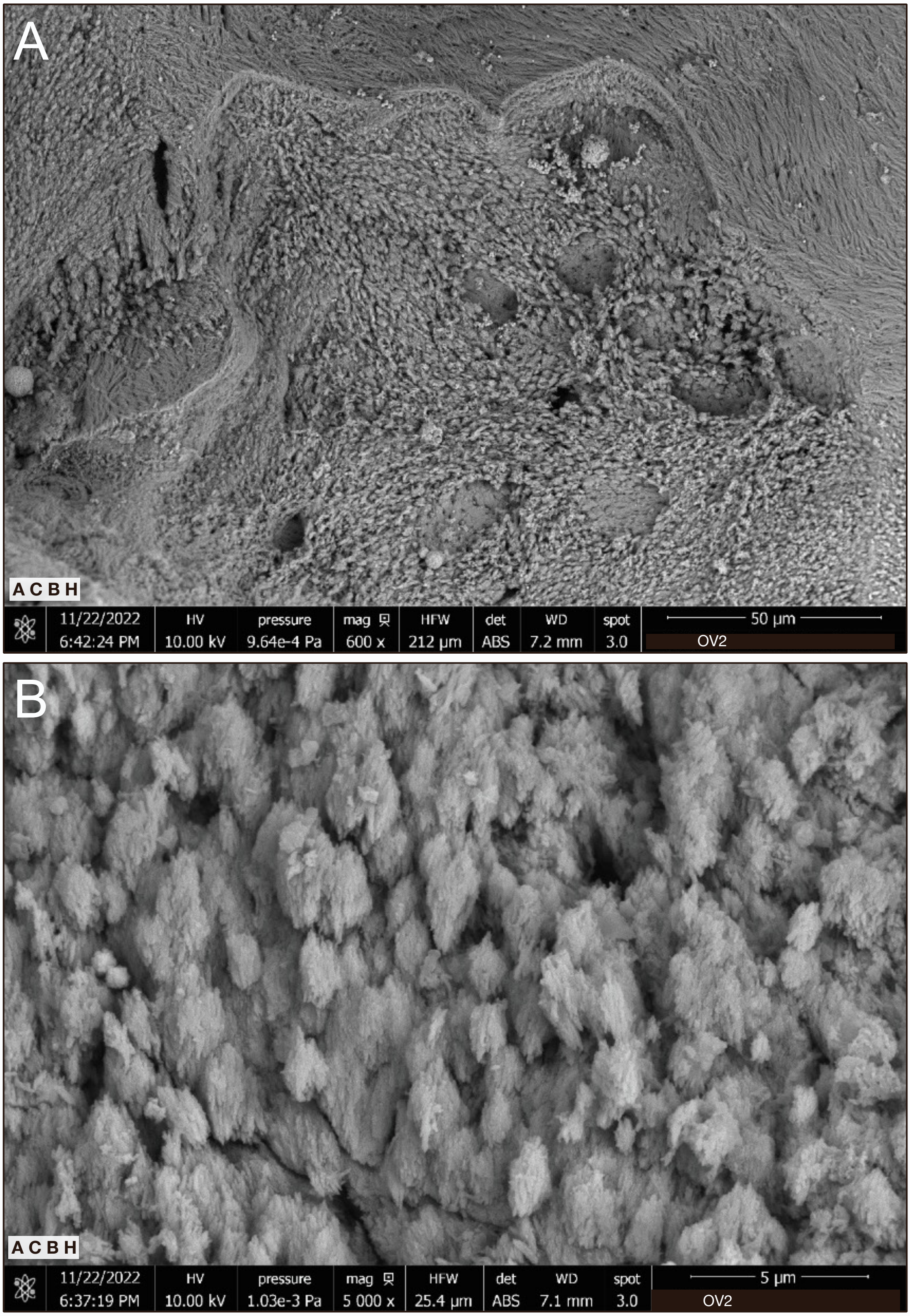

Figure S5. Forming surfaces, OV2, related to Figure 3
(A) Forming surface developing over a resorbed surface, the classical "coupled" situation. Mineralization of the fiber bundles, retaining as fossils only those parts that were already mineralized in the living animal.
(B) Close-up, note the characteristic (lozenge) shapes of the mineralized portions of the collagen fiber bundles typical for forming surfaces.
Scale bar in A equals 50 µm; scale bar in B equals 5 µm.

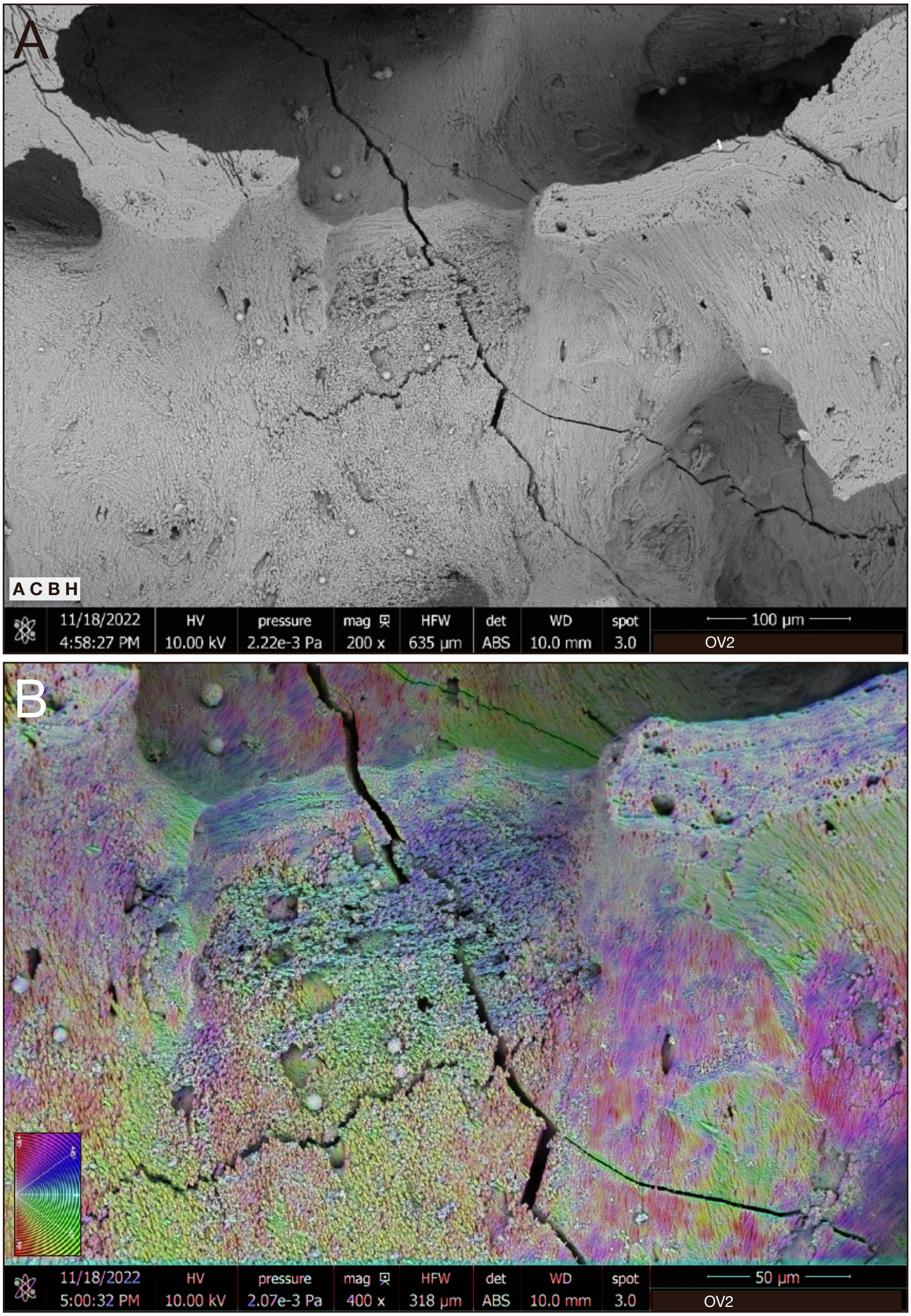

Figure S6. Forming surfaces, OV2, related to Figure 3
(A) Domains, areas with a common fiber orientation on both resting and forming surfaces. Forming surface is filling in a resorption cavity.
(B) False-color image of close-up, indicating fiber directions created by OrientationJ.
Scale bar in A equals 100 µm; scale bar in B equals 50 µm.

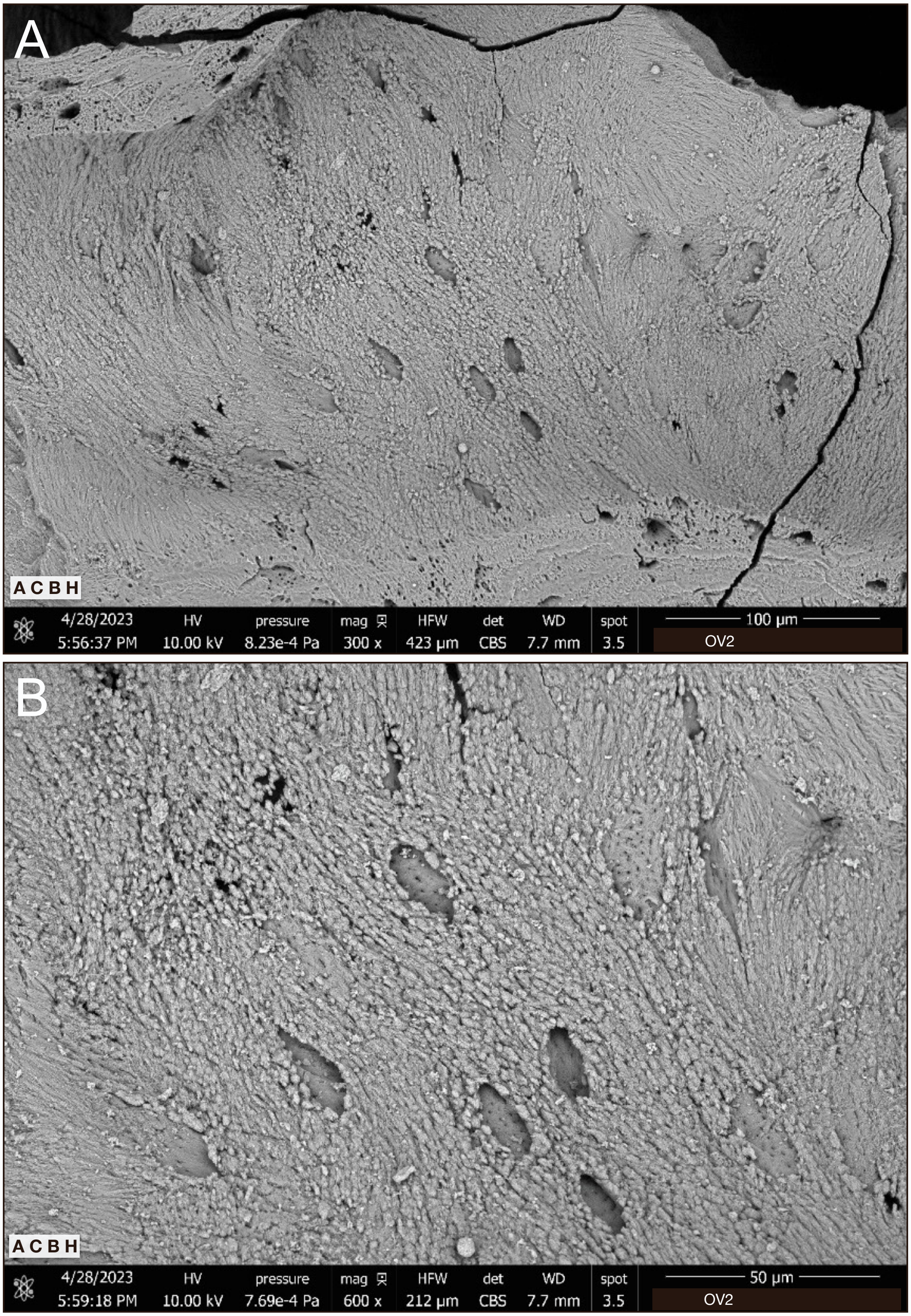

Figure S7. Forming surface covering resting surface, OV2, related to Figures 3 and 4
(A) Forming surfaces are not only seen over resorption surfaces but also on resting surfaces, indicating renewed osteoblast activity.
(B) Close-up, note the open osteocyte lacunae, recording the incorporation of these cells into the bone matrix. Mineralization is complete in the back walls of the lacunae.
Scale bar in A equals 100 µm; scale bar in B equals 50 µm.

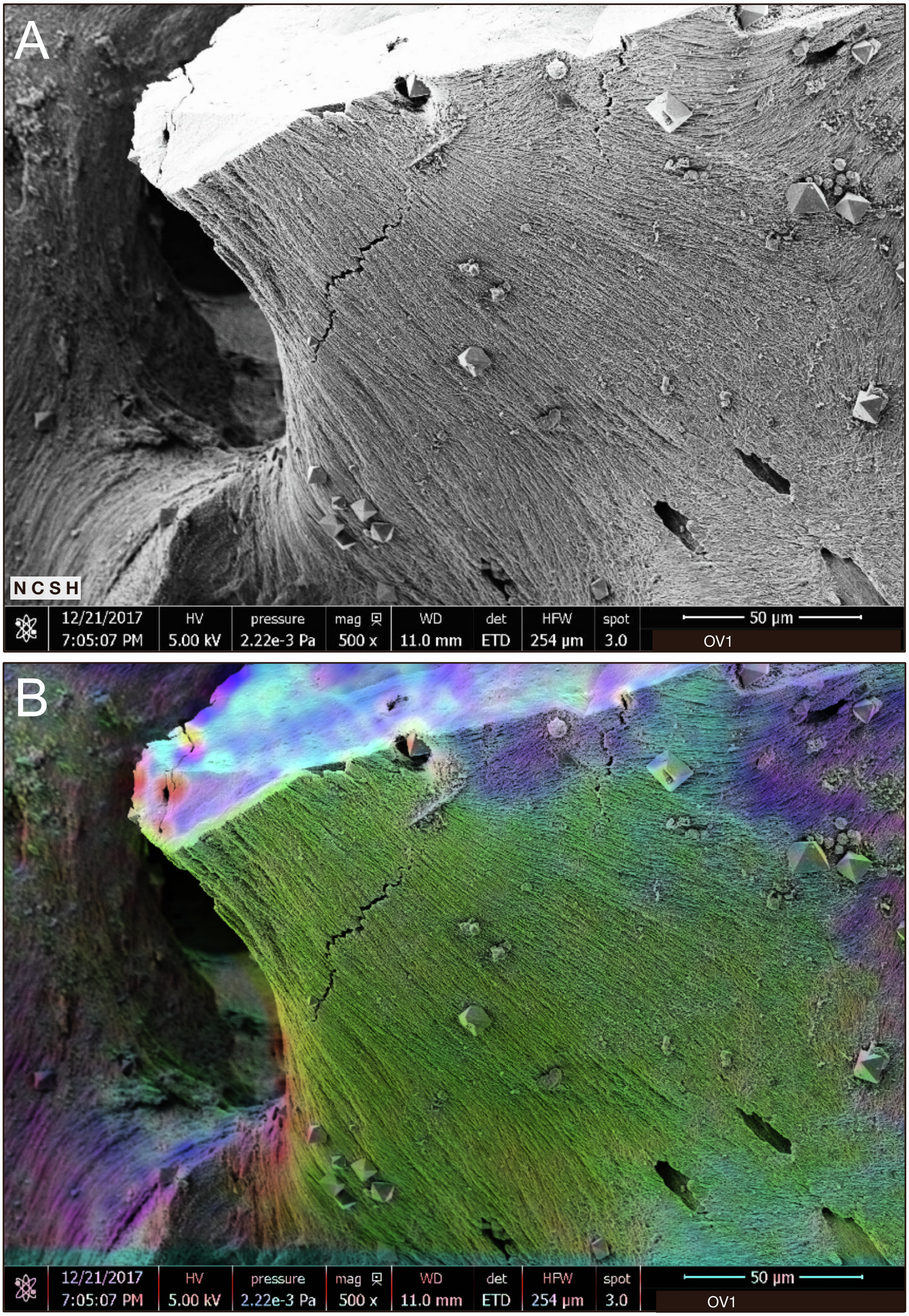

Figure S8. Resting surfaces with mostly parallel fiber bundles, OV1, related to Figure 3
(A) Domain of ordered parallel fibrils. Note the pyrite octahedra that grew on the bone resting surface after the death of the animal. Also, the fracture surfaces reveal no internal structure. This is a higher resolution version of image in main text Figure 3.
(B) False-color image of (A), emphasizing the parallel fibers.
Scale bars in A, B equal 50 µm.

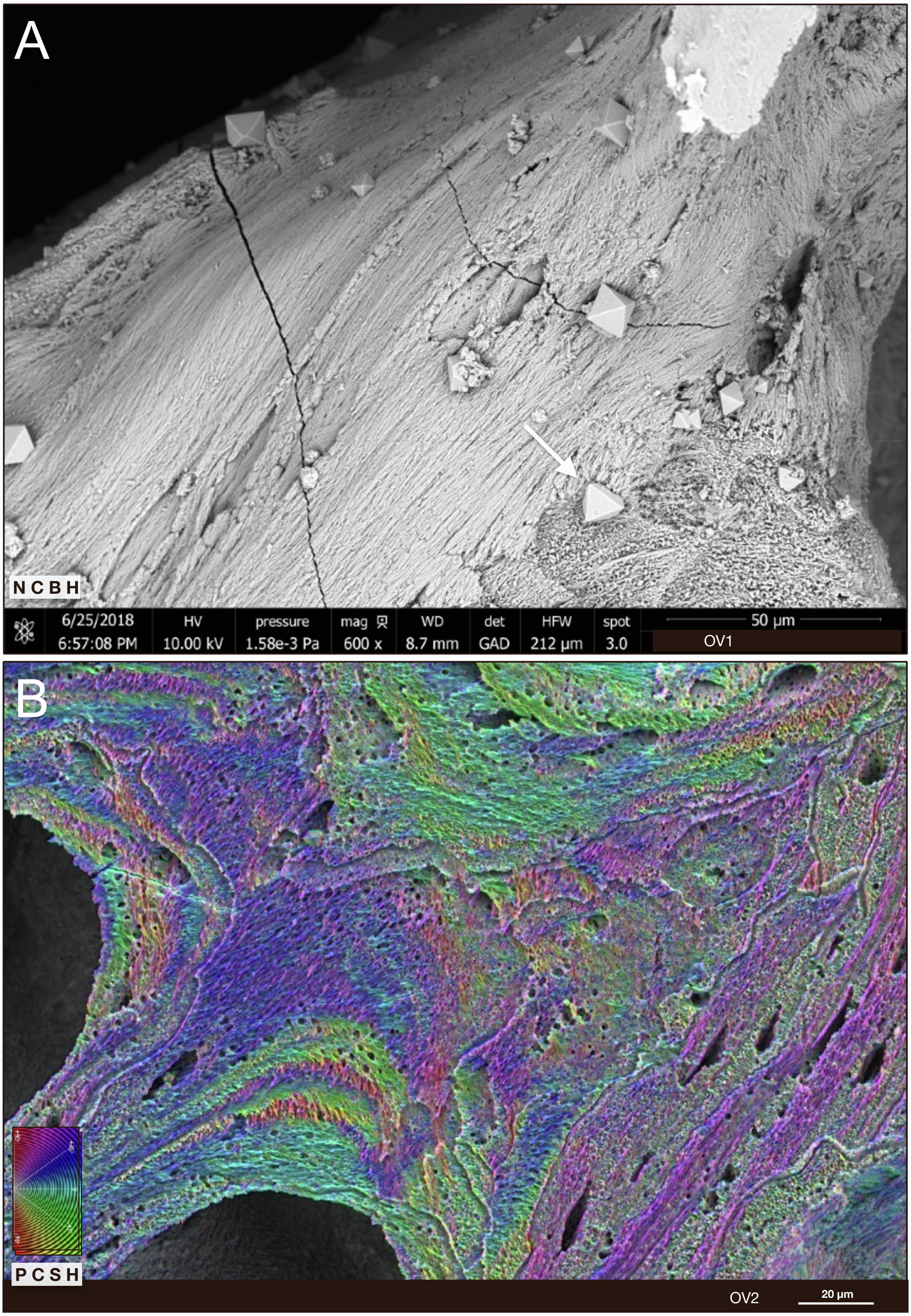

Figure S9. Resting surfaces with mostly parallel fibers, related to Figure 3
(A) OV1, domain of parallel fibers. Note the pyrite octahedron that grew on the boundary between the domain and a resorption surface (arrow). (B) OV2, phosphoric acid etched section of trabecula exposing layers of bone fibers with differing orientations (lamellae) as revealed by false color image. This is a wider field of view than in panel (G) of Figure 3 of main text. Scale bar in A equals 50 µm; scale bar in B equals 20 µm.

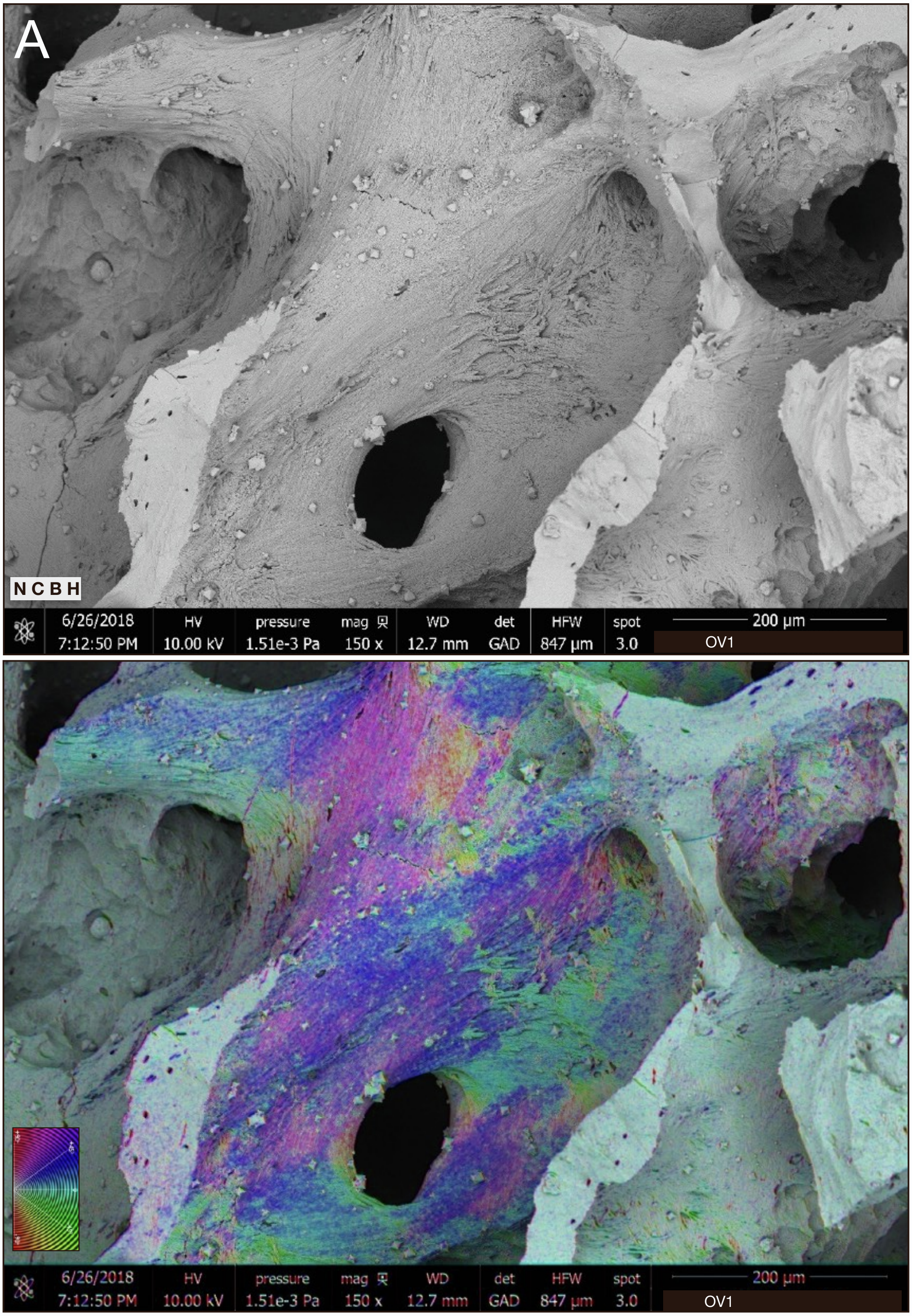

Figure S10. Resting surfaces with domains of various orientation, OV1, related to Figure 3
(A) Different domains of largely parallel fibers cover the internal trabecular surfaces.
(B) False-color image indicating fiber directions created by OrientationJ.
Scale bars in A, B equal 200 µm.

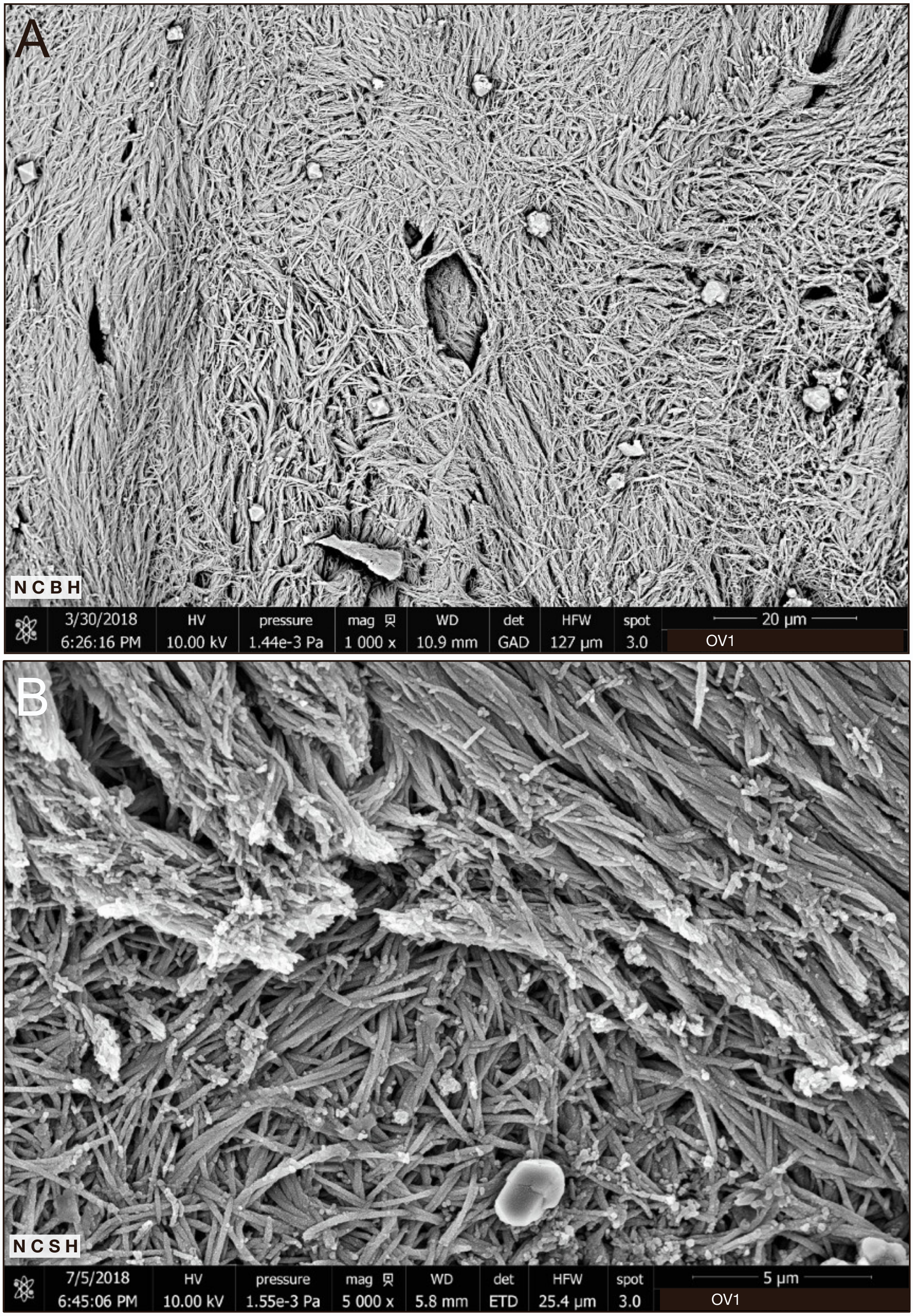

Figure S11. Resting surface with 2D non-parallel fibers, OV1, related to Figure 3

(A) Low magnification, note the osteocyte lacuna in the center of image. Also note that the non-parallel fibers are sometimes curved.

(B) Interface between a domain of non-parallel fibers and an overlying domain of parallel fiber bundles.

Scale bar in A equals 20 μm; scale bar in B equals 5 μm.

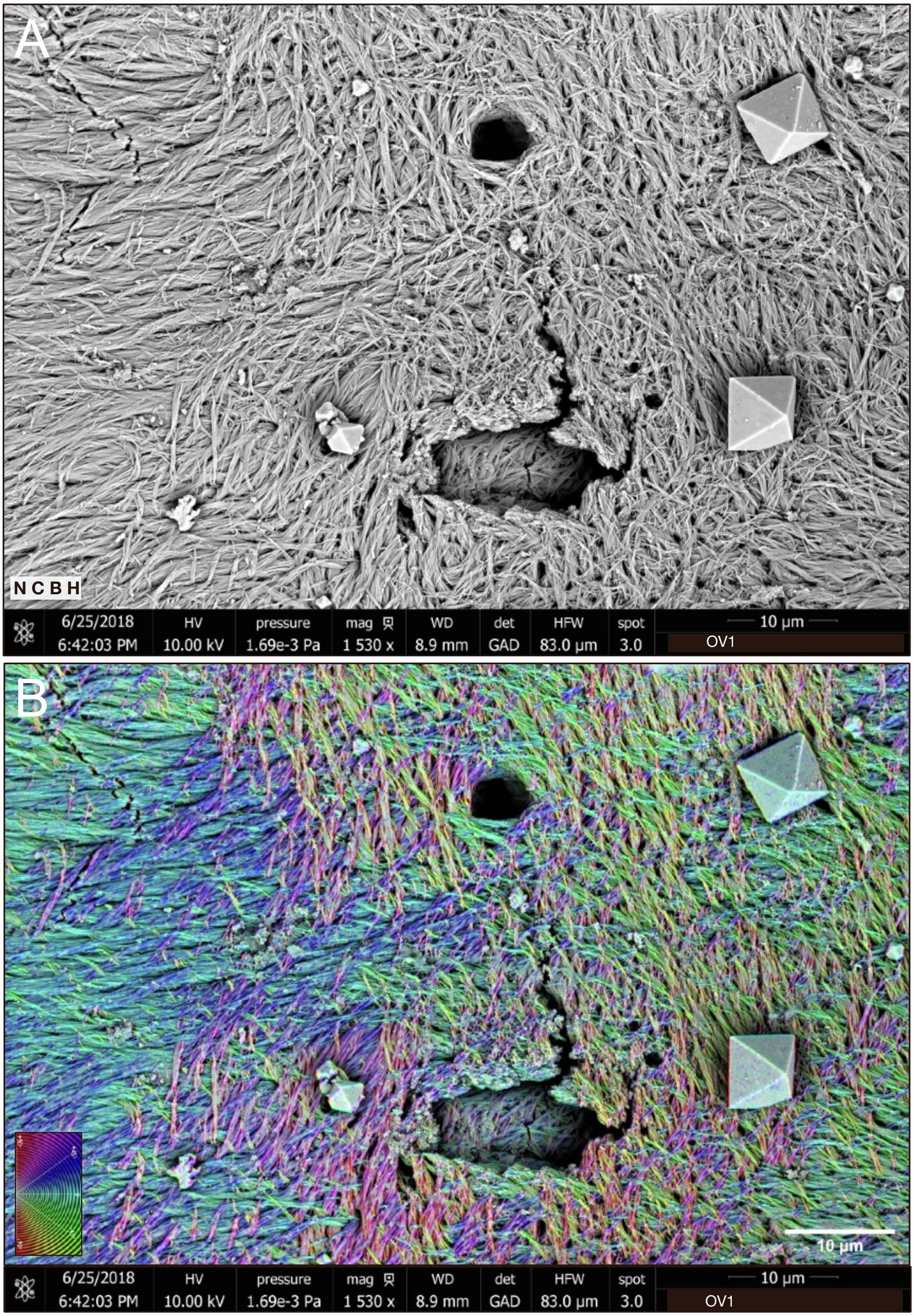

Figure S12. Resting surface with 2D non-parallel fibers, OV1, related to Figure 3
(A) Note the osteocyte lacuna, and the pyrite crystals. This is a higher resolution version of image in Figure 3.
(B) False-color image of non-parallel fibrils created by OrientationJ.
Scale bars in A, B equal 10 µm.

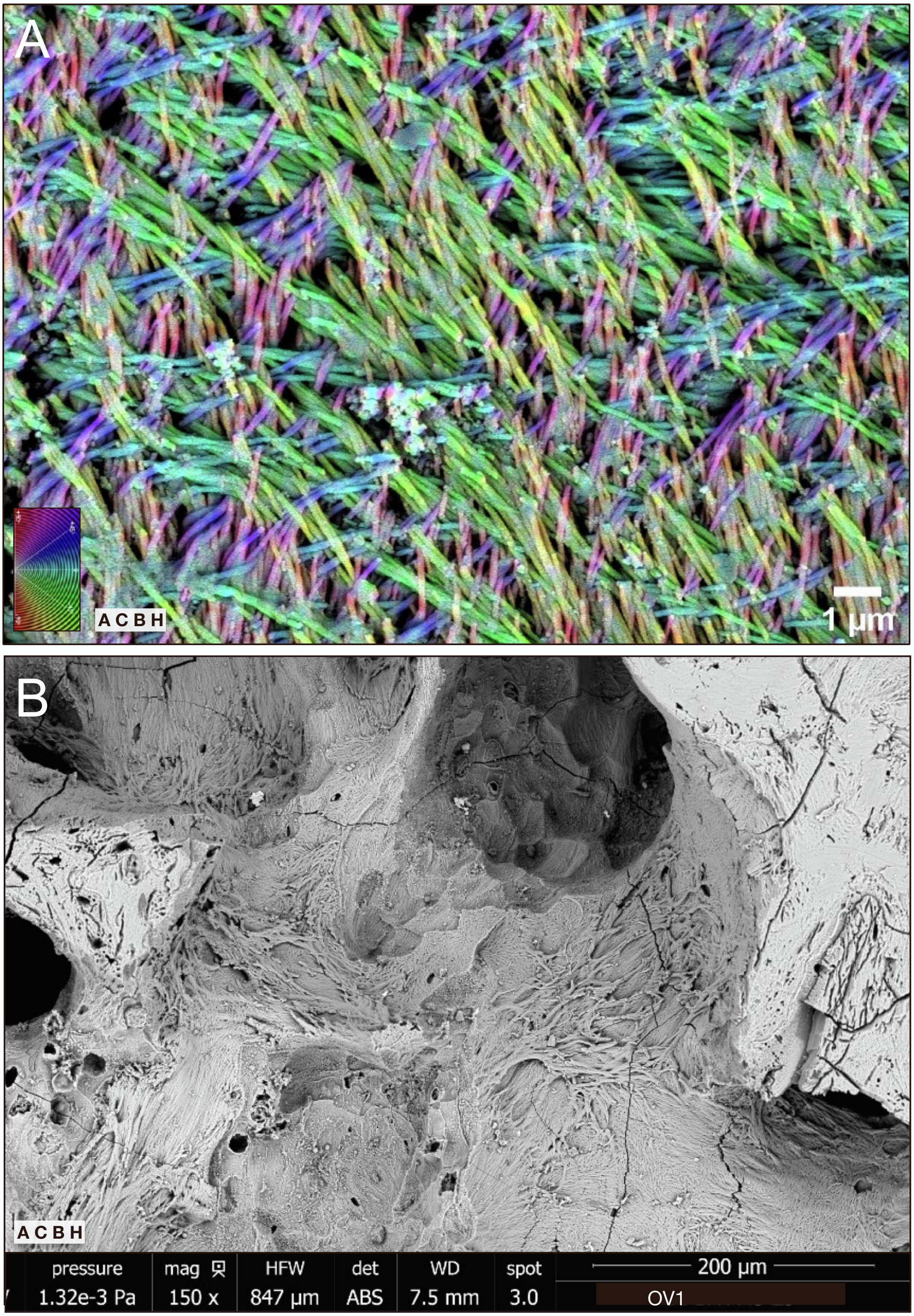

Figure S13. Resting surface with non-parallel branching fiber bundles, OV1, related to Figures 3 and 4

(A) False-color image of non-parallel fibers created by OrientationJ. This is an enlargement of Figure S12B and a higher resolution of the image in Figure 3.

(B) Complex non-parallel fiber bundles.

Scale bar in A equals 1 µm; scale bar in B equals 200 µm.

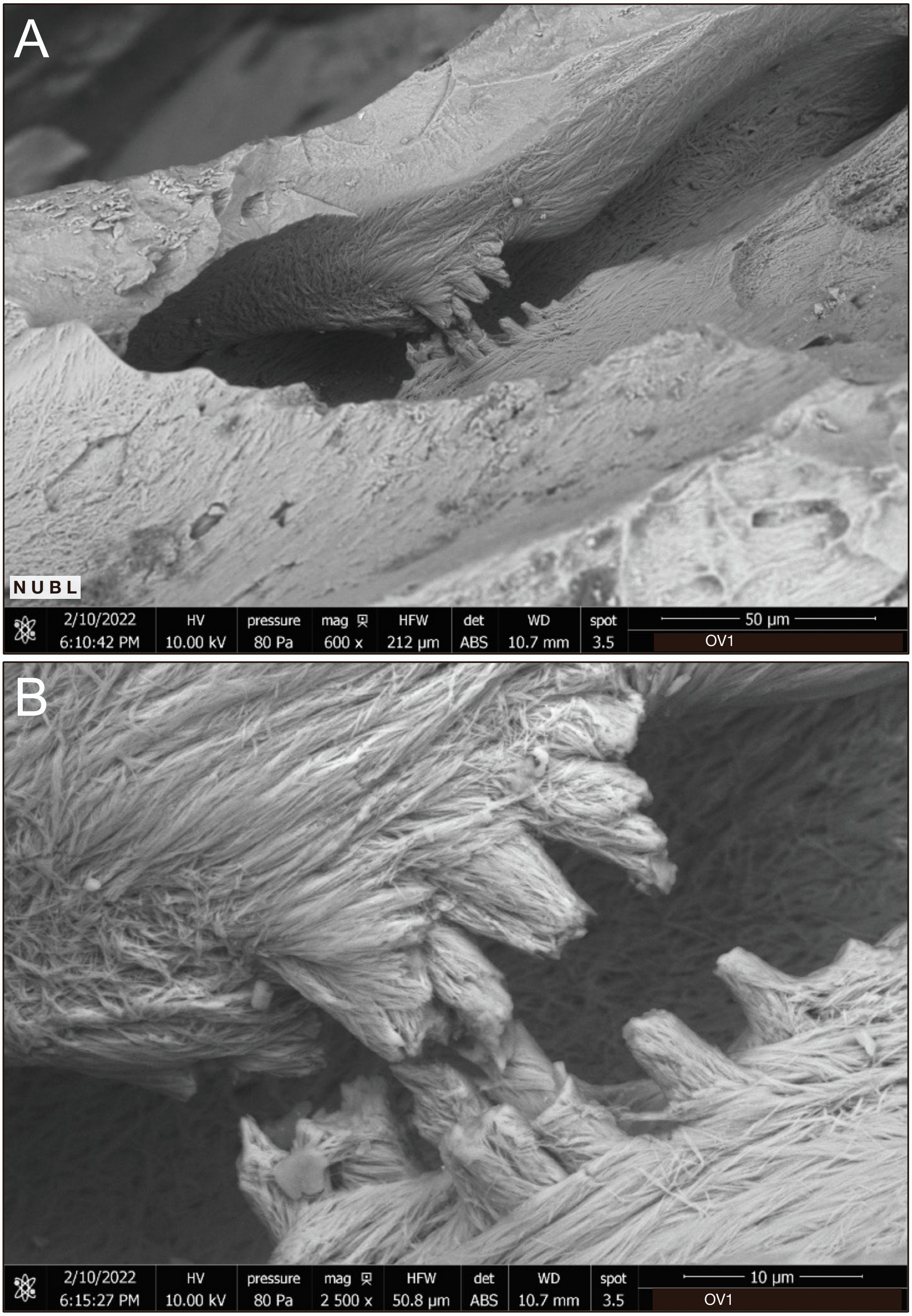

Figure S14. Partially mineralized fiber bundles connecting two trabeculae, OV, related to Figure 3

(A) Overview of trabecular bone in the endochondral territory.
(B) Close-up of bundles.
Scale bar in A equals 50 μm; scale bar in B equals 10 μm.

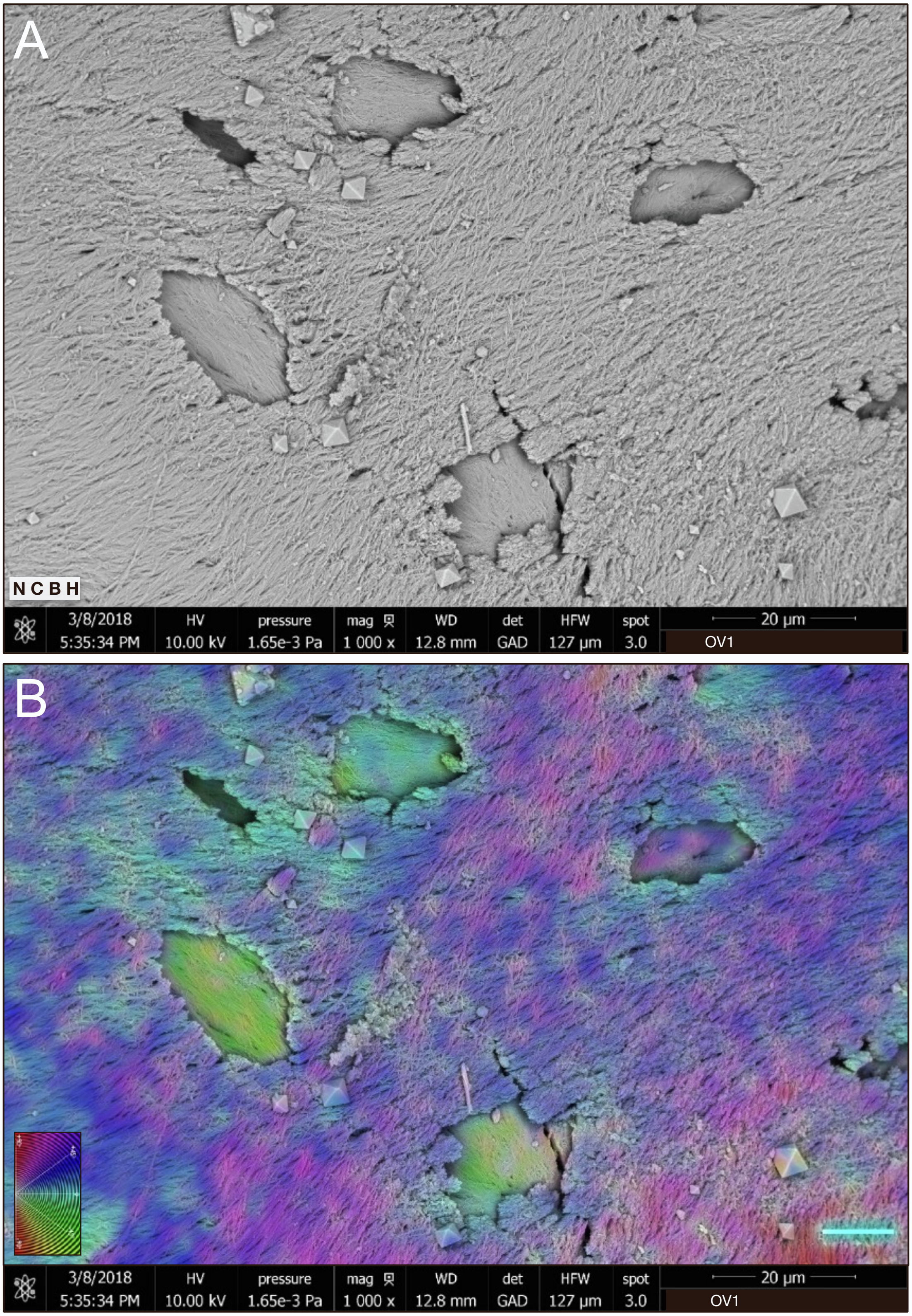

Figure S15. Resting surface with osteocyte lacunae set in moderately parallel fiber bundles, OV1, related to Figure 4
(A) Regular SEM image.
(B) False-color image indicating fiber directions created by OrientationJ. Note the higher degree of fibril orientation in the back of the osteocyte lacunae and different orientation of those fibrils Scale bars in A, B equal 20 µm.

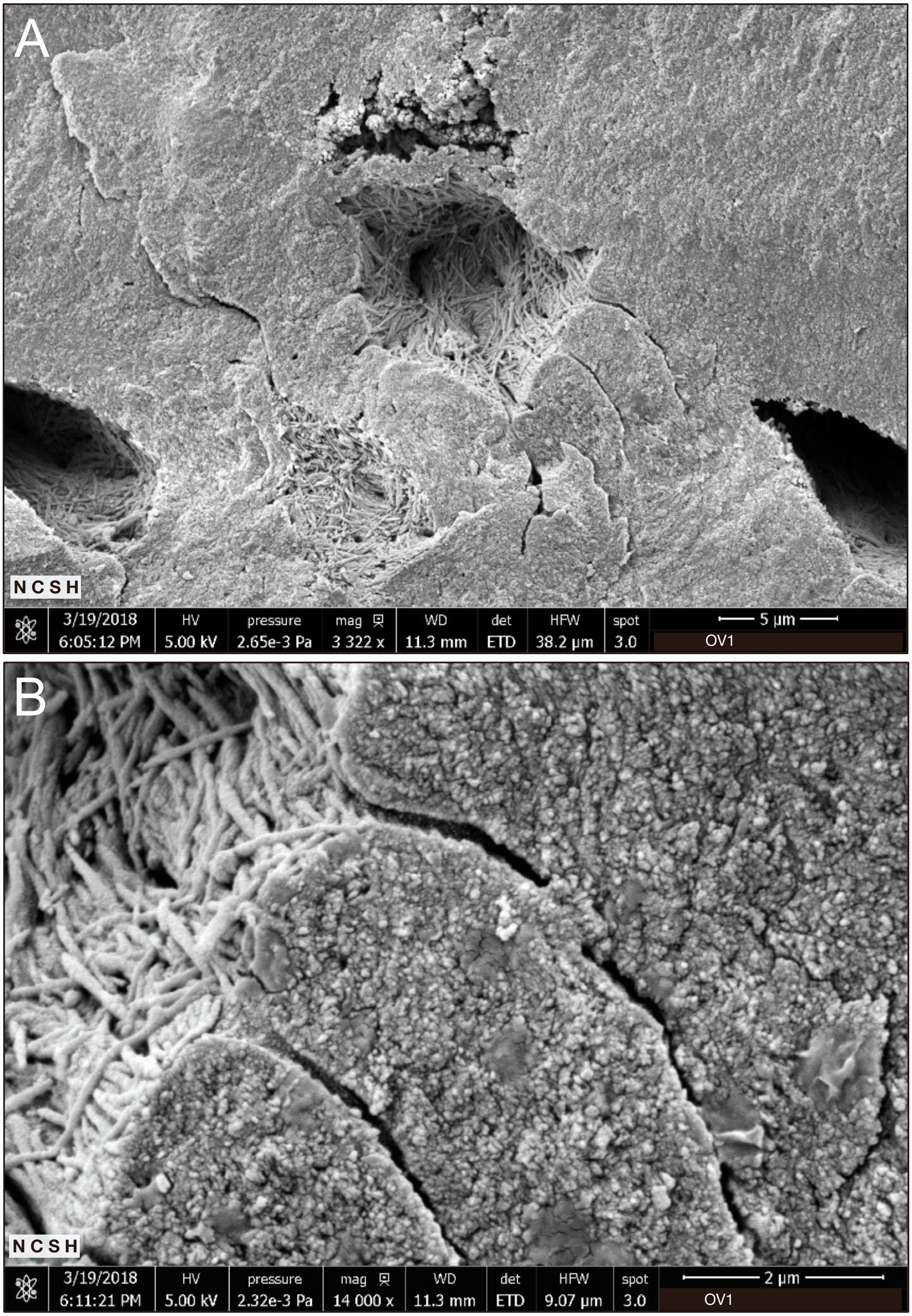

Figure S16. Open osteocyte lacunae in fracture surface, OV1, related to Figure 4
(A) Empty osteocyte lacunae revealed by a fracture surface. Note the fibrils in the lacunar wall and the homogeneous fracture surface
(B) Close-up of lacuna in center of (A). Note the empty canaliculi extending from the lacuna. Scale bar in A equals 5 µm; scale bar in B equals 2 µm.

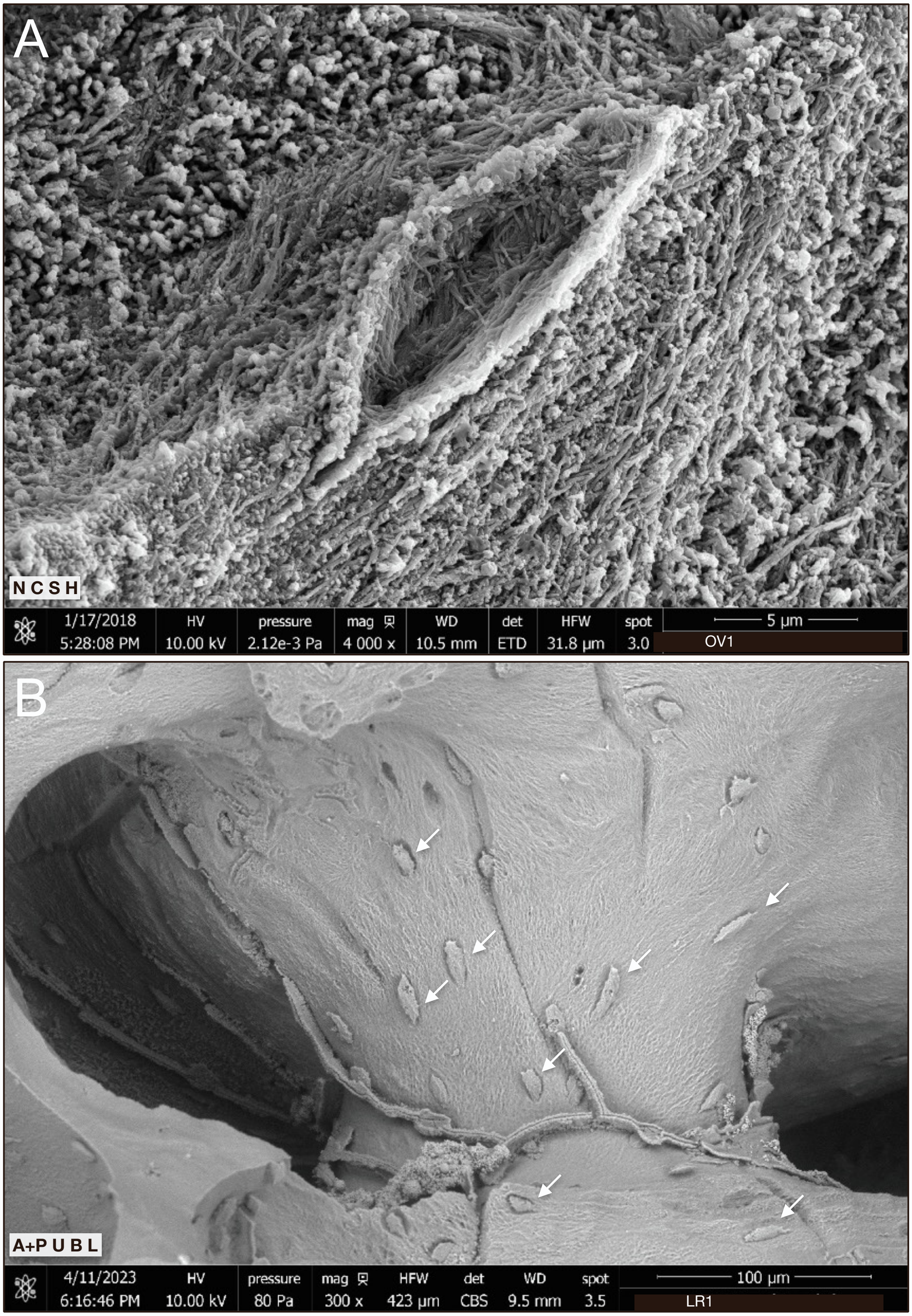

Figure S17. Osteocyte lacunae and lacunar casts, related to Figure 4
(A) OV1, osteocyte lacuna exposed by osteoclastic resorption during the animal's lifetime. The osteocyte may have been released by this process. This is a higher resolution version of image in Figure 4.
(B) LR1, spindle-shaped, flattened osteocyte lacunae (some marked by arrows) are preserved as casts. This osteocyte shape is typical for dynamic osteogenesis forming parallel-fibered and lamellar bone. The etching removed some bone fibrils, leading not only to the exposure of the osteocytes but also the diagenetic crack fills. These reveal the amount of material removed by etching. The infill of the lacunae consists of diagenetic apatite.
Scale bar in A equals 5 µm; scale bar in B equals100 µm.

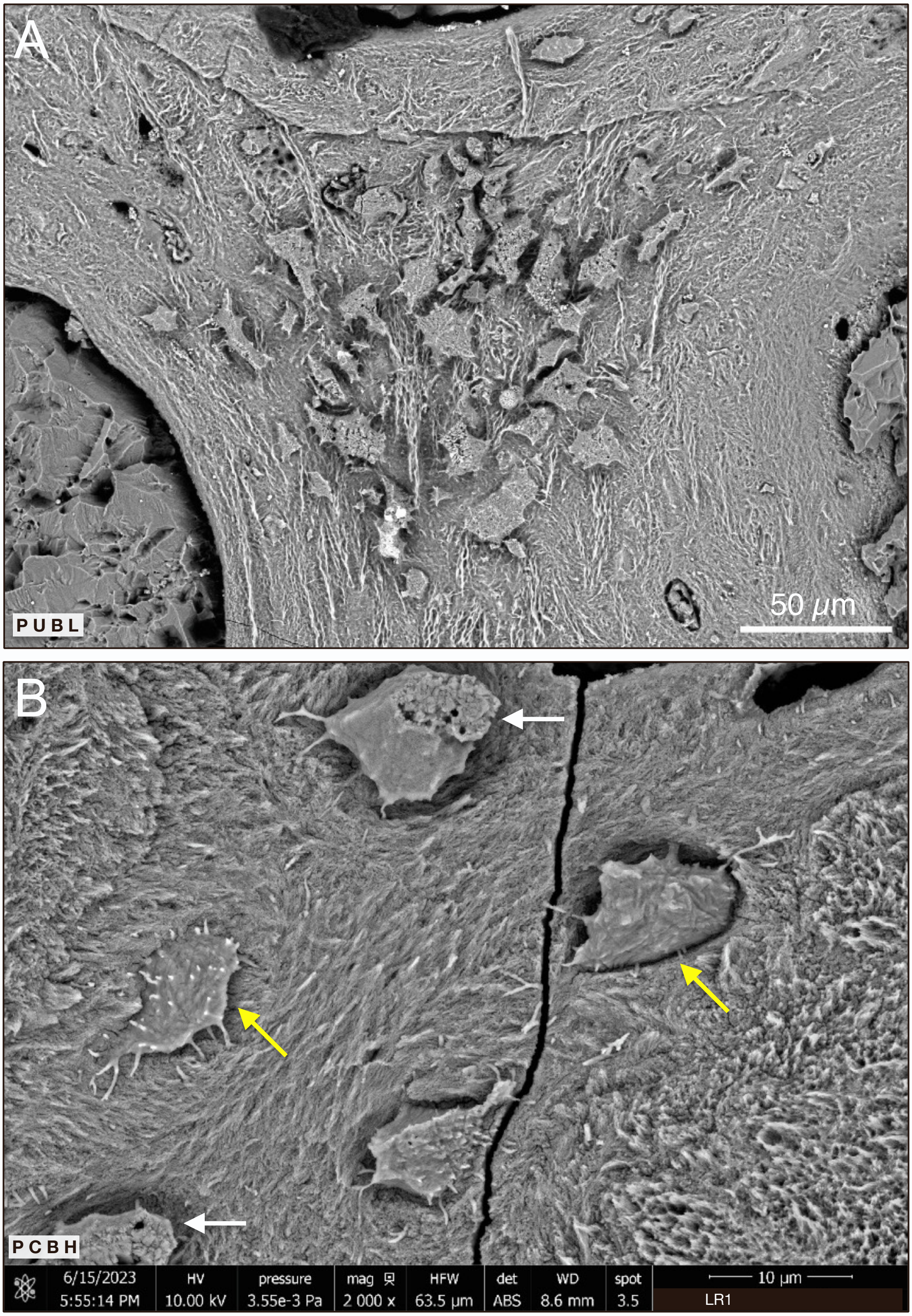

Figure S18. Osteocyte lacunar fills revealed by phosphoric acid etching, related to Figure 4
(A) OV2, cluster of irregular and densely spaced osteocyte lacunae, typical of woven bone matrix produced by static (non-scaffolded) osteogenesis. The infill of the lacunae consists of diagenetic apatite. The calcite fill of the intertrabecular spaces has also been attacked by the phosphoric acid.
(B) LR1, irregularly shaped osteocyte casts exposed by phosphoric acid etching. Note large diagenetic crystals (white arrows) inside casts and impressions of bone fibrils on cast surfaces (yellow arrows).
Scale bar in A equals 50 μm; scale bar in B equals 10 μm.

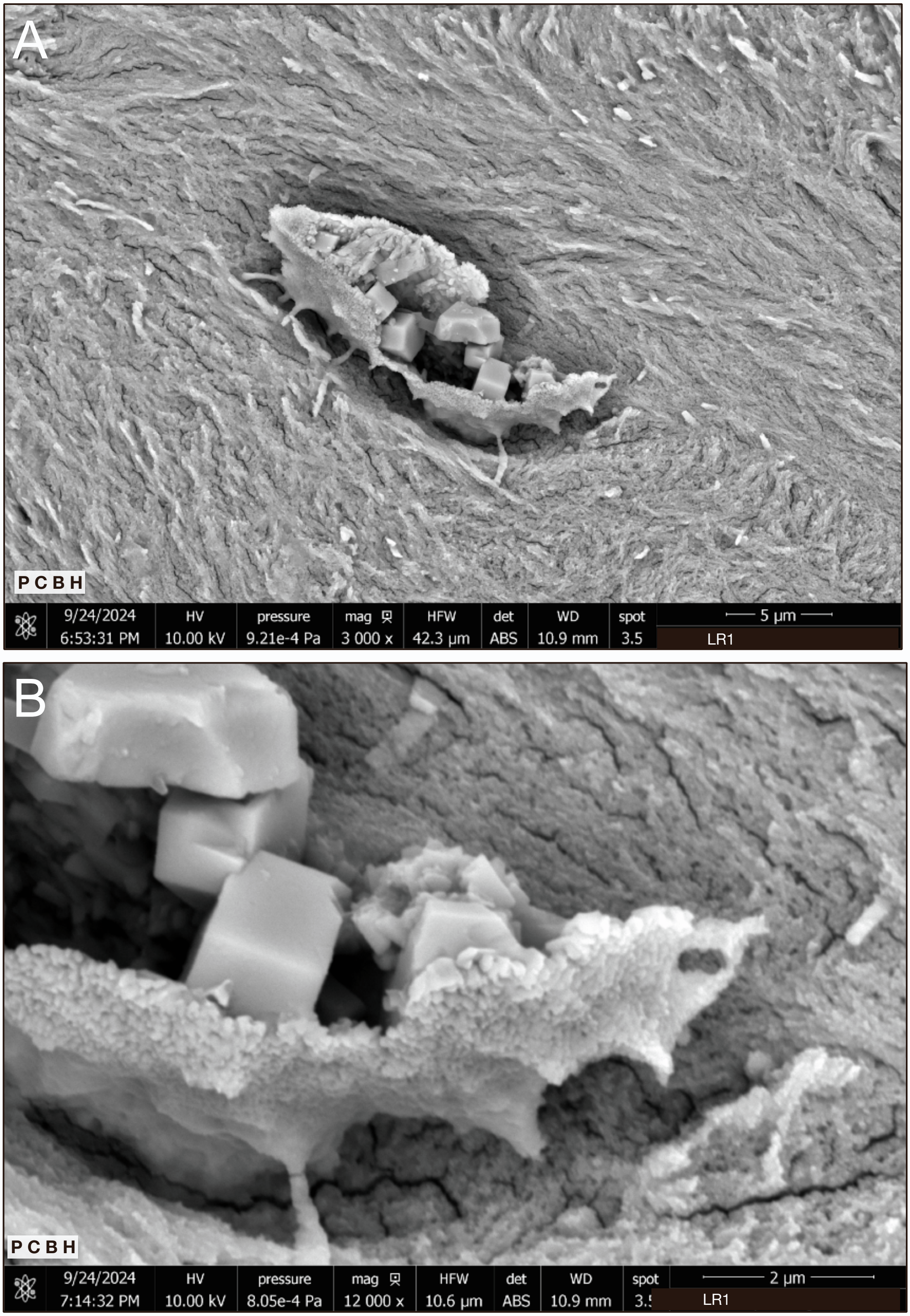

Figure S19. Single osteocyte/lacuna cast in LR1, related to Figure 4
(A) Etched surface revealing the shell and interior of cast.
(B) Close-up of above. Note the large crystals inside the cast and the fine crystals making up its shell.
Scale bar in A equals 5 μm; scale bar in B equals 2 μm.

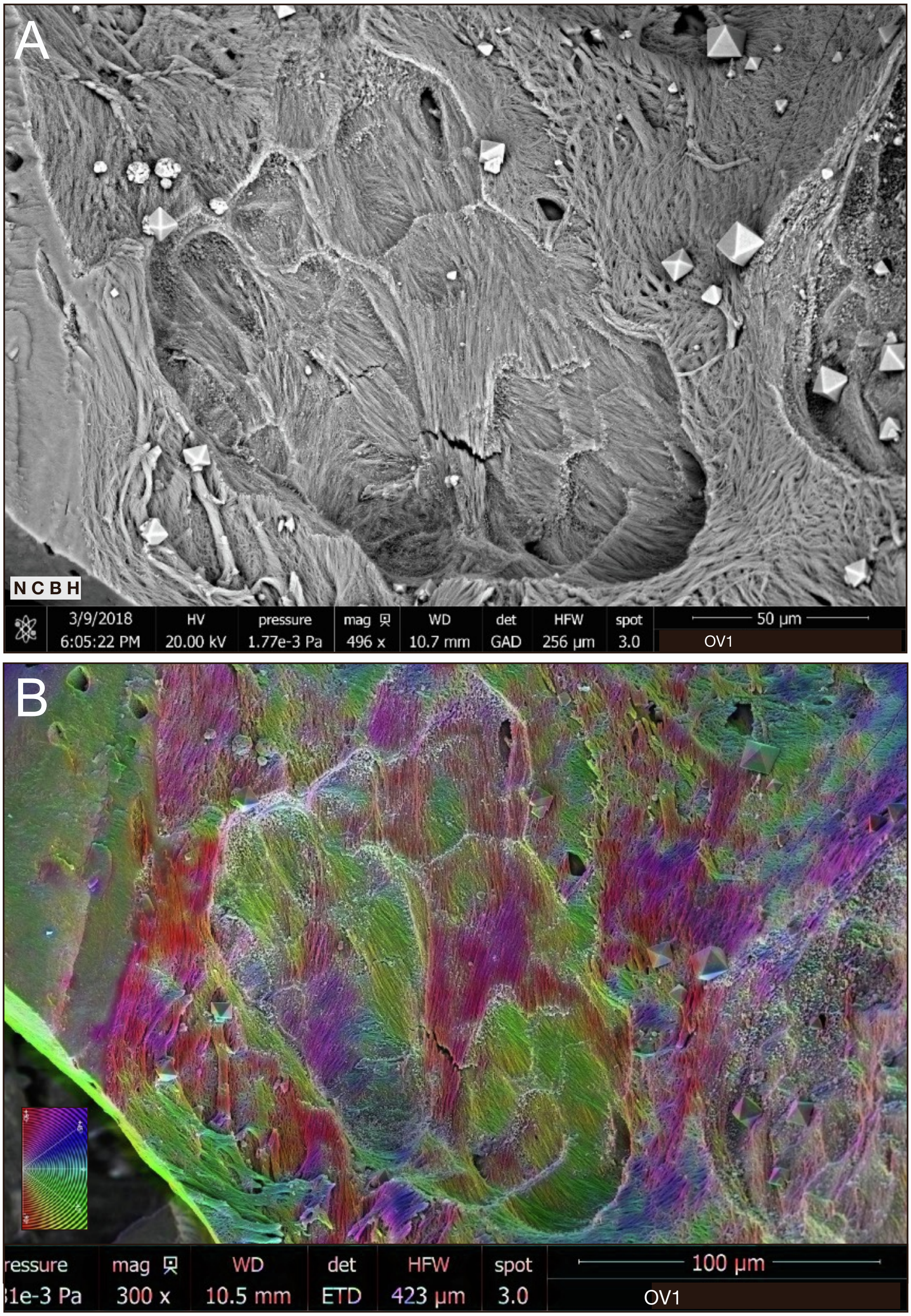

Figure S20. Resorbing surface on domains of parallel fibers, OV1, related to Figure 4
(A) Howship's lacunae resorbed into a resting surface of 2D non-parallel fibers reveal underlying domains of parallel fibers. Note the transversely cut fibers in the upper left margins of the resorption lacunae and the distinctive pyrite octahedra.
(B) False-color image of the area above, created by OrientationJ. Note the overlapping domains of parallel fibers with distinct orientation.
Scale bar in A equals 50 µm; scale bar in B equals 100 µm.

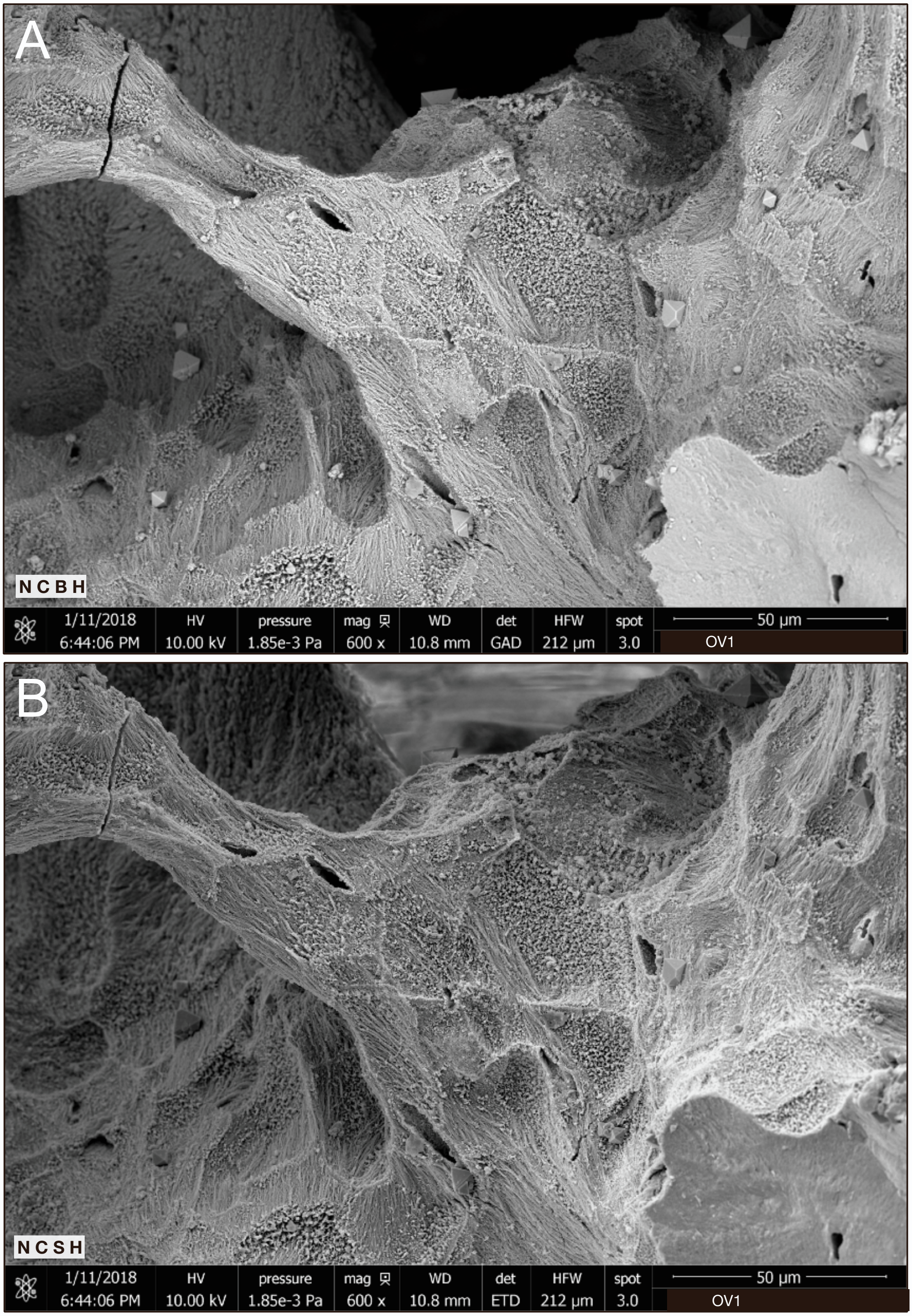

Figure S21. Resorbing surface on trabecular rod, imaged by different detectors, OV1, related to Figure 4

(A) Trabecular rod covered by resorbing surfaces, imaged with the BSE detector. Note the clearly visible pyrite crystals.
(B) Same frame of view as in (A) but with the SE detector.
Scale bars in A, B equal 50 µm.

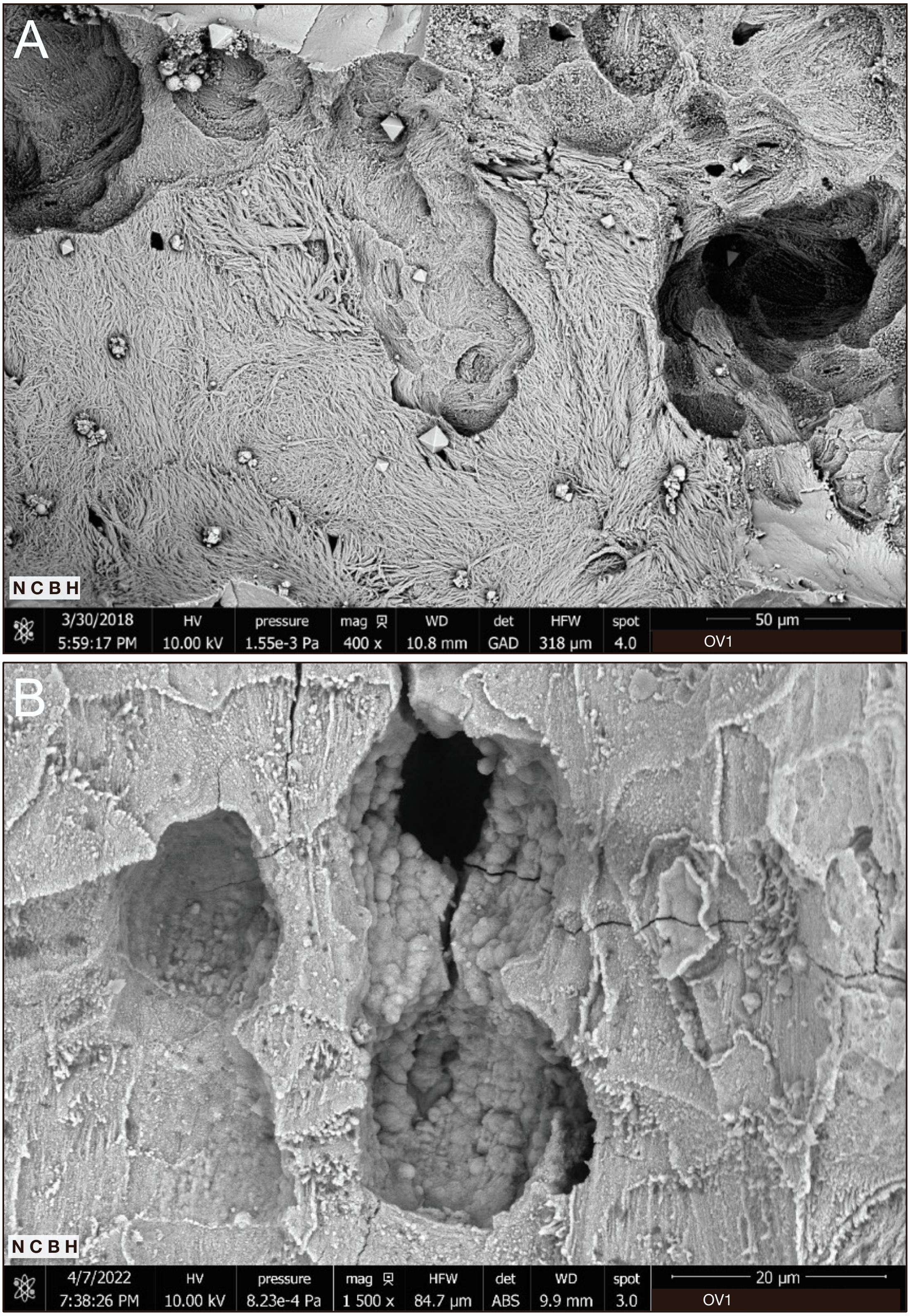

Figure S22. Resorbing surfaces on 2D non-parallel fiber domains and cartilage, OV1, related to Figures 4 and 5

(A) Howship's lacunae resorbed into a resting surface of 2D non-parallel fibers. Note the pyrite octahedra on both the resting and the resorption surfaces.

(B) Resorption surface on calcified cartilage, revealing the collagen fibrils in the cartilage and opening up chondrocyte lacunae with calcospherites on their walls.

Scale bar in A equals 50 µm; scale bar in B equals 20 µm.

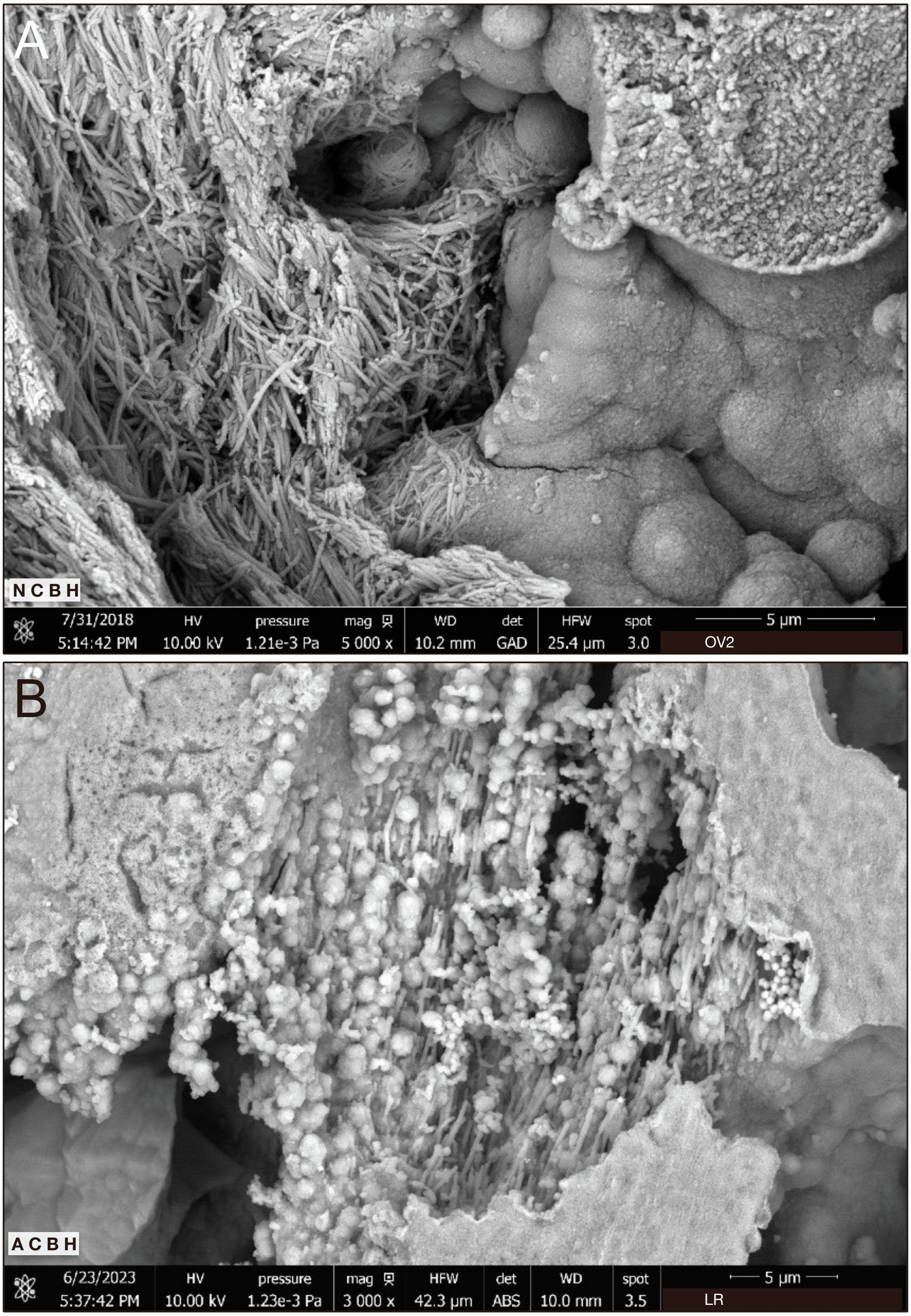

Figure S23. Cartilage and endochondral ossification, related to Figure 5
(A) OV2, area of calcospherites in cartilage on the right borders on a bone resting surface on the left.
(B) LR1, open porosity with cartilage collagen fibrils and calcospherites. Higher resolution version of image in Figure 5.
Scale bars in A, B equal 5 μm.

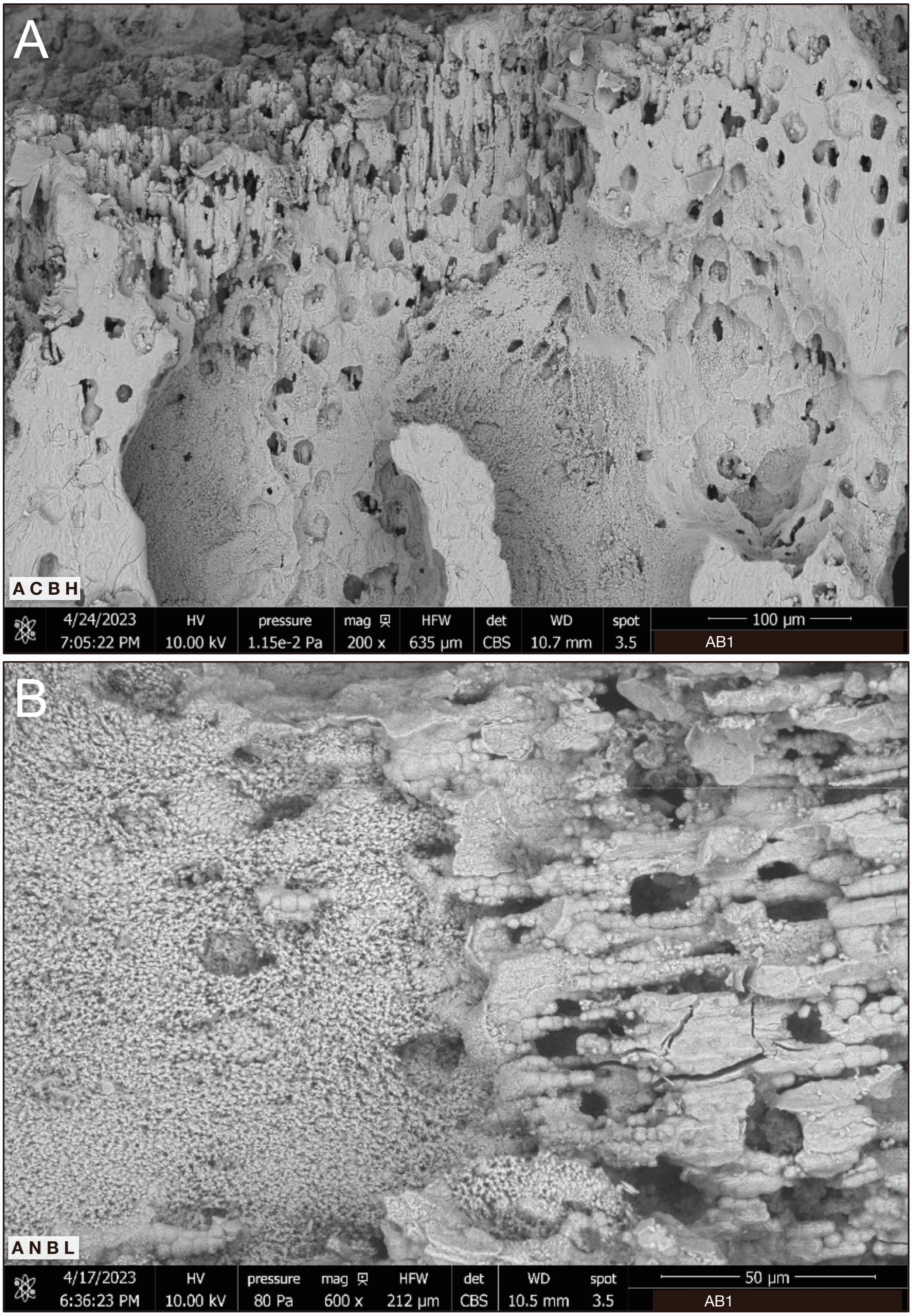

Figure S24. Cartilage and endochondral ossification, AB1, related to Figure 5
(A) Articular cartilage at top of image transitioning to endochondral trabeculae at the bottom of image. Note the distinctive chondrocyte lacunae.
(B) Higher magnification image of a different area. Note empty chondrocyte lacunae and calcospheritic mineralization of cartilage towards right and a forming bone mineralizing front to the left of the field. Scale bar in A equals 100 µm; scale bar in B equals 50 µm.

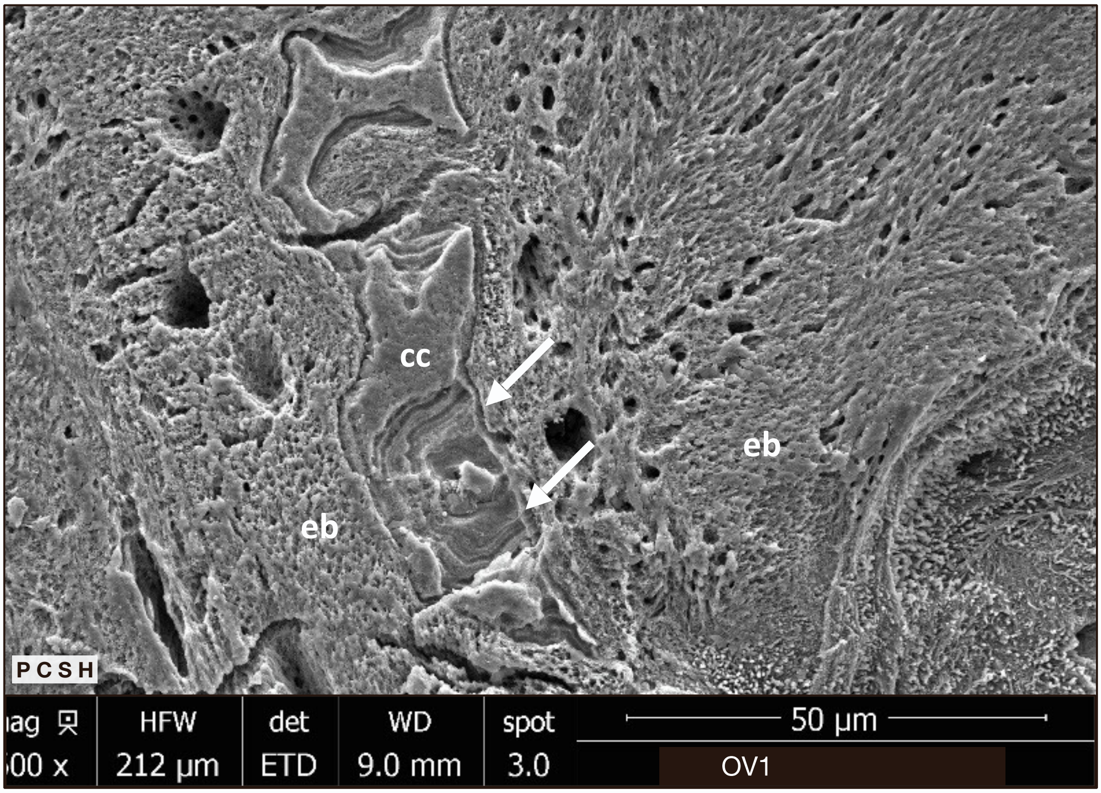

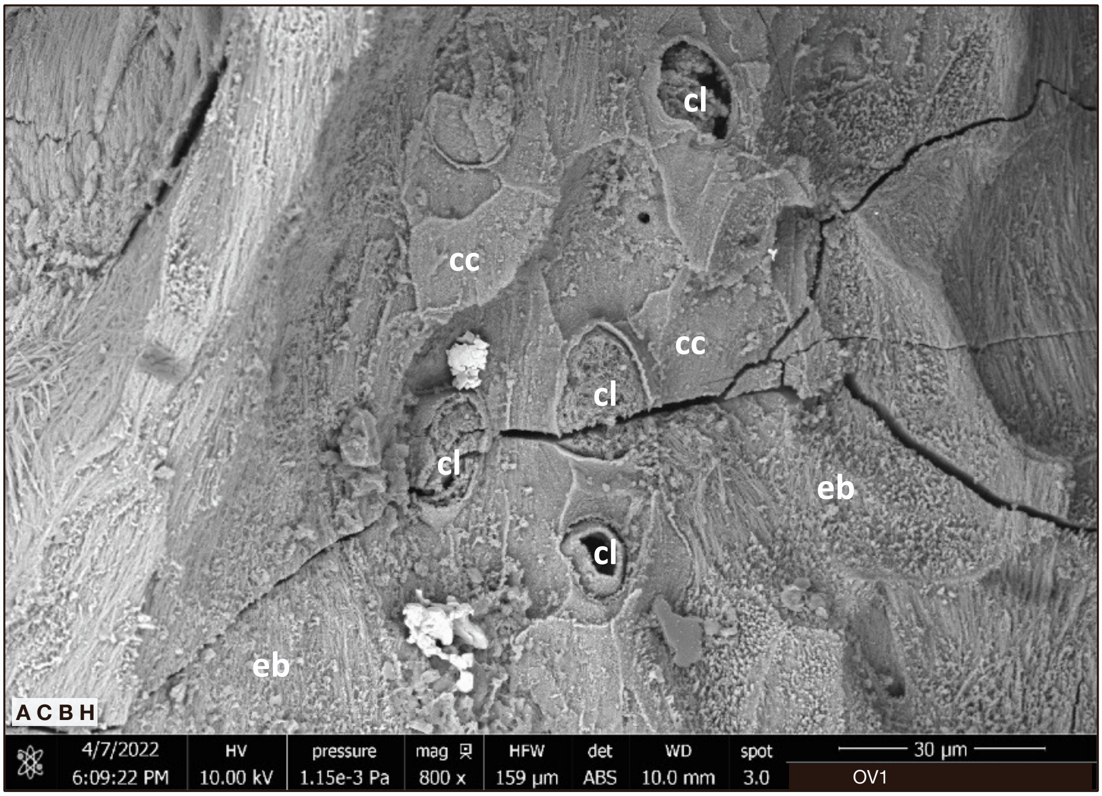

Figure S25. Cartilage and endochondral ossification, OV1, related to Figure 5

(A) Etched surface section with a small island of residual cartilage. Liesegang rings in the mineralized chondrocyte infill revealed by etching are truncated by osteoclast activity, indicating a biological origin of mineralization. Arrow points to resorption of Liesegang rings.

(B) Resorption surface on cartilage and endochondral bone matrix. Osteoclast activity opened up chondrocyte lacunae that now have a diagenetic mineral infill. Abbreviations: cc, calcified cartilage matrix; cl, chondrocyte lacunae, eb, endochondral bone.

Scale bar in A equals 50 µm; scale bar in B equals 30 µm.

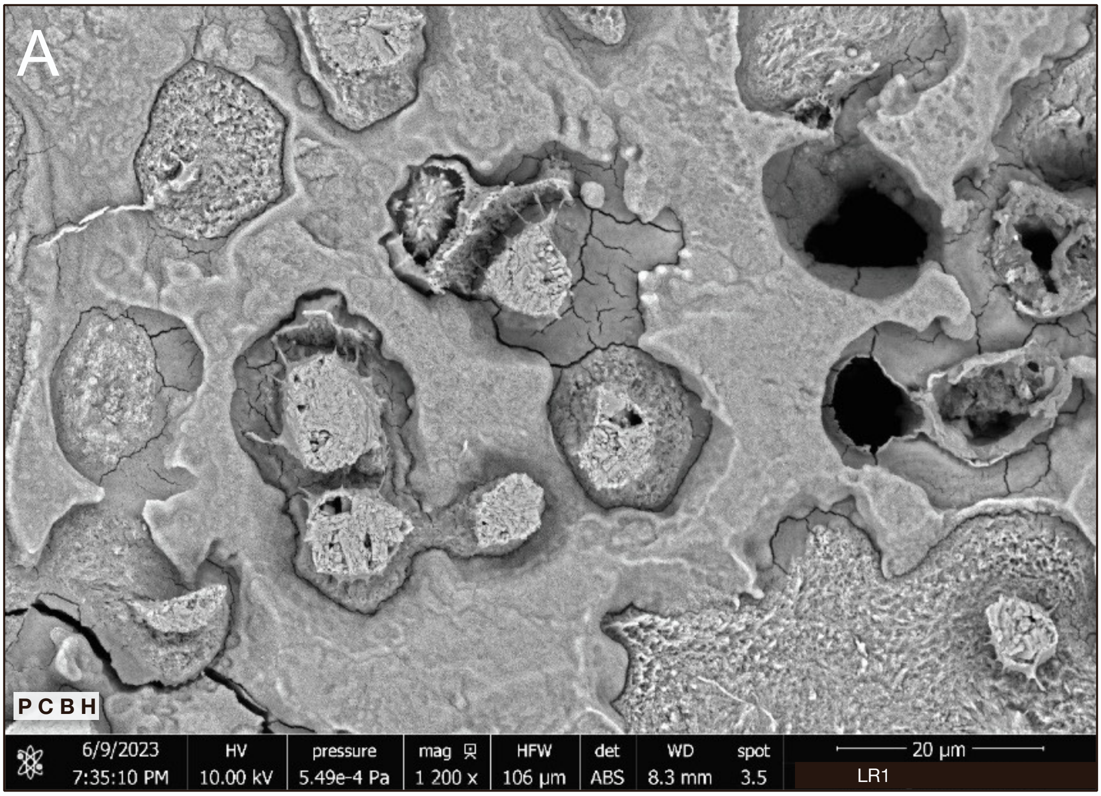

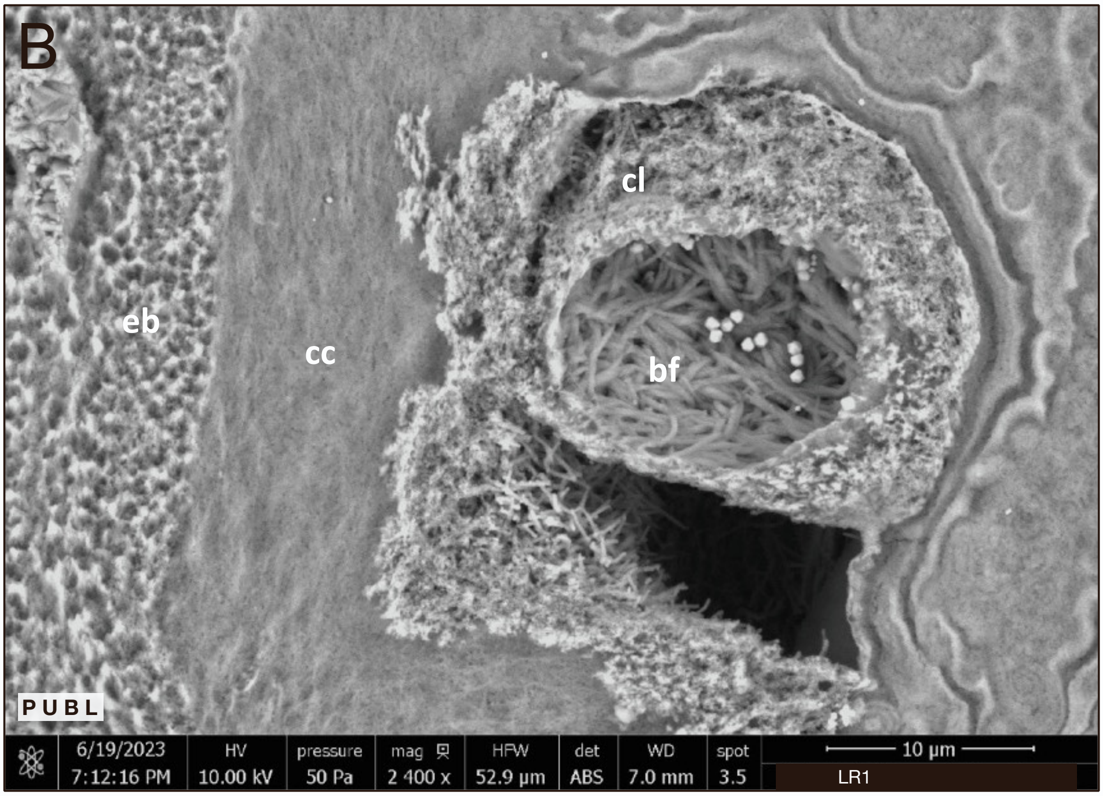

Figure S26. Endochondral ossification: osteocyte casts inside former chondrocyte lacunae on etched section surface, LR1, related to Figure 5
(A) Several former chondrocyte lacunae in a calcified cartilage matrix with osteocyte lacuna casts inside. Note the filopodia (canalicular processes) of the latter and their diagenetic mineral fill. Higher resolution version of Figure 5G,
(B) Former chondrocyte lacuna with with typical bone collagen fibrils inside. Abbreviations: bf, bone fibrils; cc, calcified cartilage matrix; cl, chondrocyte lacunae, eb, endochondral bone.
Scale bar in A equals 20 µm; scale bar in B equals 10 µm.

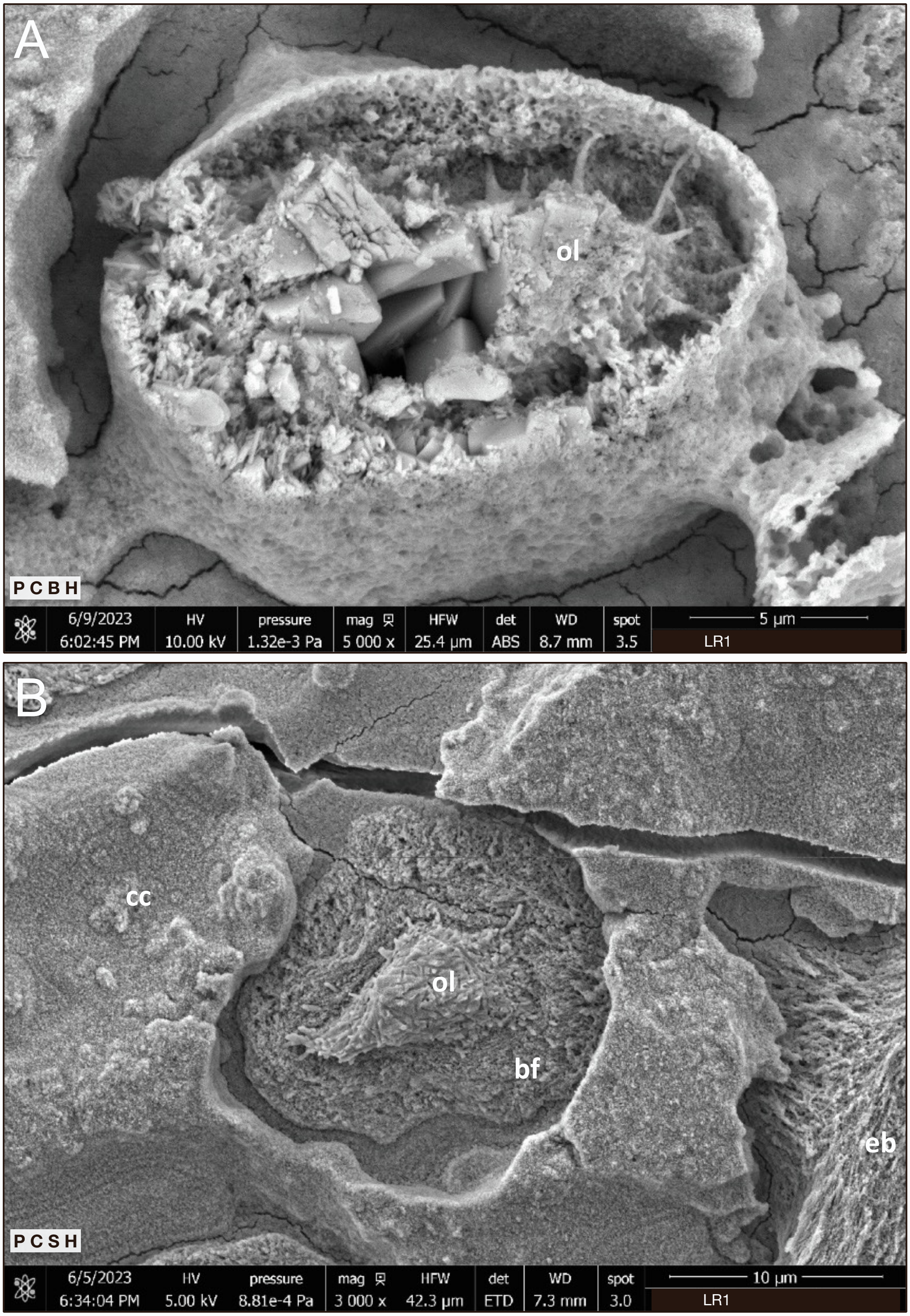

Figure S27. Endochondral ossification: osteocyte casts inside former chondrocyte lacunae on etched section, LR1, related to Figure 5

(A) Chondrocyte lacuna containing an osteocyte lacuna with a diagenetic mineral infill. The lining of the chondrocyte wall may either be diagenetic in origin or might represent a pericellular capsule.

(B) Chondrocyte lacuna with an osteocyte cast inside. Note the bone fibril impressions on the ‘osteocyte’ surface, indicating that the osteocyte was set in a bone matrix and its lacuna is a natural cast. Abbreviations: bf, bone fibrils; cc, calcified cartilage matrix; cl, chondrocyte lacunae, eb, endochondral bone; ol, osteocyte lacuna.

Scale bar in A equals 5 µm; scale bar in B equals 10 µm.

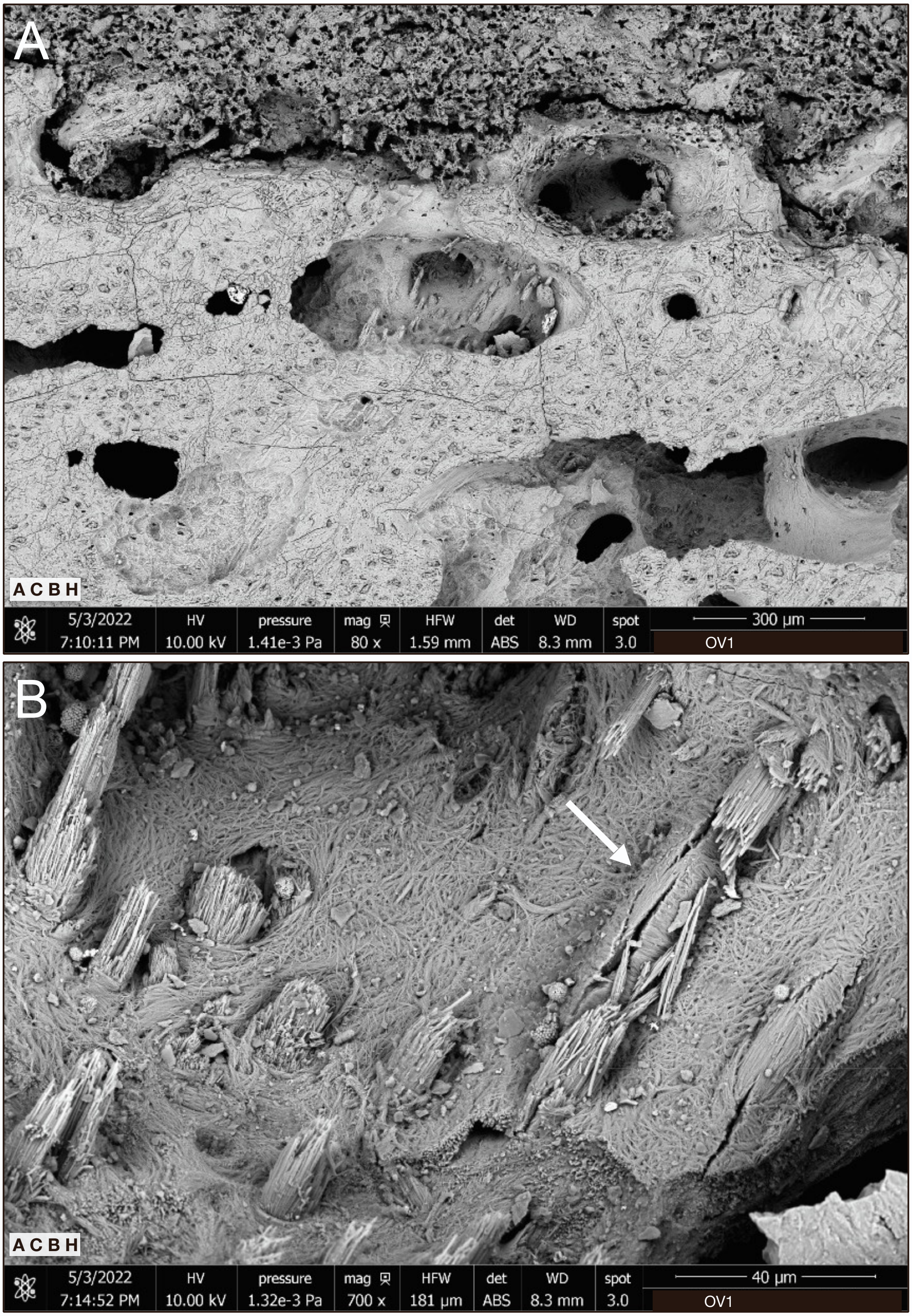

Figure S28. Sharpey (extrinsic) fibers crossing a primary trabecular space in the periosteal territory, OV1, related to Figure 5

(A) Sharpey fibers are visible in the etched surface and continue into the open trabecular spaces. Bone tissue at top was degraded postmortem. Overview of area in main text Figure 4I.

(B) Close-up, note how the intrinsic bone fibers wrap around the Sharpey fibers (arrow).

Scale bar in A equals 300 µm; scale bar in B equals 40 µm.

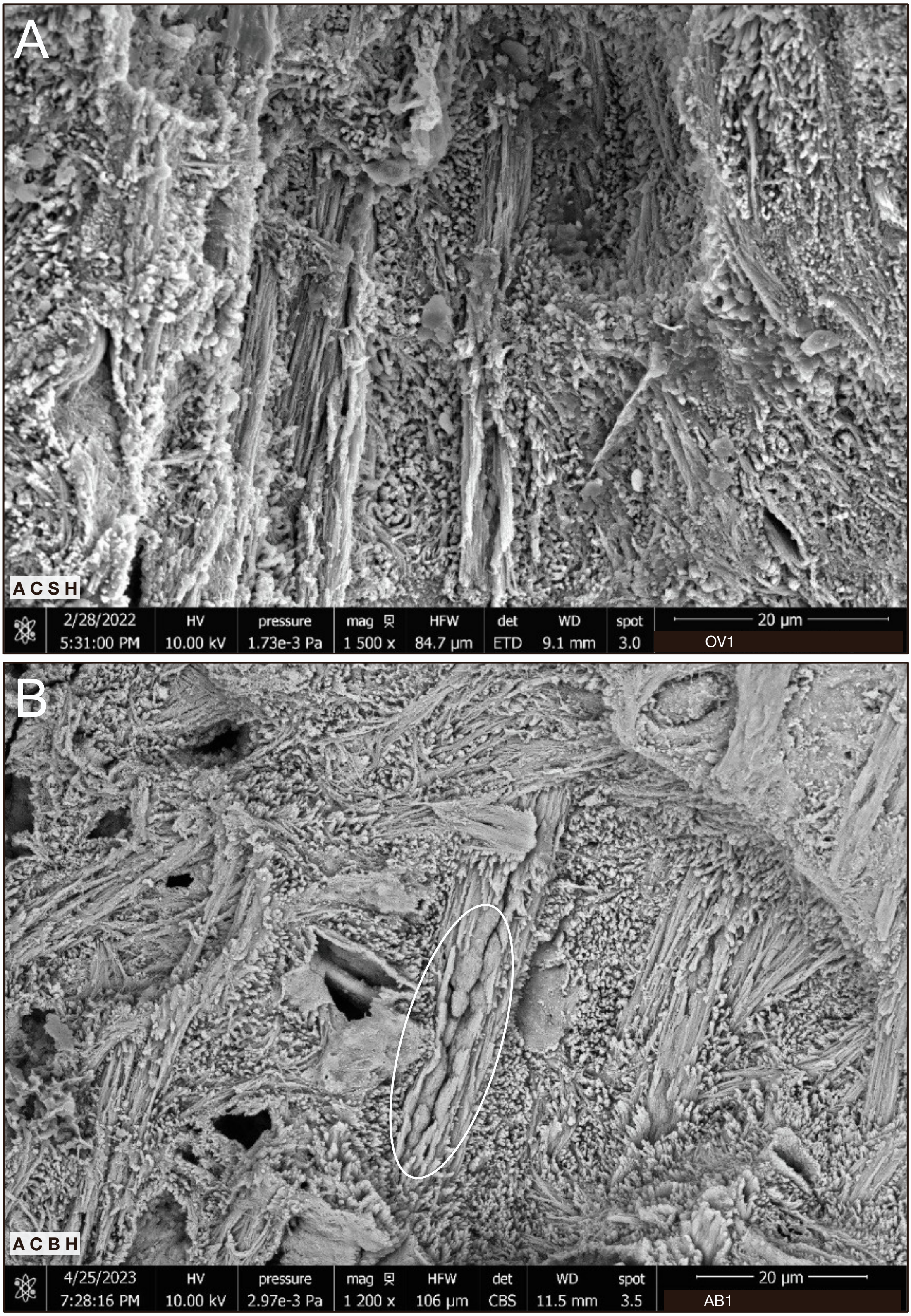

Figure S29. Center of Sharpey fibers exposed by osteoclast activity, related to Figures 4 and 5
(A) OV1, resorbed internal trabecular surfaces reveal Sharpey fibers partially unmineralized at the center.
(B) AB1, note the incompletely mineralized interior of the fibers. Zooming into the image reveals lozenge-shaped bodies (those parts of the fiber bundles which were mineralized in life) as in the forming surfaces (inside white oval).
Scale bars in A, B equal 20 μm.

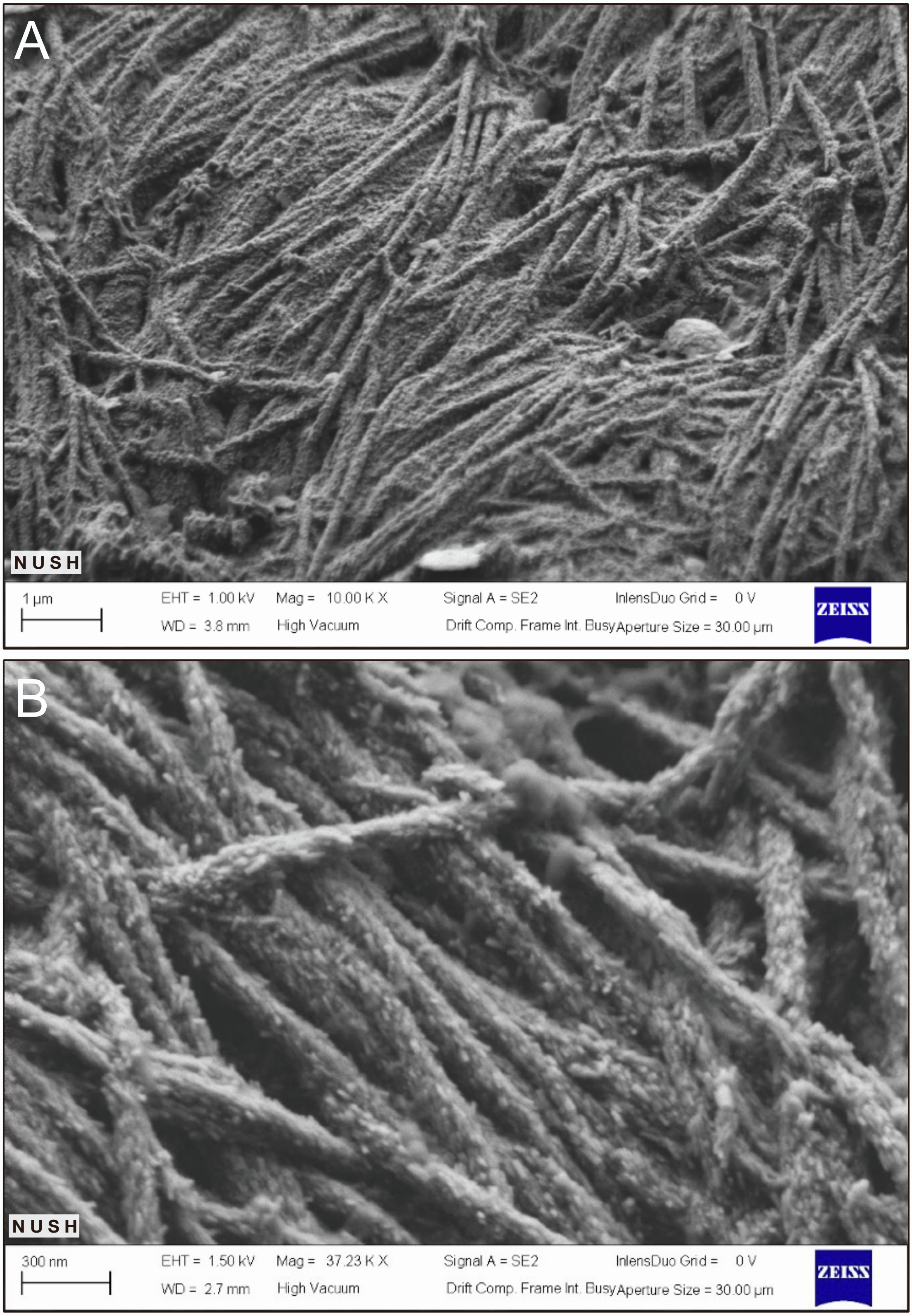

Figure S30. Bone fibrils observed in uncoated sample at high magnification, OV1, related to Figure 6
(A) Bone fibrils are covered by diagenetic apatite nanocrystals.
(B) Higher magnification view, same frame as in main text Figure 5C.
Scale bar in A equals 1 µm; scale bar in B equals 300 nm

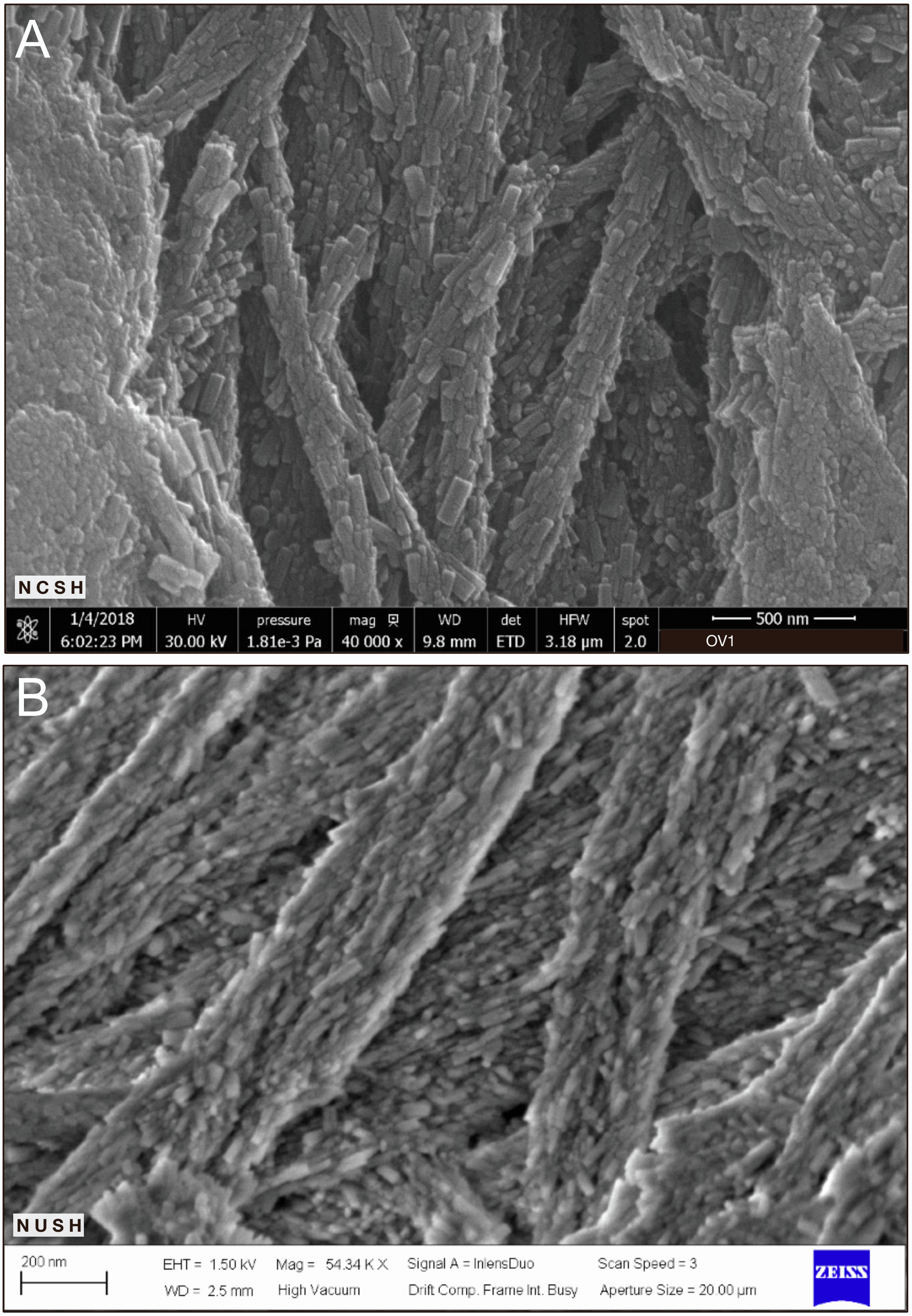

Figure S31. Bone fibrils observed at high magnification, OV1, related to Figure 6
(A) Bone fibrils are covered by or consist of relatively large diagenetic apatite nanocrystals. Thinly coated sample. Higher resolution version of image in main text Figure 5D.
(B) Similar view but taken with an in-lens detector of the Zeiss Merlin microscope of an uncoated sample.
Scale bar in A equals 500 nm; scale bar in B equals 200 nm.

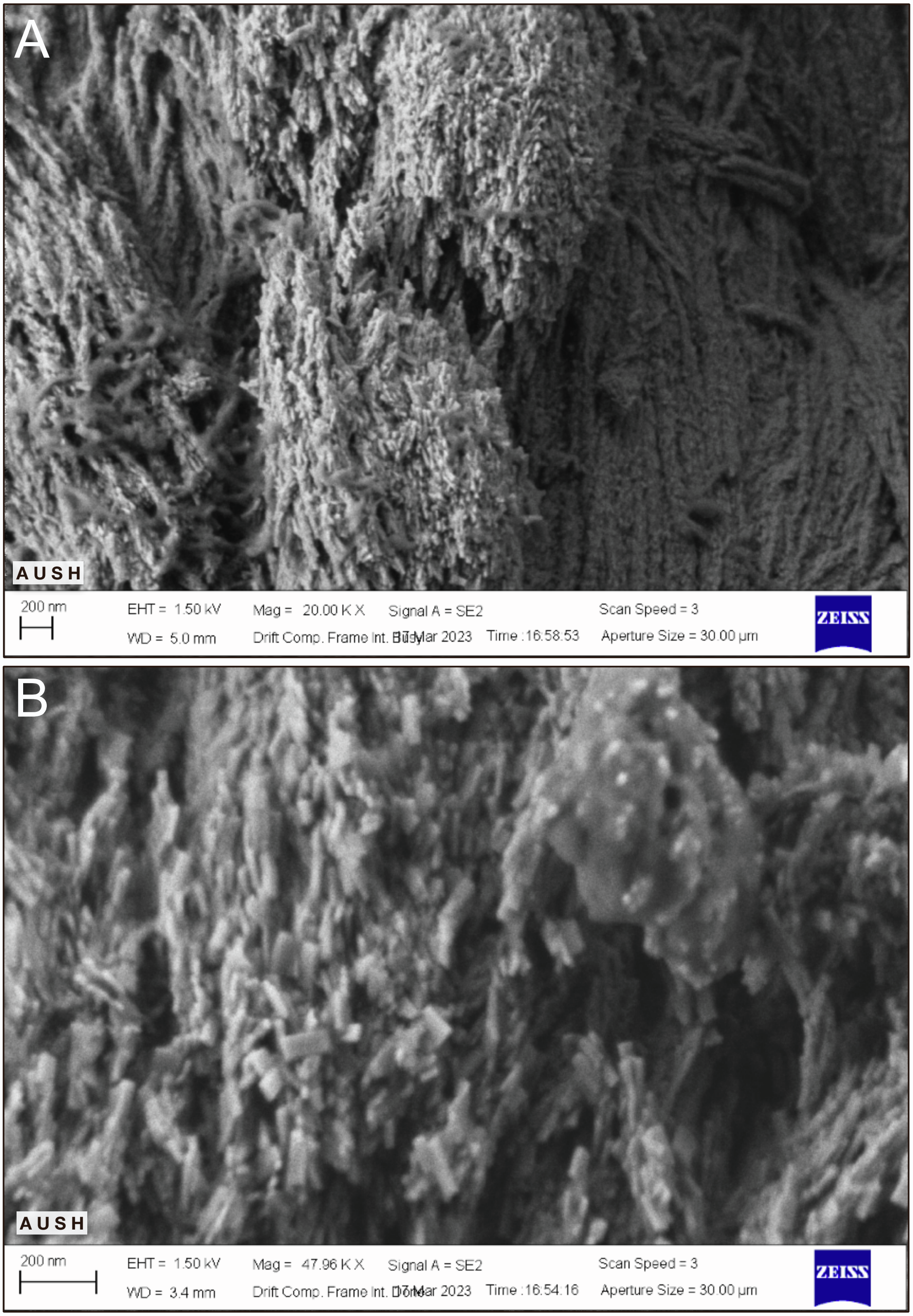

Figure S32. Forming surfaces at high magnification, uncoated sample, OV2, related to Figure 6
(A) Enlargement of lozenge-shaped bodies in a mineralizing front in a forming surface, resting surface in background.
(B) Further enlargement, note the platelet-shaped nanocrystals.
Scale bars in A, B equal 2000 nm.

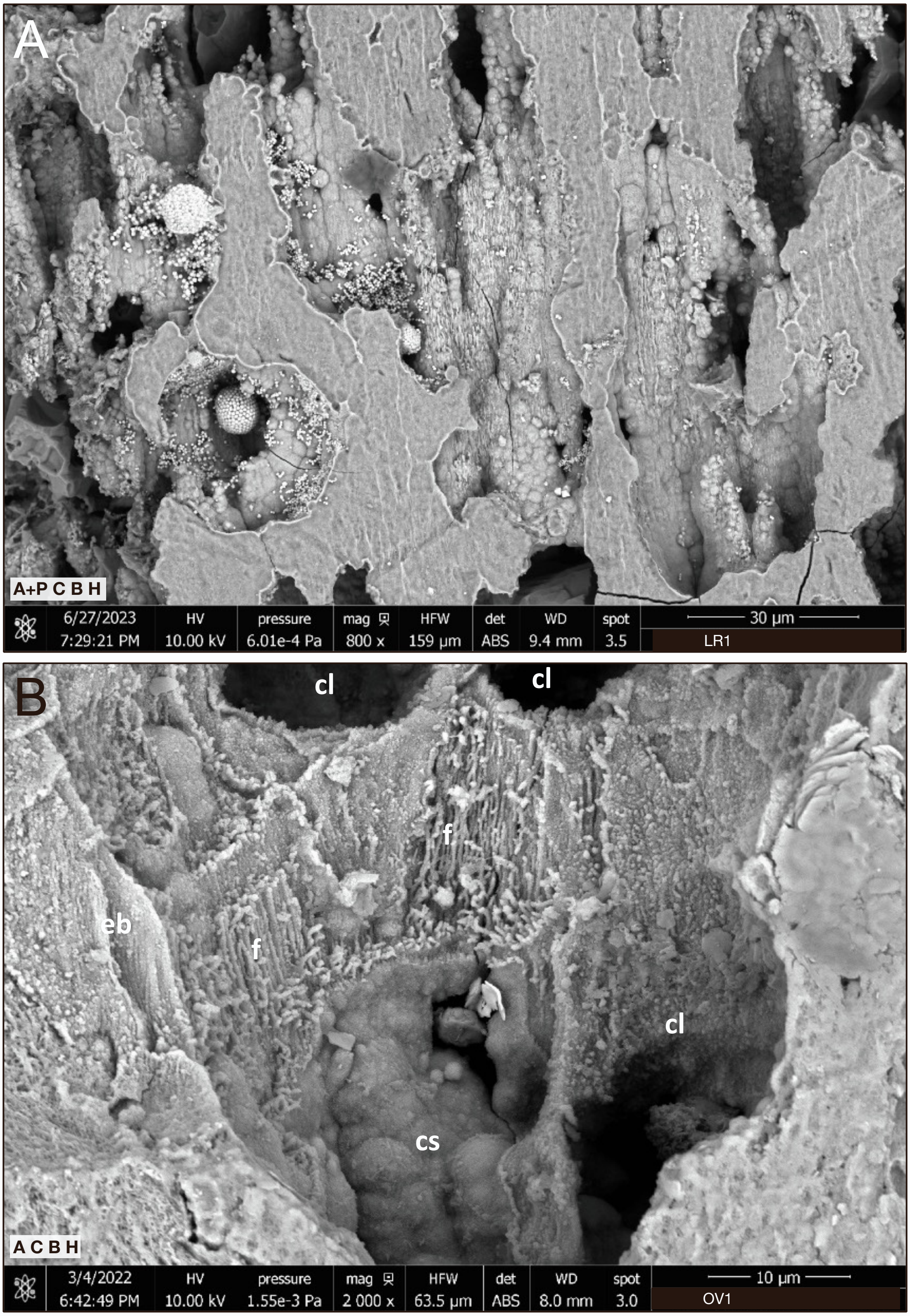

Figure S33. Cartilage mineralized collagen fibers, related to Figures 5 and 6
(A) LR1, fibers partially overgrown by calcospherites in pore space and, less distinctively, in etched surface. Section surface etched with phosphoric acid.
(B) OV1, fibers in cartilage matrix revealed by osteoclast activity. Abbreviations: cl, chondrocyte lacuna; cs, calcospherites; f, cartilage fibrils.
Scale bar in A equals 30 μm; scale bar in B equals 10 μm.

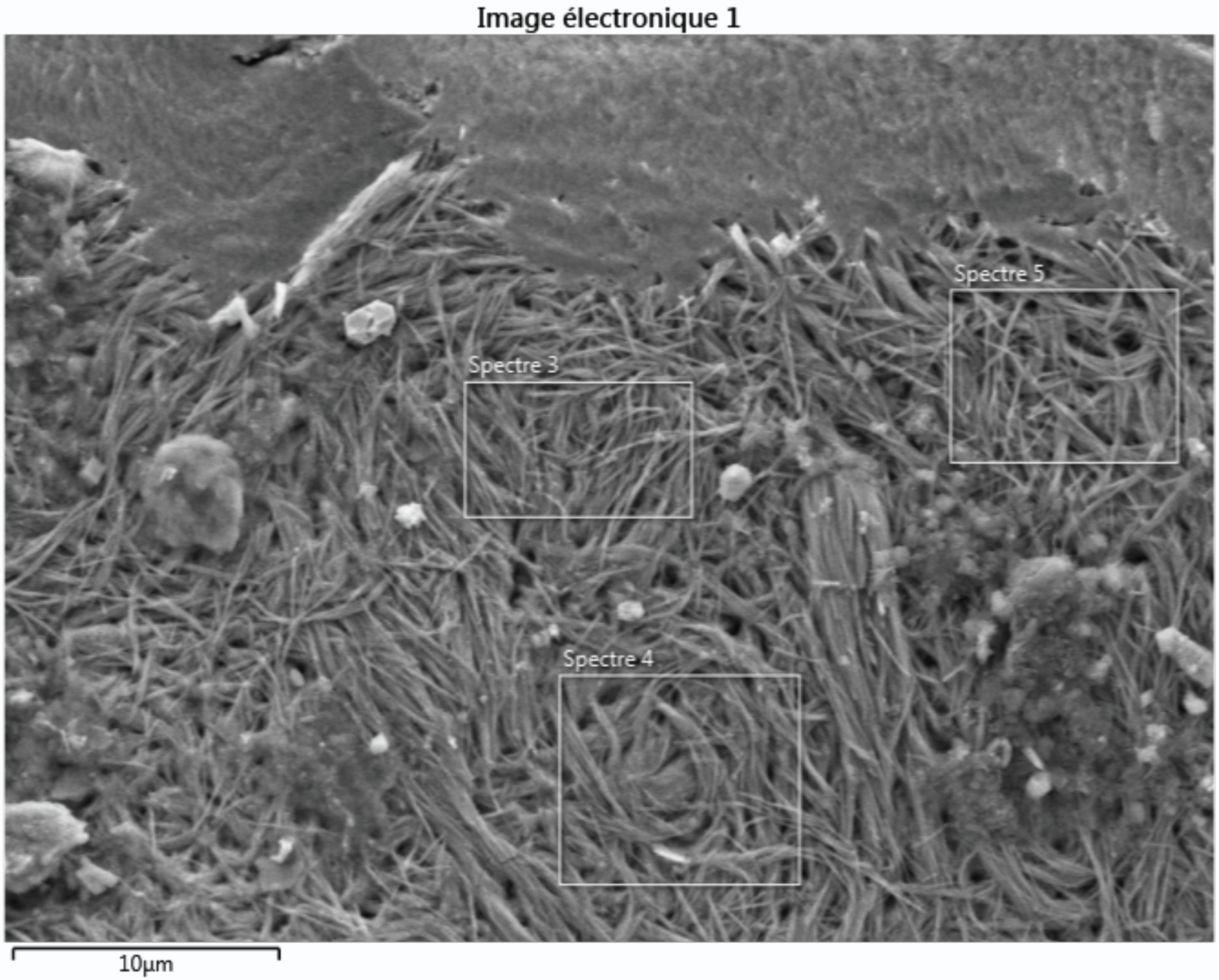

Figure S34: EDX analyses of sample OV1, bone fibrils, related to STAR Methods. Untreated resting surface with bone fibrils; the smooth region on top is a fracture surface. Distribution of EDX sampling areas 3 to 5 (“Spectre”). The sample was coated with carbon and analyzed at 8 kV. Scale bar equals 10 µm.

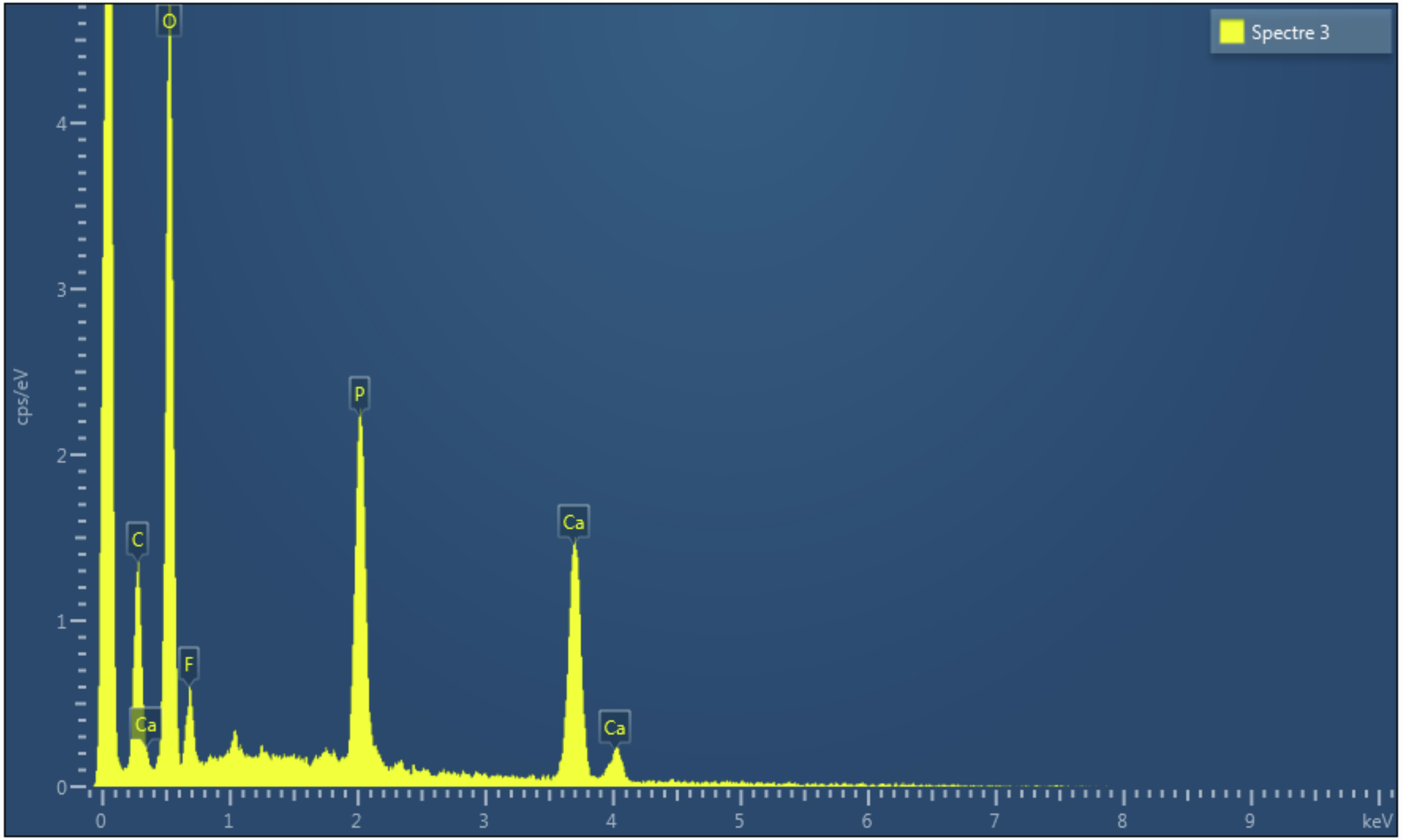

Figure S35: EDX analyses of sample OV1, bone fibrils, related to STAR Methods. Spectrum 3.

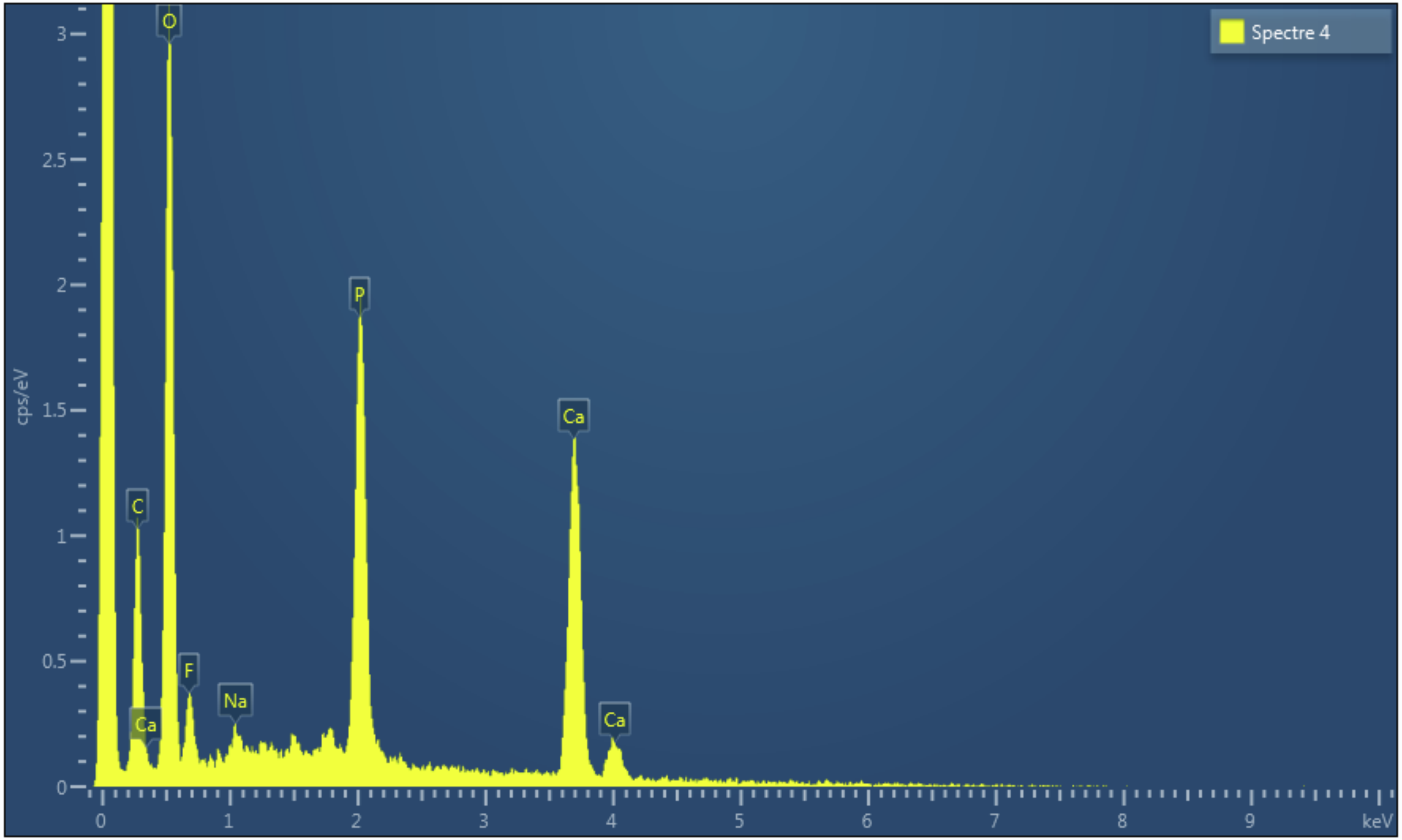

Figure S36: EDX analyses of sample OV1, bone fibrils, related to STAR Methods. Spectrum 4.

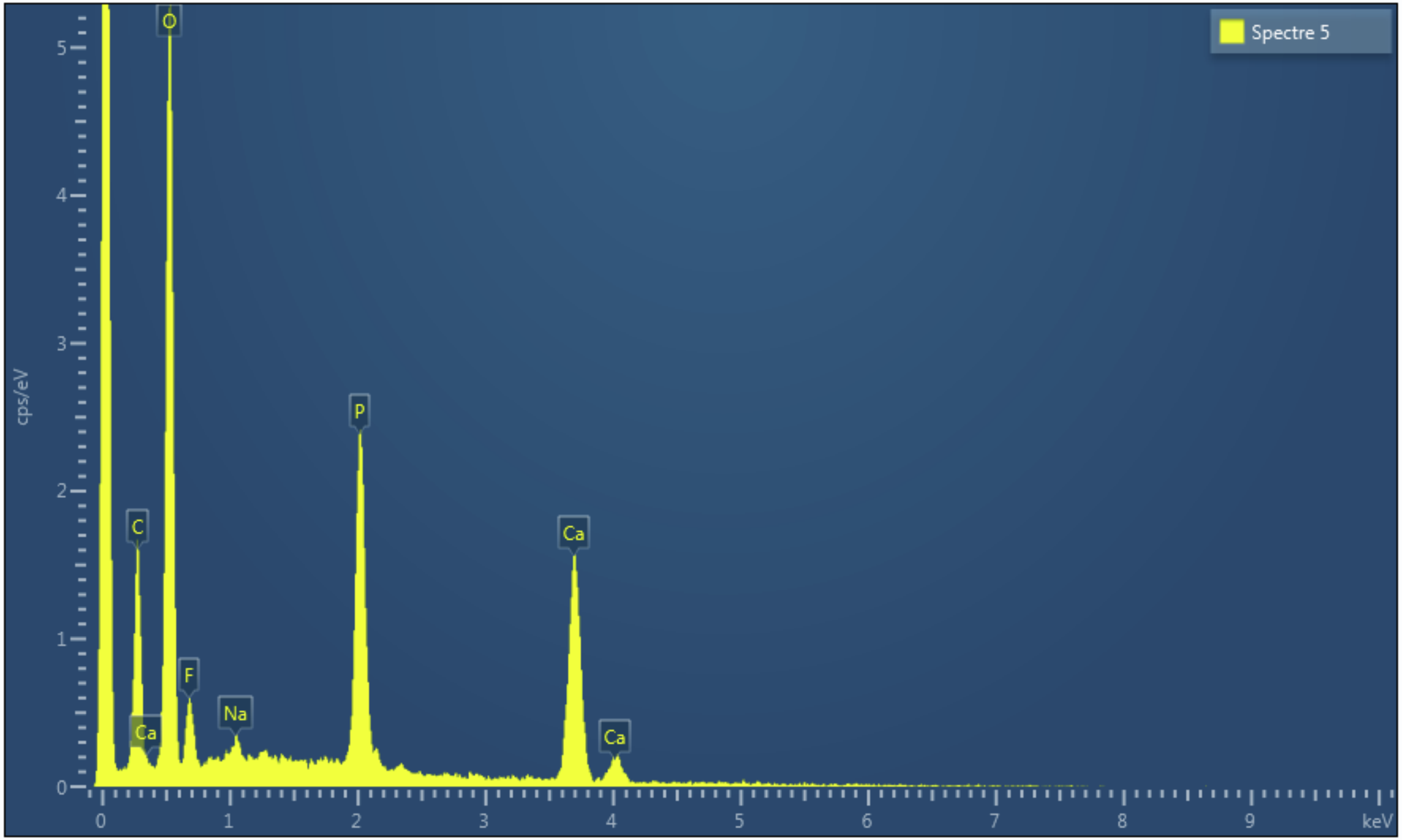

Figure S37: EDX analyses of sample OV1, bone fibrils, related to STAR Methods. Spectrum 5.

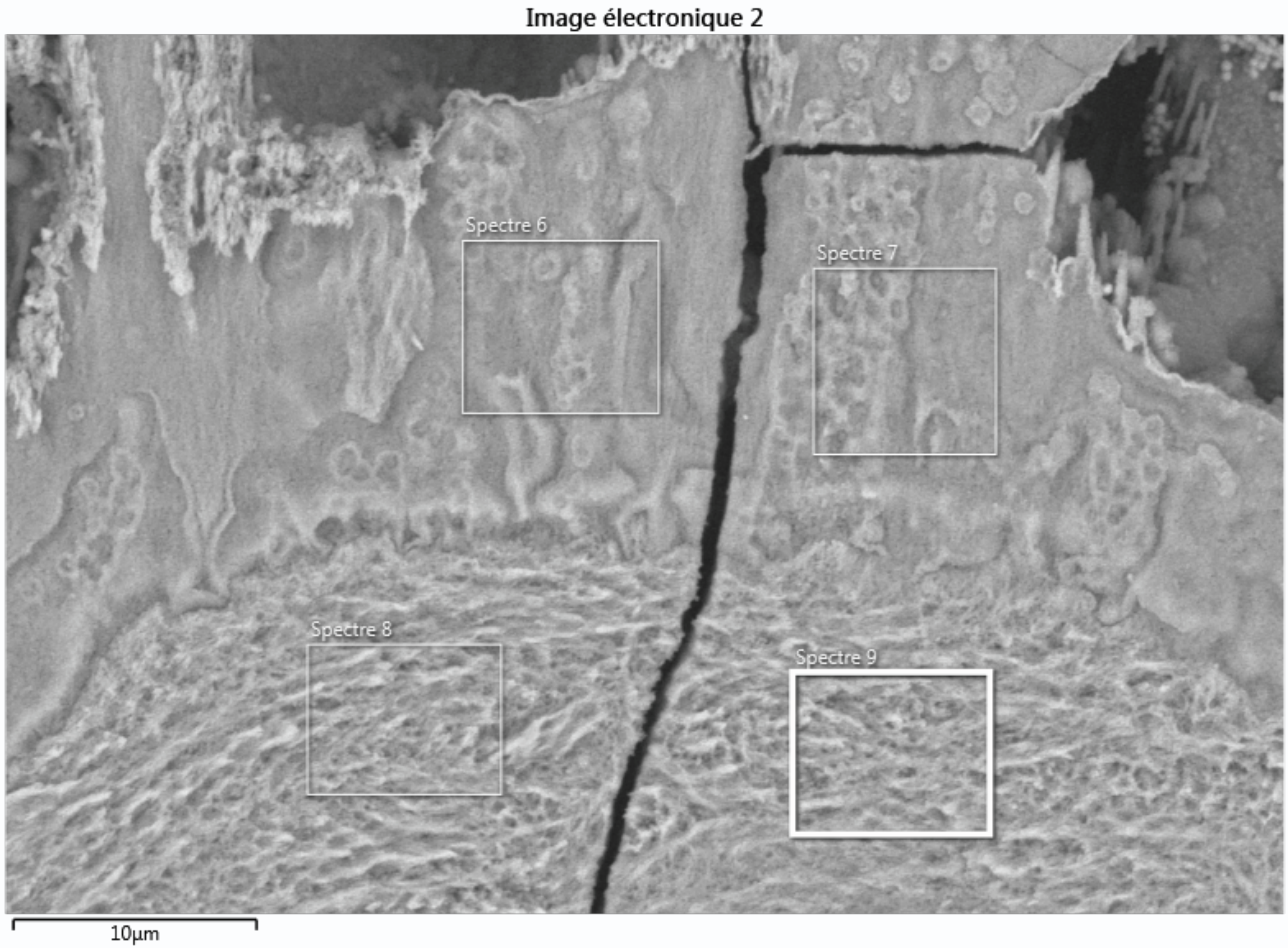

Figure S38: EDX analyses of sample LR1, bone matrix and cartilage, related to STAR Methods. Sample sectioned and then surface polished and etched, distribution of EDX sampling areas 6 to 9 (“Spectre”). The sample was coated with Au/Pd and analyzed at 10 kV. Scale bar equals 10 µm.

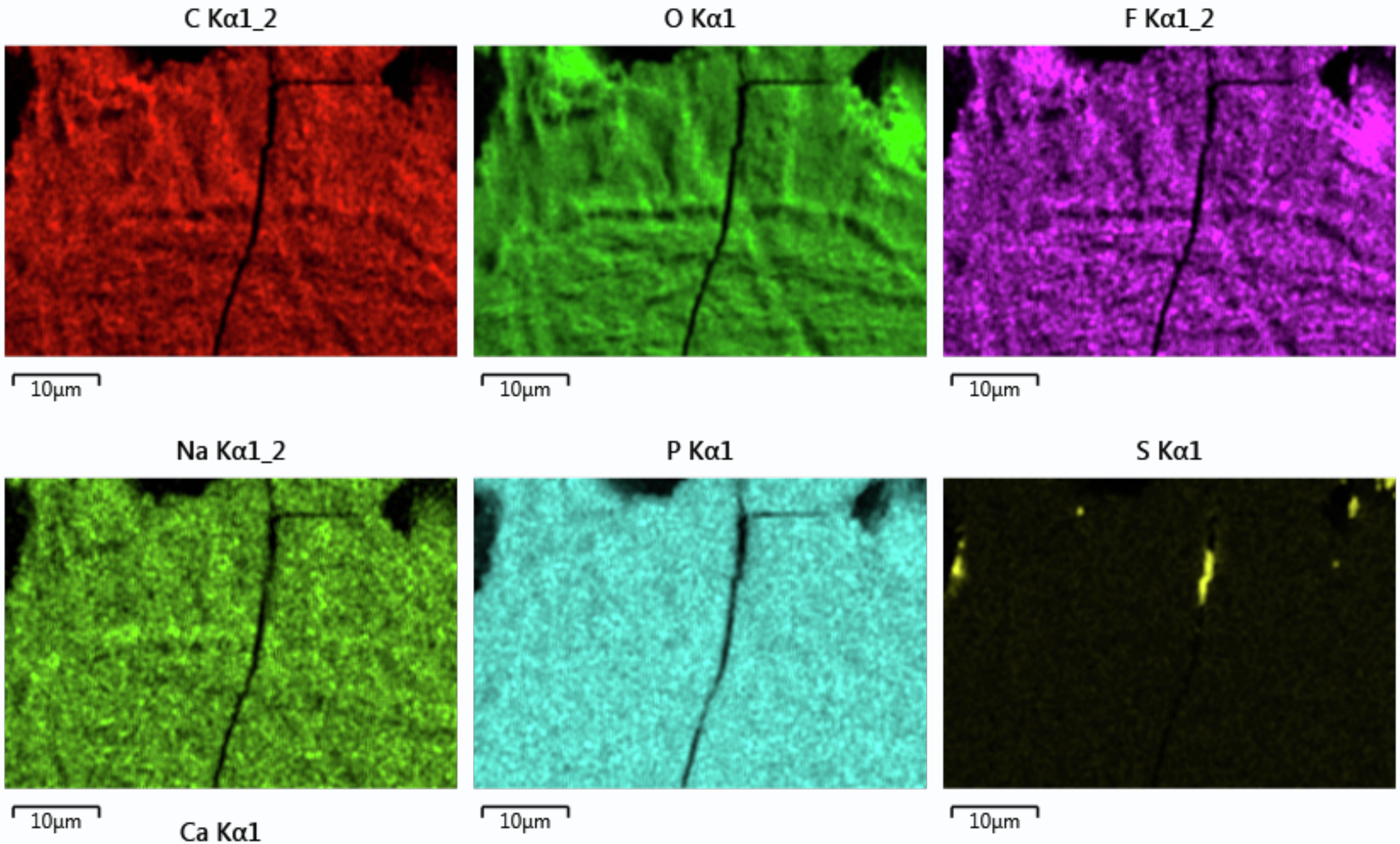

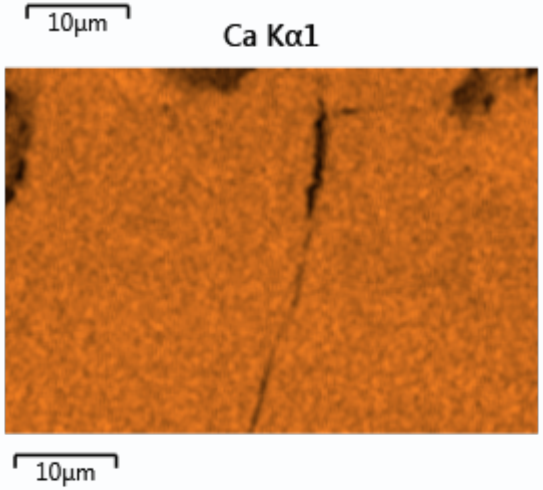

Figure S39: EDX analyses of sample LR1, bone matrix and cartilage, related to STAR Methods. Element maps of the field of view in Figure 1 for select elements, indicating that the entire field of view consists of apatite, including both the bone matrix and the cartilage. Scale bar equals 10 µm.

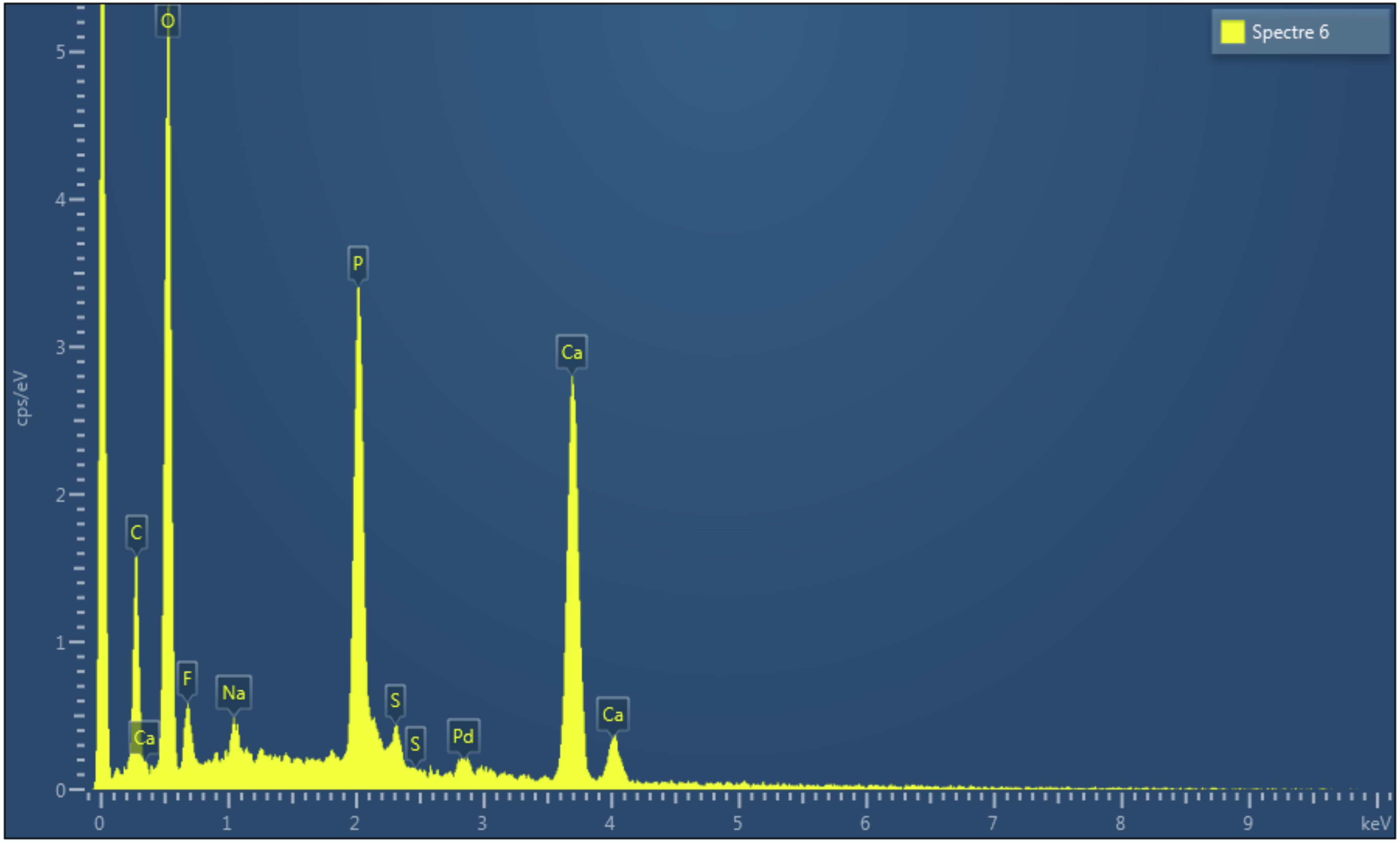

Figure S40: EDX analyses of sample LR1, bone matrix and cartilage, related to STAR Methods. Spectrum 6, of mineralized cartilage.

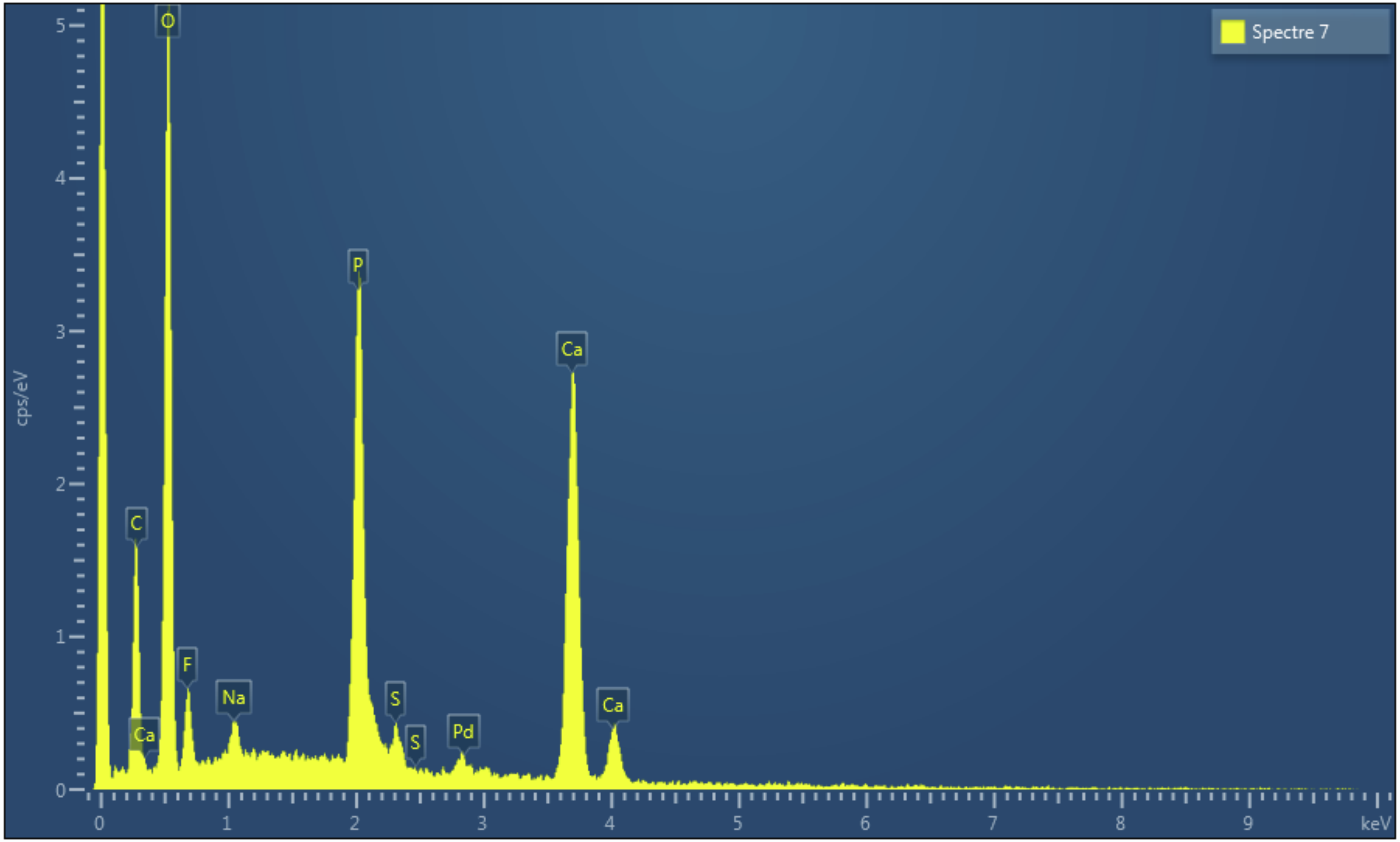

Figure S41: EDX analyses of sample LR1, bone matrix and cartilage, related to STAR Methods. Spectrum 7, of mineralized cartilage.

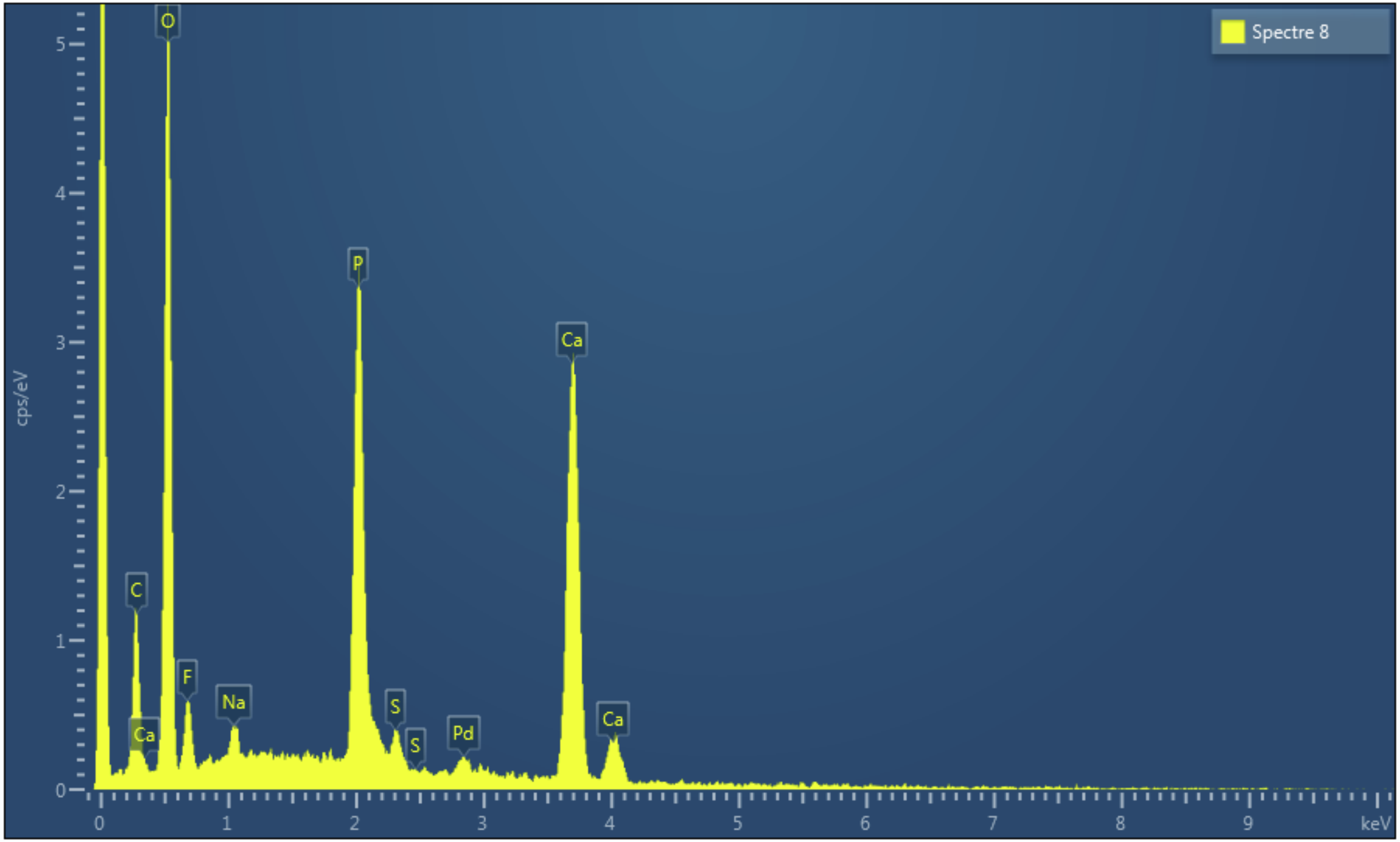

Figure S42: EDX analyses of sample LR1, bone matrix and cartilage, related to STAR Methods. Spectrum 8, of bone matrix.

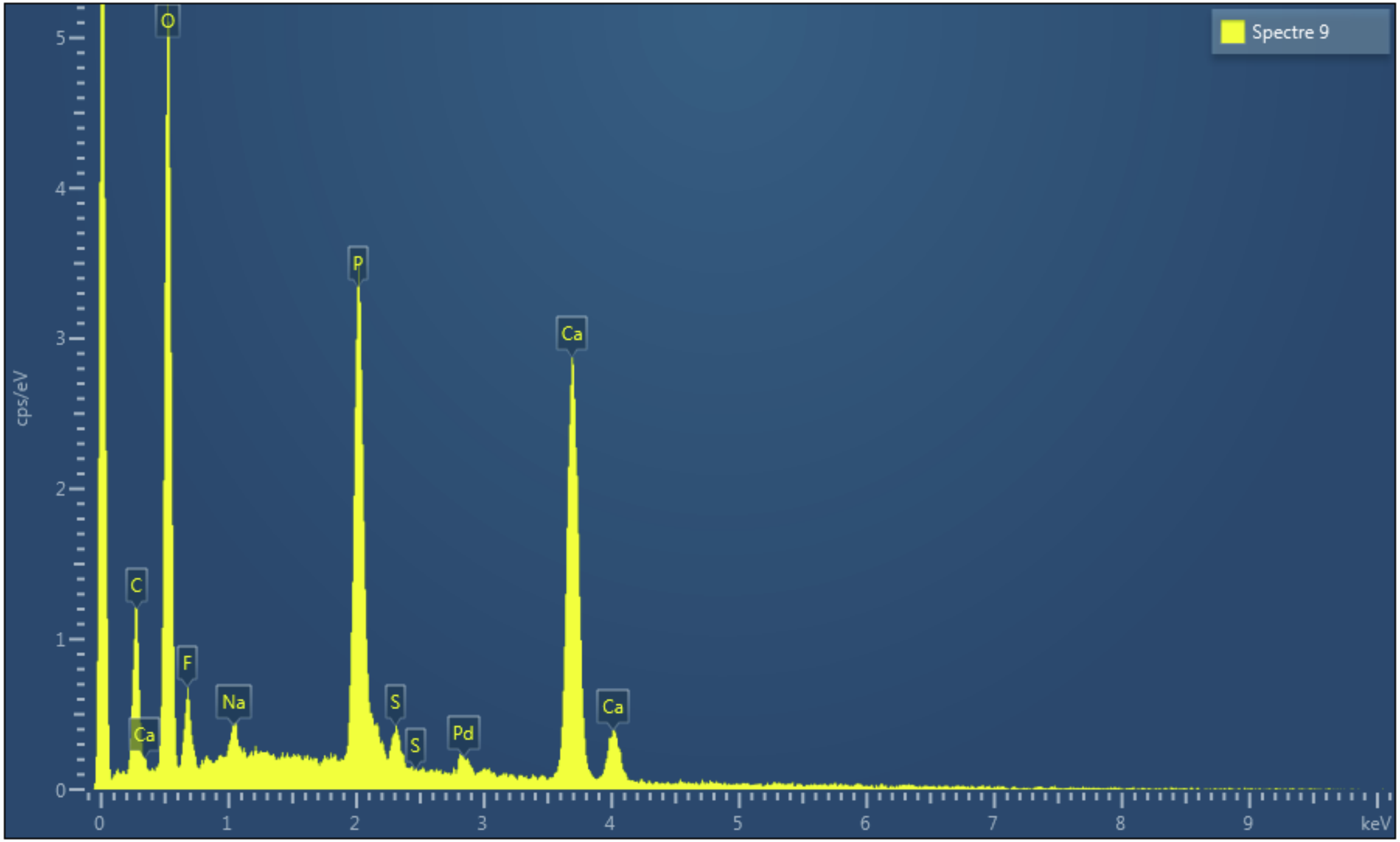

Figure S43: EDX analyses of sample LR1, bone matrix and cartilage, related to STAR Methods. Spectrum 9, of bone matrix.

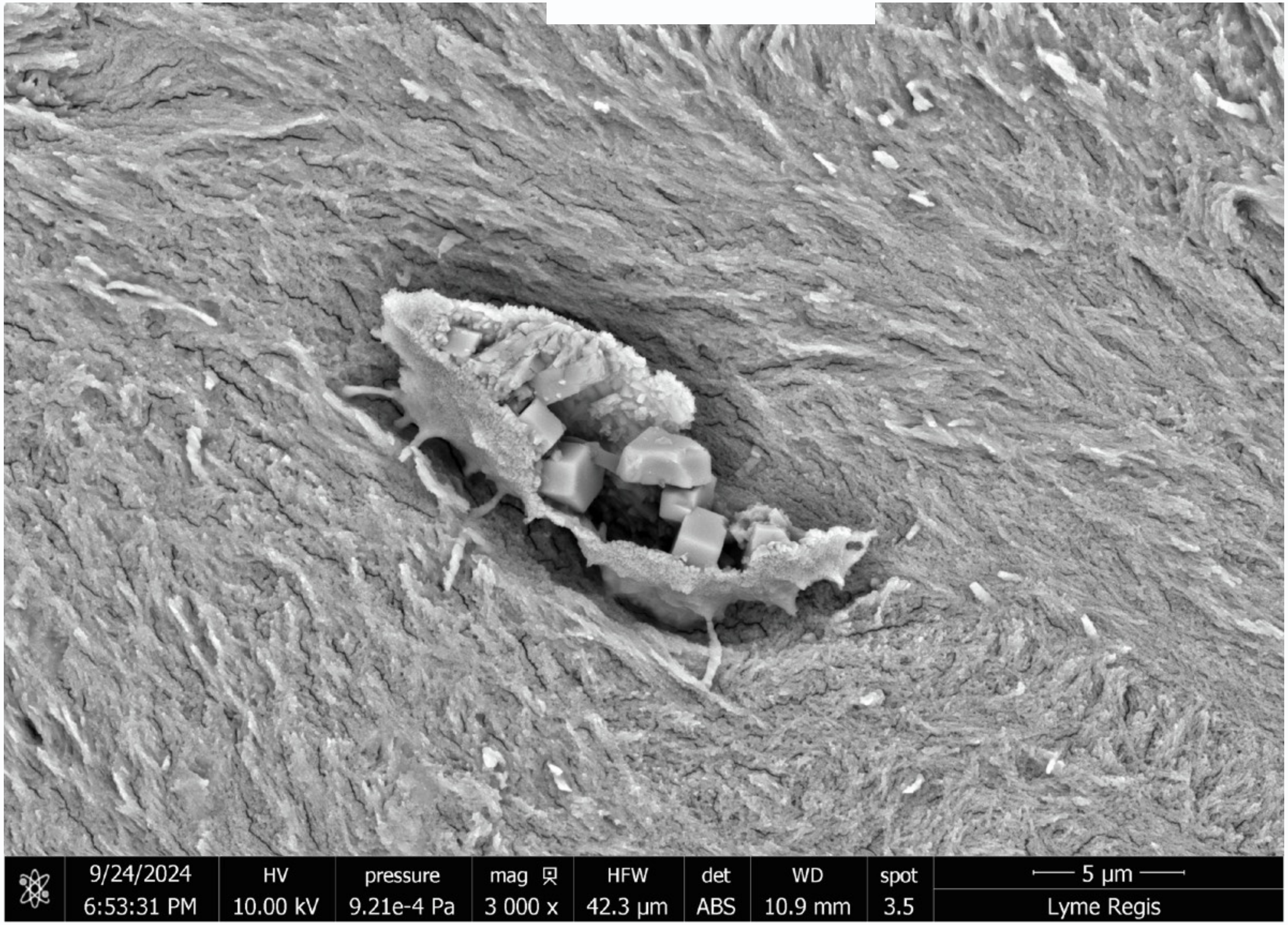

Figure S44: EDX Analysis of Sample LR1, Osteocyte, related to STAR Methods. Etched surface revealing the shell and interior of an osteocyte or lacunar cast. Note the large crystals. Scale bar equals 5 µm.

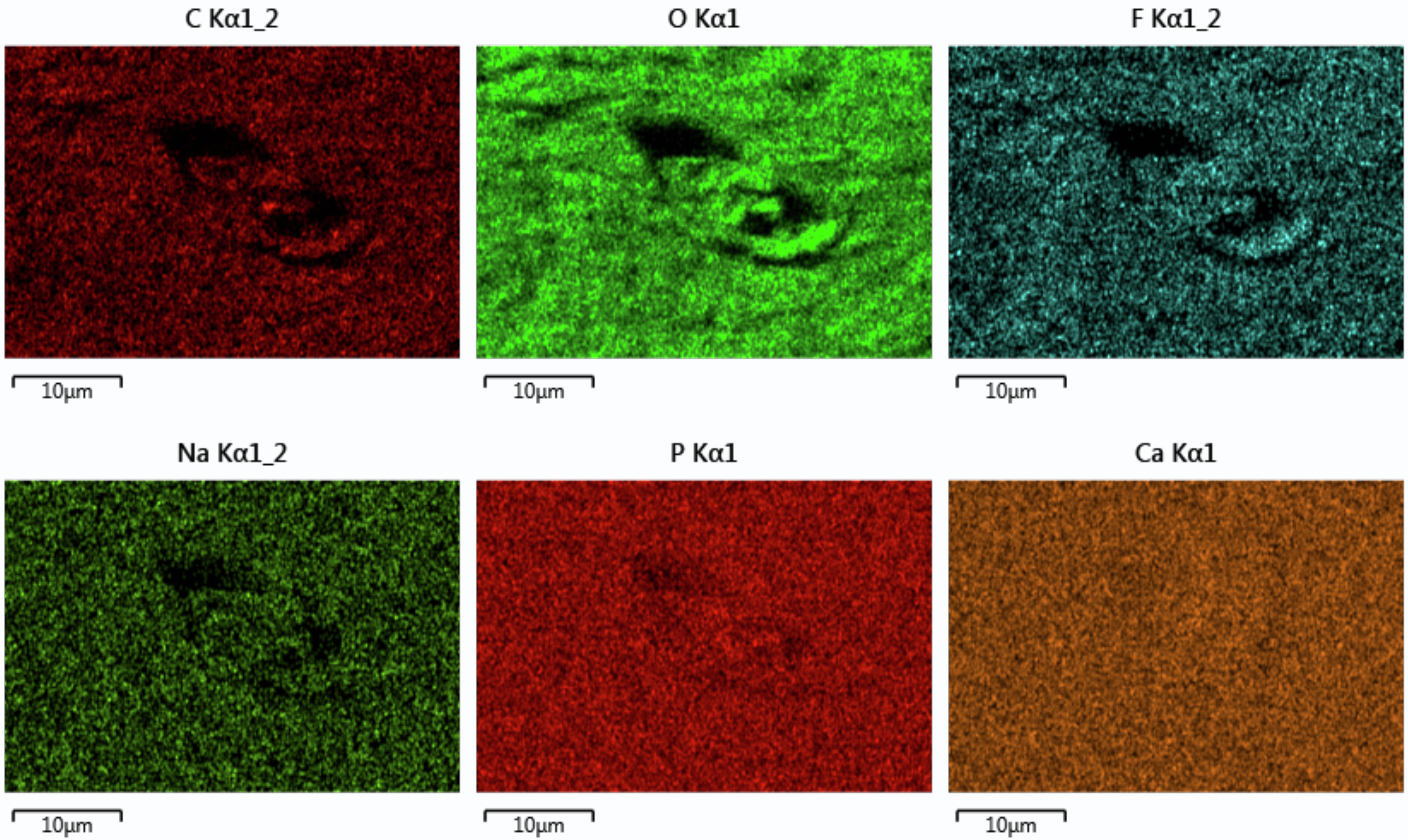

Figure S45: EDX Analysis of Sample LR1, Osteocyte, related to STAR Methods. Element maps of the area in Figure 1 for select elements, indicating that the entire field of view consists of apatite, including the osteocyte/lacunar cast. Scale bar equals 10 µm.

Image électronique 1

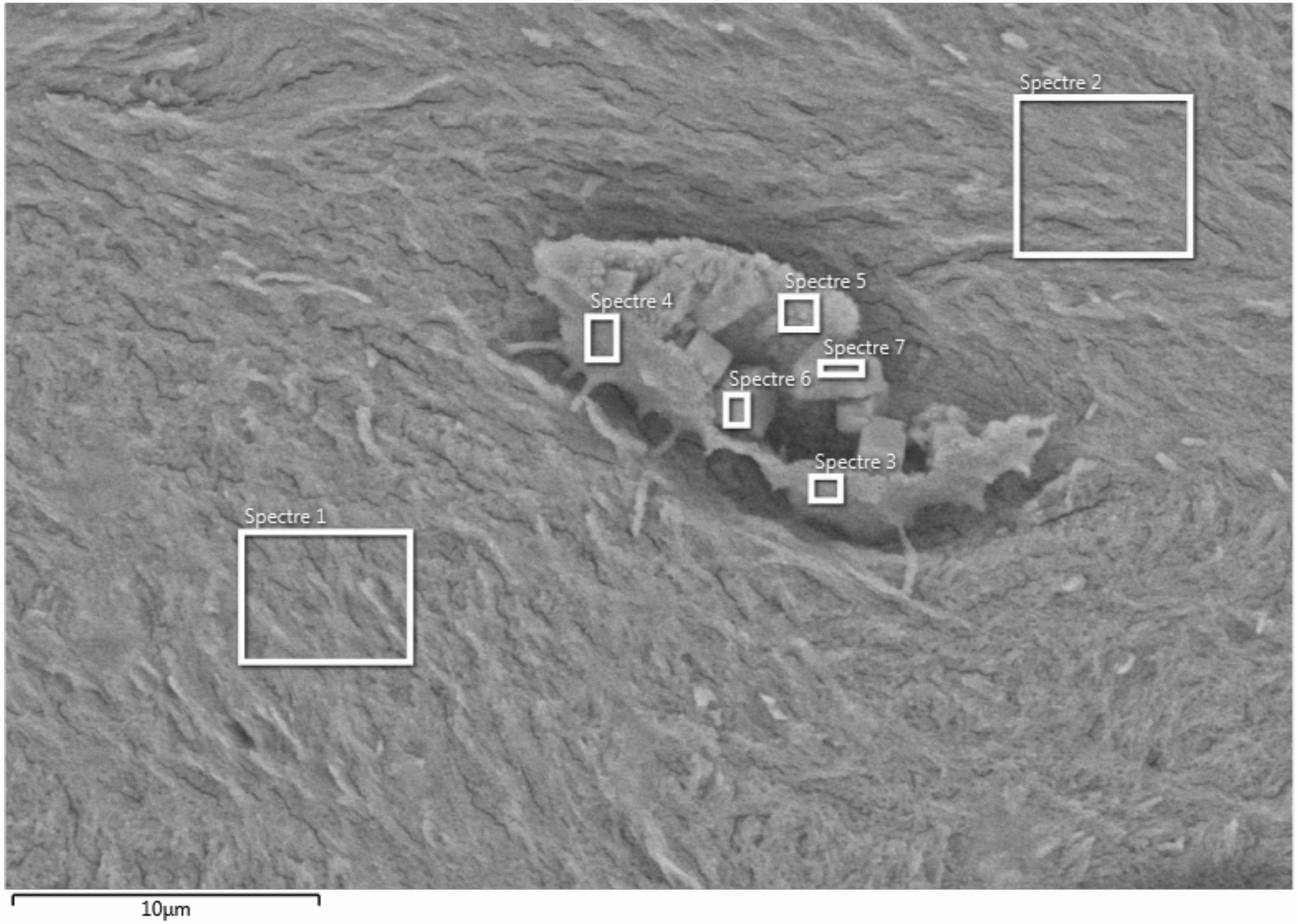

Figure S46: EDX Analysis of Sample LR1, Osteocyte, related to STAR Methods. Distribution of EDX sampling areas 1 to 7 (“Spectre”), same field of view as in previous figures. Note that the EDX spatial resolution is lower than that of the SEM image, affecting the accuracy of the results for sampling areas 3 to 7 . For one, the relative atomic compositions (or weight percentages) and relative intensities of EDX peaks are affected by the relief of the sample. Furthermore, the X-ray emission zones, particularly those of the K alpha lines of calcium and phosphorus, correspond to much larger areas both on the surface and at depth than the emission zone of the secondary or backscattered electrons, which is the origin of the SEM image. The sample was coated with Au/Pd and analyzed at 10 kV. Scale bar equals 10 µm.

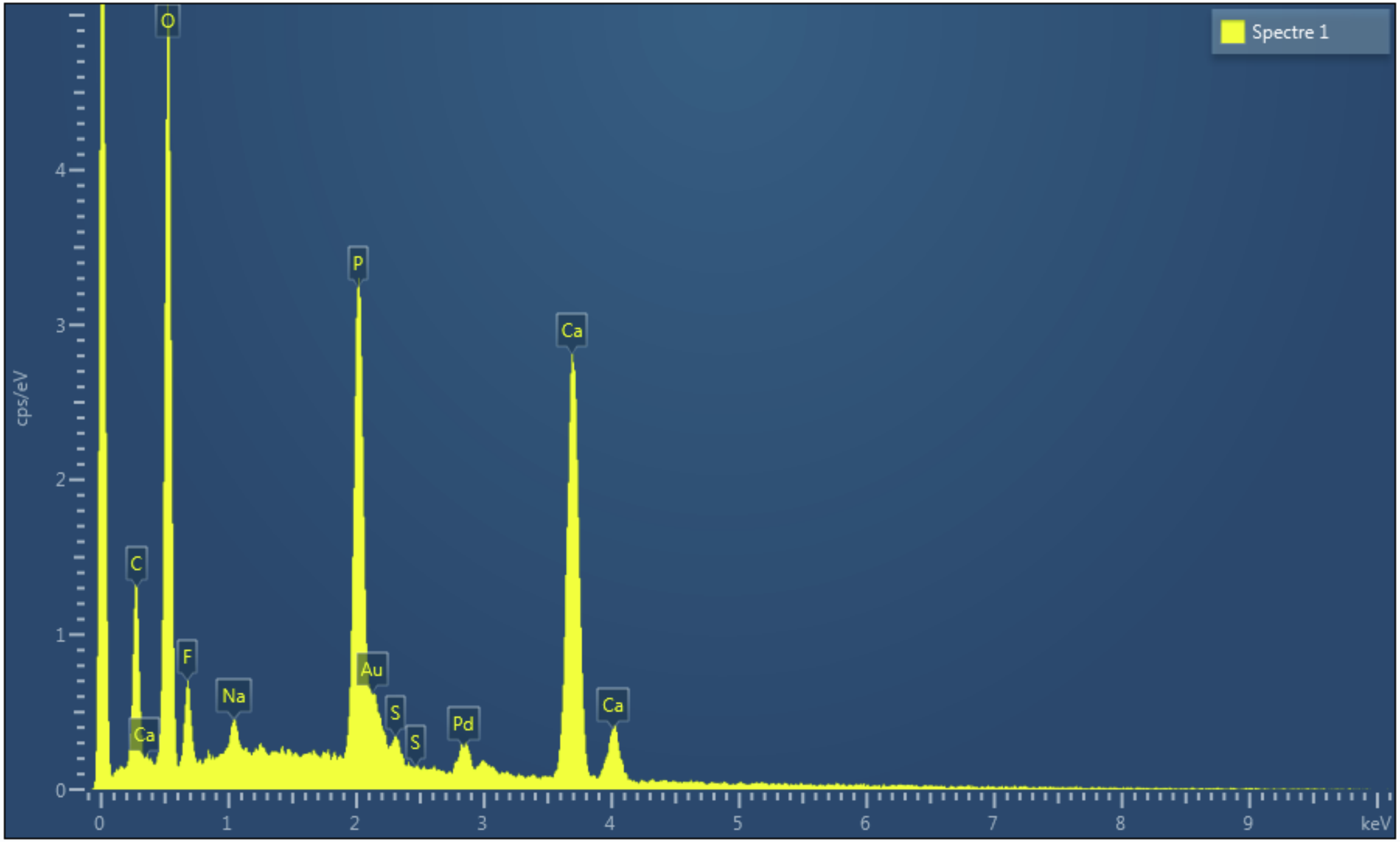

Figure S47: EDX Analysis of Sample LR1, Osteocyte, related to STAR Methods. Spectrum 1, of bone matrix.

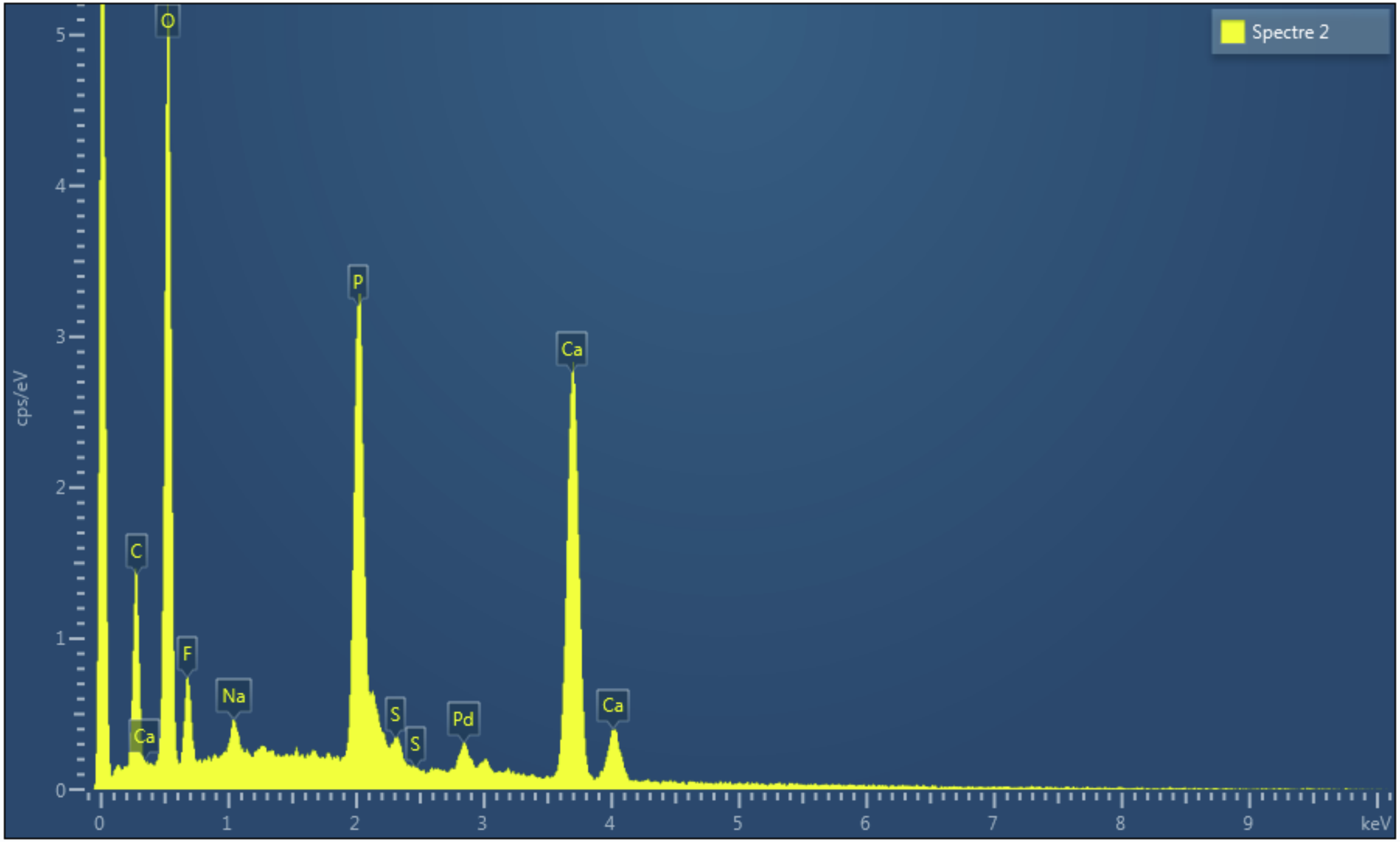

Figure S48: EDX Analysis of Sample LR1, Osteocyte, related to STAR Methods. Spectrum 2, of bone matrix.

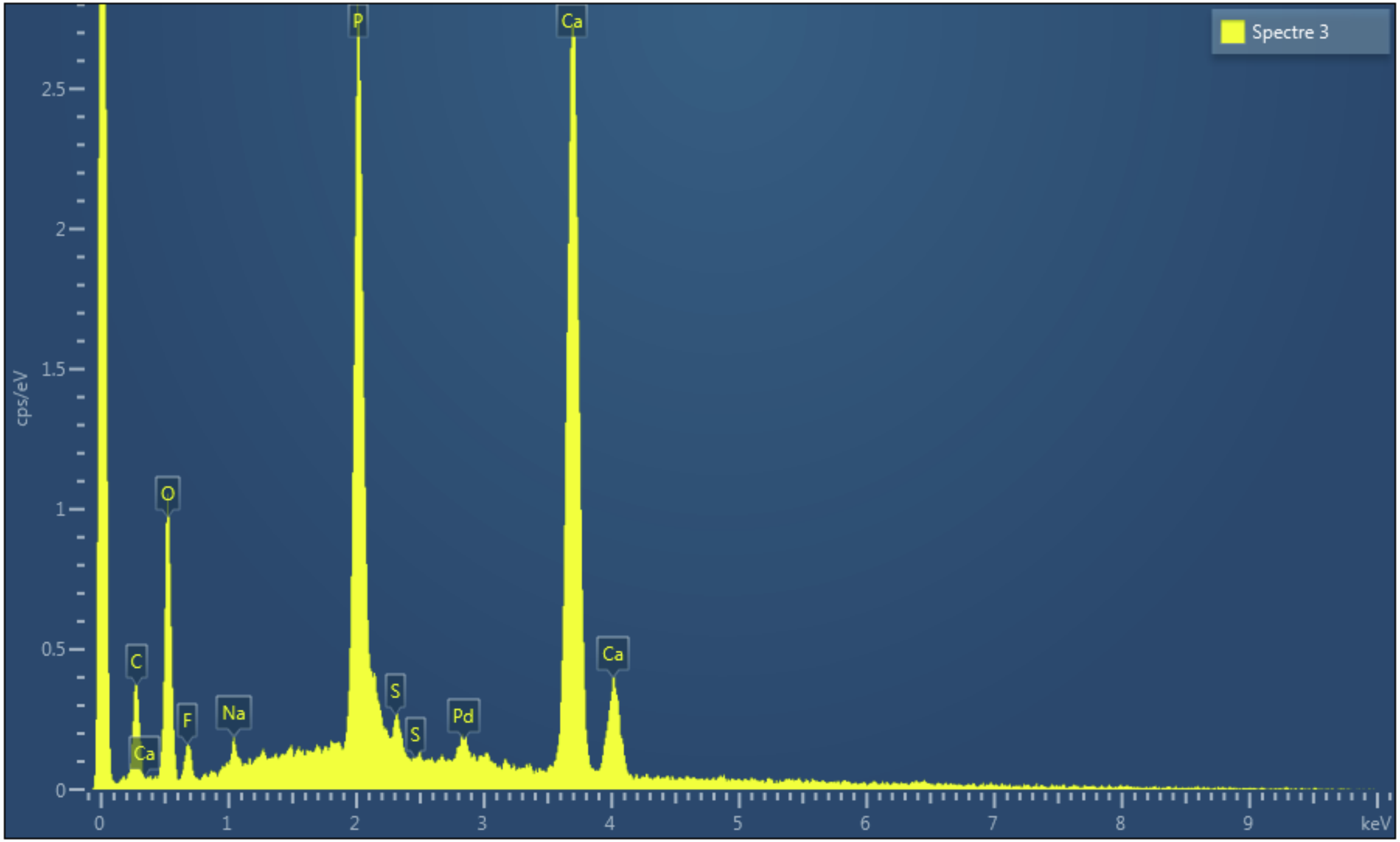

Figure S49: EDX Analysis of Sample LR1, Osteocyte, related to STAR Methods. Spectrum 3, of outside of cast shell.

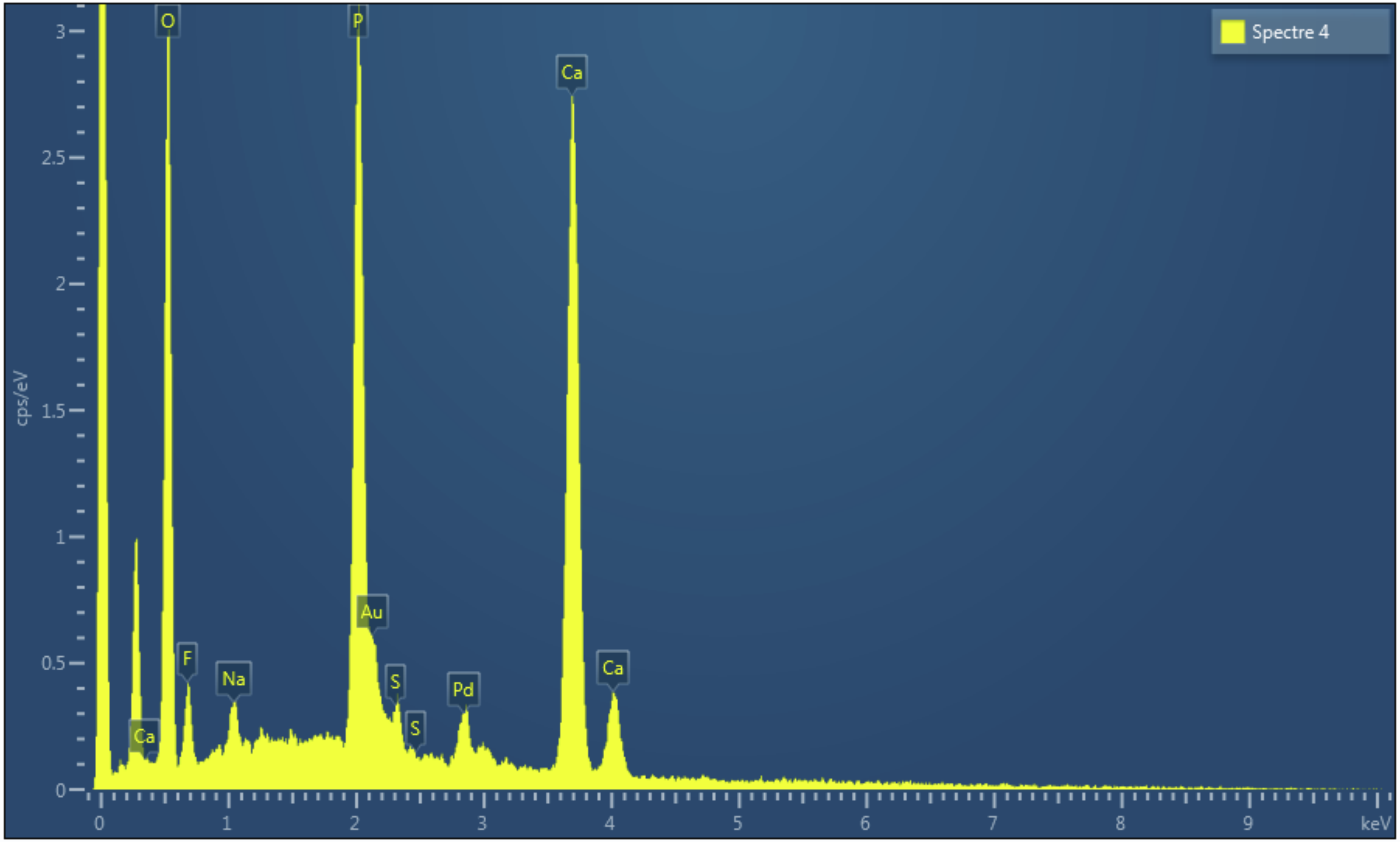

Figure S50: EDX Analysis of Sample LR1, Osteocyte, related to STAR Methods. Spectrum 4, of outside of cast shell.

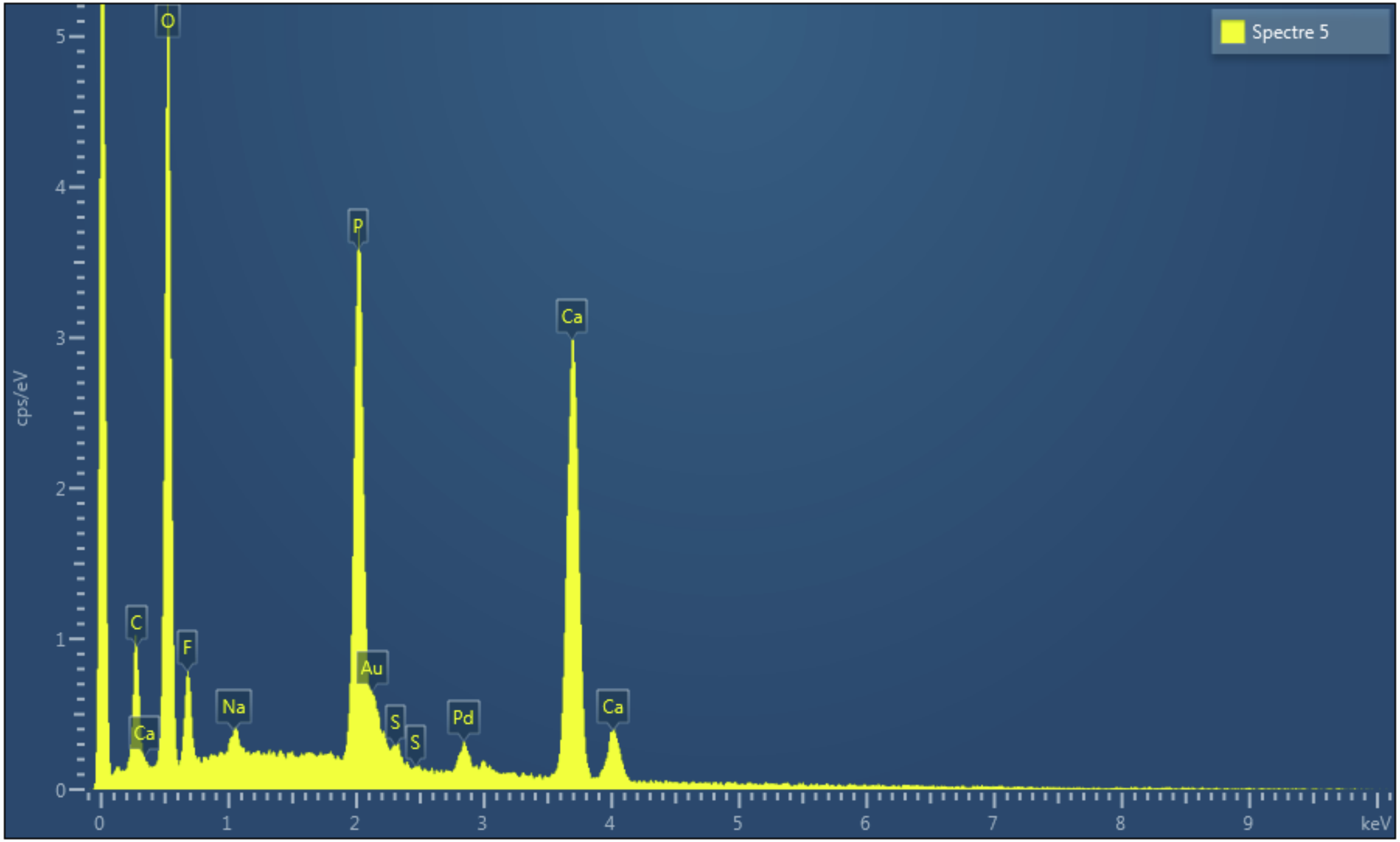

Figure S51: EDX Analysis of Sample LR1, Osteocyte, related to STAR Methods. Spectrum 5, of intside of cast shell.

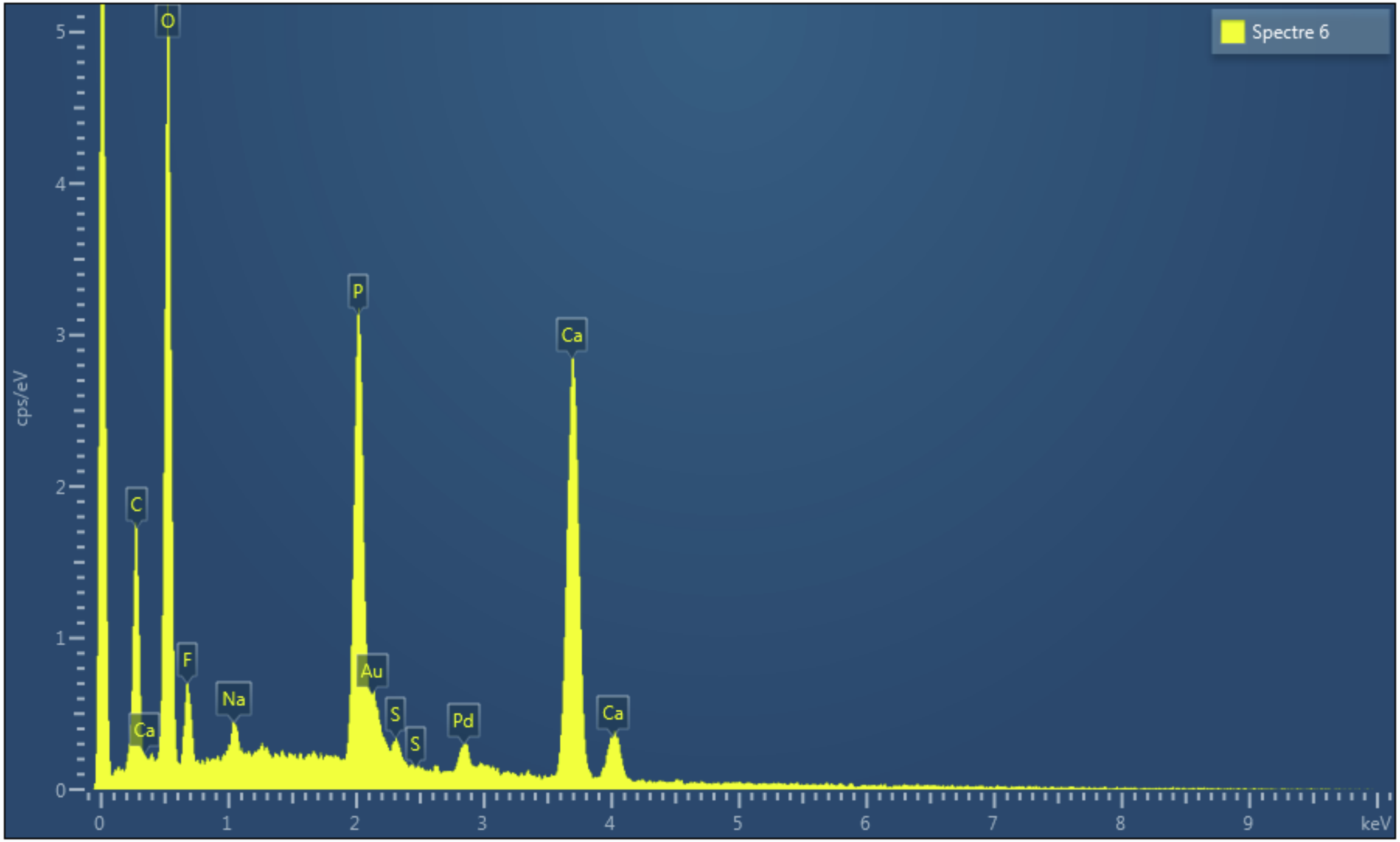

Figure S52: EDX Analysis of Sample LR1, Osteocyte, related to STAR Methods. Spectrum 6, of large crystal.

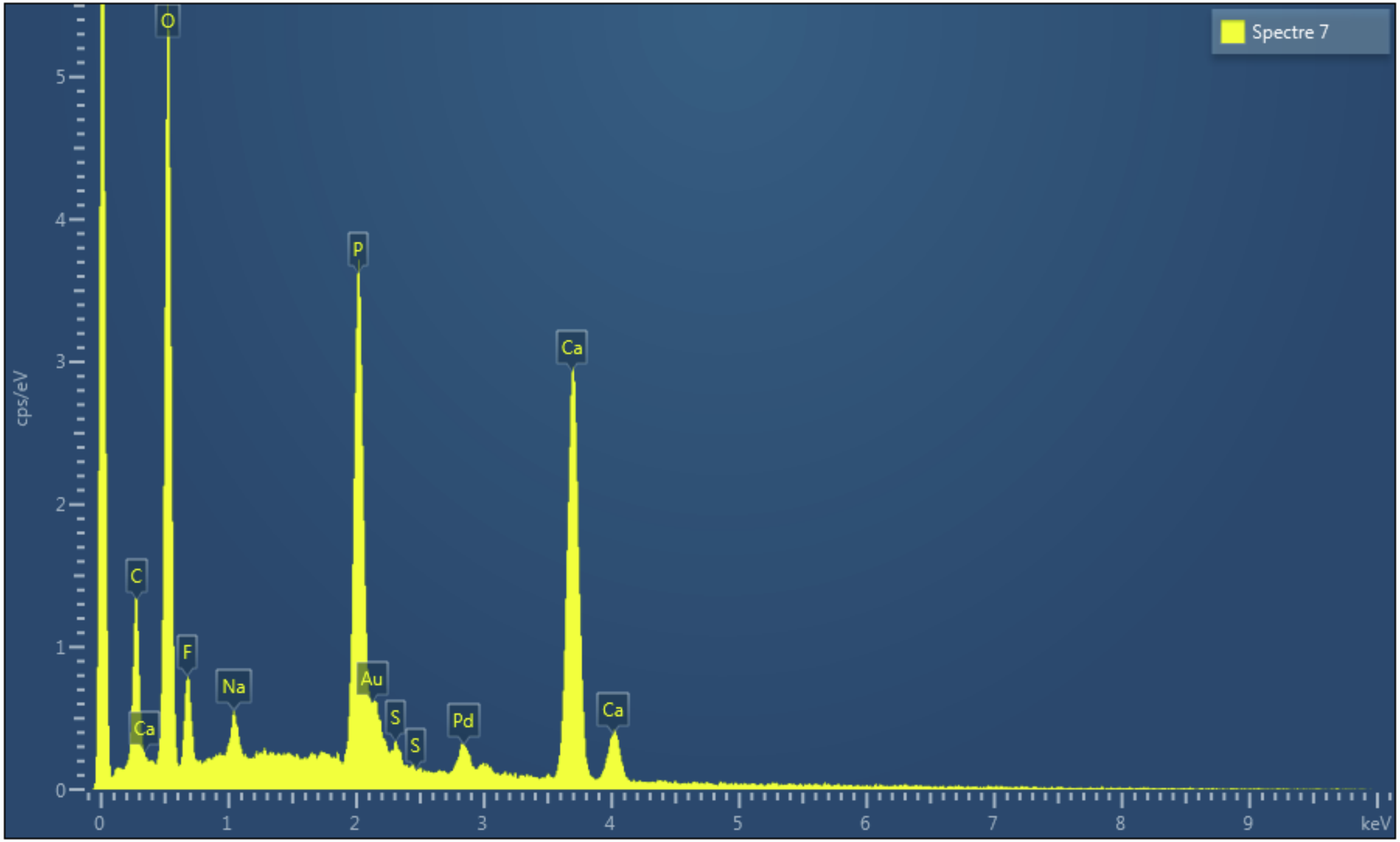

Figure S53: EDX Analysis of Sample LR1, Osteocyte, related to STAR Methods. Spectrum 7, of large crystal.

Table S1: EDX analyses of sample LR1, bone matrix and cartilage, related to STAR Methods. Atom percentages for the different spectra. Gold and paladium in the spectra are not included in the statistics.

Element/ spectrum	6	7	8	9
C	10.43	11.21	8.03	8.23
O	34.42	33.43	33.98	34.35
F	3.03	3.7	3.51	3.63
Na	0.72	0.74	0.76	0.7
P	14.66	14.32	14.87	14.68
S	1.26	1.19	1.14	1.3
Ca	35.47	35.41	37.71	37.11
Sum	100	100	100	100

Table S2: EDX analyses of sample LR1, bone matrix and cartilage, related to STAR Methods. Weight percentages for the different spectra. Gold and palladium in the spectra are not included in the statistics.

Element/ Spectrum	6	7	8	9
C	17.57	18.91	13.82	13.89
O	47.49	45.90	48.33	48.65
F	3.58	4.35	4.27	4.41
Na	0.69	0.71	0.75	0.69
P	10.50	10.17	10.92	10.75
S	1.01	0.93	0.92	1.03
Ca	19.16	19.04	21.00	20.59
Sum	100.00	100.00	100.00	100.00

Table S3: EDX Analysis of Sample LR1, Osteocyte, related to STAR Methods. Atom percentages for the different spectra. Gold and paladium in the spectra are not included in the statistics.

Element/ Spectrum	1	2	3	4	5	6	7
C	14.97	16.05	10.57	15.77	10.65	18.55	14.07
O	47.46	47.15	27.28	40.92	49.05	45.84	47.59
F	4.86	5.11	2.45	3.67	5.60	4.67	5.44
Na	0.66	0.67	0.52	0.77	0.52	0.68	0.93
P	10.64	10.33	17.18	12.38	11.77	9.71	11.11
S	0.74	0.72	1.24	1.09	0.51	0.74	0.64
Ca	20.67	19.96	40.76	25.42	21.91	19.81	20.22
Sum	100.00	100.00	100.00	100.00	100.00	100.00	100.00

Table S4: EDX Analysis of Sample LR1, Osteocyte, related to STAR Methods. Weight percentages for the different spectra. Gold and palladium in the spectra are not included in the statistics.

Element/ spectrum	1	2	3	4	5	6	7
C	8.82	9.6	5.11	8.71	6.34	11.04	8.3
O	33.87	33.93	15.27	27.47	33.94	33.43	33.94
F	4.05	4.28	1.62	2.88	4.52	3.99	4.54
Na	0.68	0.7	0.42	0.75	0.52	0.72	0.96
P	14.3	14.12	18.37	15.65	15.6	13.27	14.96
S	0.62	0.7	1.04	0.98	0.42	0.61	0.49
Ca	37.66	36.68	58.17	43.57	38.68	36.94	36.83
Sum	100	100	100	100	100	100	100

SUPPLEMENTAL TEXT related to STAR Methods

The purpose of the following sections is to expand on our observations on the fossilized bones, considering aspects and details that were omitted from the main text for want of space. In particular, we discuss the implication of our work for understanding bone fossilization. This supplement follows the organization of the main text.

Forming surfaces

Forming surfaces are recognised by the morphology of the mineralizing fronts seen in parallel fibered and lamellar bone (Figures 2A, 2B, 3C, 3D, S1A and S5-S7). They are characterized by lozenge-shaped aggregates of mineral that vary in size from place to place. As in modern bone[17,44], different stages of mineralisation could be recognized from the local texture of the forming surfaces, ranging from smaller and less elongated lozenge-shape aggregates of minerals to nearly continuous bundles of fibrils. Note that a new term, "tessele", has been introduced for the lozenge-shaped aggregates in some recent literature, e.g., reference[66].

In our ichthyosaur sample, new bone tissue formation commenced on both, resting and resorption surfaces, and is worth considering further both at the histological and nanostructural levels. The location of the forming surfaces in the centra is not related to their location, i.e., near the center or near the outer surface of the centrum. The location is also not related to the territory they occur in, i.e., endochondral vs. periosteal, nor the origin of the bone tissue, i.e., primary vs. secondary. Notably, the proportion of forming surfaces vs. resting surfaces differs between the individual ichthyosaur specimens. Forming surfaces are very rare in the late juvenile OV1 but rather common in the skeletally mature OV2 (Figures 2B,3C, 3D, and S5-S7) and in AB1. Quantification of these and other types of surfaces are difficult in LR1 because large areas of trabecular surfaces are obscured by early diagenetic pyrite deposition on this specimen. Osteoblast secretory activity on trabecular surfaces of Jurassic ichthyosaurs must have varied with the season, possibly indicating that OV1 had died at a different season of the year than OV2.

Notably, forming surfaces of woven bone were not observed in this study. Woven bone does not form at a surface but starts locally between cells where there was nothing previously, i.e., there is no requirement for a scaffold. However, where there is a mass of forming woven bone, it ends somewhere - at its limit - which is some kind of surface[1,2]. Mineralisation in woven bone is initiated by matrix vesicles and is extremely rapid, on a timescale of hours as against days in lamellar bone[17]. In samples made anorganic, the most recently formed and mineralized woven bone is extremely tender and crumbly and difficult to conserve without very careful handling. We do not conceive that such conditions prevail in nature, during taphonomy and fossilization, so it is no surprise that we did not observe 'forming surfaces of woven bone' in the four ichthyosaur fossils studied by us.

Resting surfaces

Resting surfaces are the most common type of internal trabecular surfaces we observed. There are distinct differences in the numbers of osteocyte lacunae on the trabecular bone surfaces among the

samples as well, with OV1 having lower densities than the three other samples. This correlates with the low amount of forming surfaces in OV1, but quantification of these observations is needed.

Domains are areas of uniform bone fiber orientation in a forming or resting surface. In our fossils, as in modern bone, the packing density of fibers deposited in a domain can vary from one layer to another: it is not uncommon to see a sparse network of parallel fibers in a given orientation superimposed on a denser network of subjacent parallel fibers with a different orientation (Figures 3D, S12, and S13).

Ordered and disordered domains are well known in modern mammalian bone[17]. Ordered domains consisting of nearly parallel branching fiber bundles are the most common type of organization in adult bone and characterize lamellar or parallel-fibered tissue types (Figures 2F, S9, and S10A). Two-dimensional disordered domains can be observed in more specific circumstances at the internal surfaces of infilling secondary osteons (Haversian canals), especially when the gutter becomes narrow and in the back-wall of some open osteocyte lacunae[17]. A less regular organization of collagen was also observed in the trabecular osteoporotic bones of 75-year-old women[44]. According to[17], two-dimensional disorder could be linked to reduced movement of osteoblasts on the bone surface during fiber deposition. In contrast, ordered domains of parallel fibers would imply synchronous movement of elongated osteoblasts, all oriented in a given direction, and secreting collagen fibrils parallel to their major axis as they migrate or oscillate in this preferred direction on the bone surface.

In modern bone made anorganic, so-called prolonged resting surfaces are abundant[10,17]. These surfaces are characterized by the fact that mineralisation has extended beyond the limit of the collagen fibers. In this case, the secretory activity of the osteoblasts has been at rest for long enough to allow mineralisation of the ground substance surrounding and beyond the fibers. Although resting surfaces are abundantly documented in all four ichthyosaur specimens, revealing a wide array of bone fibril orientations, prolonged resting surfaces are not. We observed prolonged resting surfaces in the back of some osteocyte lacunae. Although post-morten abrasion could potentially have altered resting bone surfaces[67], this process is unlikely to have affected the surfaces we studied because those surfaces are deep inside the ichthyosaur centra.

Osteocyte lacunae

It has long been known from light microscopic observations that osteocyte lacunae preserve well in fossil bone. It recently has also been recognized that in some fossils, these lacunae may house the remains of osteocytes that can be liberated by digestion of the fossil bone[46,47]. In the case of the Jurassic ichthyosaurs studied by us, no organic remains are preserved inside the lacunae, which are either open (e.g., Figures 4D-F and S15-S17A) or, in LR1, filled with diagenetic minerals (Figures 4G, S17B, S18 and S19). The mineral nature of the infill is indicated by the fact that the fill consists of idiomorphic crystals forming minute geodes (Figures S18B and S19). These crystals are apatite as indicated by EDX (see Figure S44-S53, Tables S1-S4).

As with modern bone, mineralized collagen fibrils are visible inside the open lacunae. The fibrils inside frequently, but not necessarily, align with the lacuna long diameter and with fibrils of the surface in which the lacunae are embedded (Figures 4D and S15). Numerous submicron-sized holes inside

the empty osteocyte lacunae represent the openings of canaliculi, having housed the filopodia (canalicular processes) of the osteocyte. Similarly small holes outside the lacunae on the trabecular surface (Figures 3I and S13A) correspond to the canalicular processes (filopodia) of an active osteoblast layer. Interestingly, Kaye et al.[68] in their figure 10E had already observed broken open osteocyte lacunae in fossil bone with bone fibrils inside, very much like what we report here. However, they interpreted the bone fibrils as bacterial filaments and did not state the age and taxon of their sample.

Examples of osteocyte lacunae opened up by osteoclast activity were seen in ichthyosaur specimen OV1 (Figures 4E and S17A). This process is known from modern bone as well (Figure 1F), where the osteocytes then become mobile again (Figure 1G) after having been liberated by the osteoclasts.

Phosphoric acid etching of both trabecular and section surfaces in LR1 exposes osteocyte-shaped bodies that either represent natural casts of osteocyte lacunae or natural casts of osteocytes (Figures 4G, S17B, S18B and S19). These casts are more resistant to etching than the bone matrix. The mineral nature of the casts in LR1 is apparent from the observation with BSE which reveals the casts to be of the same density as the bone matrix. EDX analysis (see Figure S44-S53, Tables S1-S4) reveals them to have exactly the same composition as the surrounding bone matrix, i.e., apatite. The casts carry impressions of bone fibrils on the cast surface (Figure S18B) which may indicate that they are lacunar casts because fossil degraded osteocytes typically do not show these impressions[46,47], but see[69,70]. It is not clear what mineralogical differences between bone matrix and casts would explain the greater etching resistance of the latter. Pyrite as the fill can be excluded because of its much higher density than the bone matrix (this would be apparent in BSE images), so apatite with a different composition appears likely. Alternatively, the larger crystal size of the apatite in the casts compared to the bone matrix nanocrystals, as well as their orientation perpendicular to the cast surface, could lead to greater etching resistance.

Resorption surfaces

Resorption surfaces are distinctive in our fossil material and occur on all kinds of mineralized substrate, including bone in the periosteal and endochondral territory and on calcified cartilage. In modern bone, Boyde & Jones[17] described two morphologies of resorption lacunae. ‘Snail track’ resorption traces result when the osteoclast can ‘eat and walk’ at the same time and are associated with the gentle sculpting of local bone surfaces. At the other extreme, patches of deep pits result when the osteoclasts stay in the same area, burrowing into it. These patches are associated with strong surface drifts, for which perhaps the best examples are in growth drifts of whole cortices in dense bone or opposing trabecular surfaces in spongy bone, the resorbing sides of tooth sockets in ‘orthodontic’ tooth movement, and in the walls of dental crypt to accommodate an expanding tooth germ within.

Cartilage and endochondral bone

Cartilage and the endochondral bone originating from it are integral components of the vertebral centra in amniotes, occurring on the anterior and posterior articular surfaces and on the rib articular surfaces[1]. In ichthyosaurs, cartilage is also present dorsally at the neurocentral suture surfaces because of the lack of fusion of the neural arch to the centrum[57]. Contrary to a common misconception, cartilage is not transformed to bone. When calcified, it merely, but very importantly, acts as an extremely rapidly assembled scaffold on (and in) which to grow bone. Cartilage cells can expand within the matrix that they produce around themselves and thus inflate the tissue volume before their extracellular matrix calcifies[1,2,31]. Bone cells cannot.

In fossils, the material nature of cartilage is harder to understand than that of bone and bone fibrils because of the poor record of unmineralized cartilage in fossils[34,35]. However, fossil cartilage is worth studying from a cytological perspective because processes are preserved that have been hypothesized but are difficult to observe in modern samples. The inherent problem of studying unmineralized cells in mineralized tissues in modern histology may be circumvented by the peculiarities of fossilization. We observe that fossilization provides a record of such cells and "presents" them together with the *in-vivo* mineralized extracellular component (Figures 5G, S26A, and S27).

Our observations of fossil cartilage were made on both internal surface features and in etched sections. These observations are of interest regarding hypotheses of cell death and transformation in the formation of endochondral bone. As noted, the early juvenile sample LR1 (Figures 5B, 5D, 5E, 5G, S26, and S27) is especially informative because of its early growth stage, when cartilage production and endochondral ossification was rapid. As a result, this specimen preserves more cartilage by volume than the other specimens and more features of the transition of calcified cartilage to endochondral bone can be observed.

Particularly striking are the many examples of osteocyte casts within chondrocyte lacunae, indicating that the de-differentiation of chondrocytes and re-differentiation into osteoblasts was an important process. These osteoblasts then secreted bone matrix inside the chondrocyte lacunae (Figure S26B) and differentiated into osteocytes. We have documented the presence of such osteocytes abundantly (Figures S26 and S27) and found that they are preserved as natural casts including their filopodia of diagenetic apatite minerals with sometimes large crystals. As in the osteocyte casts in bone, the casts show the impression of bone fibers and fibrils on their surfaces (Figure S27B). We did not yet observe any natural casts of osteoblasts inside chondrocyte lacunae. Nevertheless, LV1 attests to the importance of the transformation of chondrocytes to osteoblasts in ichthyosaurs already 200 Ma and the general importance of the process in endochondral ossification.

Extrinsic (Sharpey) fibers: Incorporation and mineralisation

Sharpey fibers as a component of the periosteal bone tissue occur at well defined locations in the ichthyosaur centra, adjacent to the anterior and posterior articular surfaces, representing the insertion of the extrinsic fibers of the joint capsule. The fibers are distinctive in incident light optical microscopy (Figure 5H) and not only are embedded in the bone matrix but also protrude as mineralized "stumps" into intertrabecular space (Figures 5I and S28). Sharpey fibers protruding into marrow space have

also been observed in modern bone. Such projections form in areas of slow bone growth, whereas in areas of fast bone apposition, Sharpey fiber mineralisation lags behind, resulting in a depression on the internal bone surface at the location of the fiber[8].

The integration of the extrinsic Sharpey fibers with the bone matrix is revealed in a striking way in sample OV1, in which intrinsic fibers wrap around extrinsic Sharpey fibers (Figures 5J and S28B) when the density of extrinsic fibers is high. This arrangement probably reinforces the anchorage of the Sharpey fibers in the bone and is probably the consequence of the restraints of space.

Bone fibrils close-up: Implications for fossilization

An important aspect of our discovery of preserved internal mineralized bone surfaces in Jurassic ichthyosaurs are the implications for the fossilization of bone in general. Chemically, fossil bone generally consists almost entirely of fluorapatite[43]. This leads to two further questions: (I) How can the collagen in the fresh bone be replaced by apatite without compromising bone histological structure? (II) what is the source of the additional phosphate that is required to build the replacing apatite[71]?. Fluorine replacing the hydroxyl group does not appear to be a limiting factor because of the abundance of this element in ground and pore water[71,72,73].

Beyond the restricted sample evidence[4,6,7,68,74], the varied preservation and different stratigraphic horizons of the Jurassic ichthyosaur fossils suggest that the kind of preservation we describe here may be much more widespread than previously believed. For one, the difference in macroscopic and pore fill preservation in a single bone, i.e., OV1 (Figures 4A, 4B, 6A, and S3), suggests that localized differences in fluid flow may have been responsible, caused by the relatively impermeable clay sediment. But also the differences in pore fill and the differences in embedding rock and locality between all four samples suggest as much.

Although all four samples come from rocks with a similar depositional origin, i.e., poorly oxygenated mud on the seafloor in a shelf environment[60-63], they differ in the mineral fill of the pore space in which there would have been bone marrow in the living animal. Open porosity in OV1 (Figure 4A) contrasts with an evenly distributed calcite fill and very limited pyrite in the pore space in OV2 and AB1 and with a fill of two generations in LR1 (pyrite first and calcite later). It may mainly be the amount of early mineralisation covering up the internal bone surfaces that determines the visibility of the bone fibrils and other features. Pyrite cannot be removed by acid, leaving such specimens less accessible to the study of internal surfaces at high magnification. Phosphoric acid etching reduces fibril and fiber and Sharpey fiber dimensions so that they decrease compared to the diameter of the continuous fibers observed on the bone surface without etching (Figure S4B).

Nanometric apatite crystals in OV1 vary in size. Well-preserved internal bone surfaces of open porosity have crystals about half the size (average: 66 x 19 nm) of the largest crystals found on surfaces originating from millimeter-sized trabecular fragments caused by diagenetic crushing. Intermediate size crystals were also observed, particularly on areas partially or completely filled with calcite, the latter having been revealed by acetic acid dissolution of the calcite filling the pores. Although we do not understand the origin of these differences, the larger crystals, which are less

numerous on the fibrils, may have originated from the smaller ones by Ostwald ripening during diagenesis[75].

Obviously, the fibrils on the resting surfaces of the fossil ichthyosaur bone are preserved in their full morphology, and any dissolution and reprecipitation processes of bone apatite and removal of bone collagen must have taken place below the level of the bone fibril. However, our TEM observations do not inform on this question since the fossil nanocrystals are the same size as the modern ones, but appear to take up the space once occupied by collagen, suggesting that there are some newly precipitated nanocrystals (Figures 6H and 6I).

Making bone anorganic: A model for bone fossilization?

The astounding preservation of the internal bone surfaces in the Jurassich ichthyosaur fossils raises the question of the process of natural maceration during fossilization. Under suitable conditions, all soft tissue components of the body degrade and disappear during early taphonomic changes after death: clean skeletal tissue samples can be retrieved from animals buried in sand where there is a good rainfall in modern examples (AB personal observations). McFadyean[76] recommended macerating bones in a well-rotting heap of stable manure, where we expect to find the temperature optimum for microbial proteolytic enzymes. This raises the question as to what extent microbial degradation (vs. autolysis) was involved 150 Ma ago? Surprisingly, fungi do not seem to have played a role because evidence of more than superficial fungal and bacterial boring in fossils is extremely rare, as opposed to its ubiquity in modern burials[73,77,78].

We return to the striking resemblance of our Jurassic ichthyosaur fossils to modern bone made anorganic. At low to medium magnification (field-of-view width of 100 to 500 µm) (Figures 2-5), the fossil bone surfaces of the Jurassic ichthyosaurs look the same, only crisper, than modern mammal and bird bone made anorganic. In this treatment, any unmineralized collagen is removed and the fiber bundles are retained as their mineralized skeletons with the shape of the smallest fibrils retained. Unfortunately, the resemblance of the fossils and fresh samples in the SEM does not inform us if the crystalline structure of the original mineral skeleton remained intact or how it was modified during fossilization.

The similarity of the fossil and extant samples suggests that the fossilization process might act in a similar fashion to the process of making modern bone anorganic. We consider enzymatic digestion (maceration) of the unmineralized matrix (osteoid) during fossilization most likely. The other methods for making modern bone anorganic are unlikely to offer an analog to fossilization because they involve high temperatures (pyrolysis) or a strongly oxidative environment. Experimental work has shown that decomposing bone will quickly become anoxic internally[79]. We caution that none of the techniques applied to modern samples may offer a good analog to bone fossilization because slow processes such as collagen hydrolysis may play a role. Some bone may be fully mineralized in less than 20k years, as evidenced by permineralized Late Pleistocene specimens (e.g.,[80]). On the other hand, most archaeological bone tissue[73] and much Late Pleistocene bone is poorly preserved and ultimately headed for destruction.

Numerous studies have confirmed and refined the seminal SEM observations made by Boyde et al. in the late 1960s[10], which showed that in lamellar bone, the mineral fraction of the anorganic surfaces retains the fibrillar morphology of the collagen fibrils at the resting surfaces and takes the form of a collection of lozenge shaped aggregates at the mineralisation front of the forming surfaces below the osteoid. According to the work of Benezra Rosen et al.[37], once the bone has been rendered anorganic, the mineral skeleton of the fibrils can also exhibit a periodicity of around 70 nm, consistent with the initial quaternary structure of the collagen of mineralized fibrils (d-banding). While some authors[81] have questioned the efficacy of certain chemical treatments for making bone anorganic, which they claim do not fully extract the collagenous phase embedded within the mineralized fibrils, Chen et al.[13] have demonstrated otherwise. Immersing mature cancellous bone in a 2.6% NaOCl solution at 37°C for 14 days preserves its original structure (the fine trabecular architecture is unaltered and the microstructural features are preserved) while reducing the collagen content to below 0.01%. Some chemical treatments, including NaOCl, can also induce artefacts by dissolution and re-precipitation of apatite crystals from the mineral skeleton[81], leading for example to the appearance of new crystals on the surface, superimposed on the preserved mineral skeleton. This has led to the development of the gentlest possible treatments to render bone anorganic, at least superficially, without degrading its mineral skeleton[82].

Interestingly, the excellent preservation of fibrils in fossil specimens suggests that the mechanisms that degrade and extract collagen during fossilization are similar to today's gentlest treatments to make bone anorganic, i.e., those best able to expose the mineralising front within the mineralising fibrils[10,82].

The fact that the fibrillar morphology of mineralized collagen fibrils can be preserved in the anorganic surfaces of resting surfaces of modern bone may seem surprising. It is incompatible with the classical model of intra-fibrillar mineralisation mainly at gap zones illustrated in many textbooks. The precise arrangement of apatite crystals inside and on the surface of collagen fibrils is still poorly understood[23]. A recent model posits the presence of intra-fibrillar mineralisation in the gap zone, combined with extra-fibrillar mineralisation in the form of a mineral shell of bio-apatite nanocrystals around the collagen fibrils[22,23]. This model is more compatible with that of Landis[83], who suggest that most mineral in bone is intrafibrillar, with observations of the mineral skeleton of fibrils whose morphology is intact after being made anorganic. In addition, since the pioneering work of A. Boyde et al. the gradual transformation of the mineral phase from discontinuous lozenge-shaped clusters to a continuous fibril network as the mineralisation matures has been observed many times[84].

Novel hypothesis of bone fossilization

Our observations may even hint at a novel hypothesis of bone fossilization in general: hypermineralisation through loss of osteocyte regulatory function. This hypothesis accounts for the ubiquitous presence of well-preserved bone in the fossil record, independent of geological age, embedding rock, depositional environment, taphonomy, and taxonomy. This independence would suggest an immediate post-mortem process for fossilization that acts before all of these other factors can influence the fate of the bone. The hypothesis starts with the observation that osteocyte death is

delayed compared to that of the entire animal. When the osteocytes have died, too, one of their major functions, prevention of hypermineralisation of the bone tissue[85], will end. As a result, hydroxyapatite precipitation from supersaturated extracellular fluids still present in the early post-mortem bone matrix will proceed unchecked but not replacing the existing organic remains such as osteocytes and neurovascular bundles. The hypothesis thus assumes that these purely organic structures would only decay after the initial mineralisation is finished. Evidence consist of the frequent preservation of degraded osteocytes in fossil bone from deep time[45,46]. However, this hypothesis does not account for the eventual loss of the collagen from the bone fibrils. Experimental work[71] will be needed for clarifying these questions.

SUPPLEMENTAL REFERENCES related to Supplemental Text

66. Buss, D.J., Rechav, K., Reznikov, N., and McKee, M.D. (2023). Mineral tessellation in mouse enthesis fibrocartilage, Achilles tendon, and Hyp calcifying enthesopathy: A shared 3D mineralization pattern. Bone *174*, 116818.
67. Bromage, T.G. (1984). Interpretation of scanning electron microscopic images of abraded forming bone surfaces. American Journal of Physical Anthropology *64*, 161-178.
68. Kaye, T., Gaugler, G., and Sawlowicz, Z. (2008). Dinosaurian soft tissues interpreted as bacterial biofilms. PLoS ONE *3*, e2808.
69. Cadena, E. (2016). Microscopical and elemental FESEM and Phenom ProX-SEM-EDS analysis of osteocyte- and blood vessel-like microstructures obtained from fossil vertebrates of the Eocene Messel Pit, Germany. PeerJ *4*, e1618.
70. Ullmann, P.V., Pandya, S.H., and Nellermoe, R. (2019). Patterns of soft tissue and cellular preservation in relation to fossil bone tissue structure and overburden depth at the Standing Rock Hadrosaur Site, Maastrichtian Hell Creek Formation, South Dakota, USA. Cretaceous Research *99*, 1-13.
71. Kral, A.G., Lagos, M., Guagliardo, P., Tütken, T., and Geisler, T. (2022). Rapid alteration of cortical bone in fresh- and seawater solutions visualized and quantified from the millimeter down to the atomic scale. Chemical Geology *609*, 121060.
72. Trueman, C., N. (2004). Forensic geology of bone mineral: geochemical tracers for post-mortem movement of bone remains. Geological Society, London, Special Publications *232*, 249-256.
73. Hagelberg, E., Bell, L.S., Allen, T., Boyde, A., Jones, S.J., and Clegg, J.B. (1991). Analysis of ancient bone DNA: techniques and applications. Philosophical transactions of the Royal Society of London Series B, Biological sciences *333*, 399-407.
74. Elorza, J., Astibia, H., Murelaga, X., and Pereda-Suberbiola, X. (1999). Francolite as a diagenetic mineral in dinosaur and other Upper Cretaceous reptile bones (Laño, Iberian Peninsula): microstructural, petrological and geochemical features. Cretaceous Research *20*, 169-187.
75. Trueman, C., Behrensmeyer, A., Tuross, N., and Weiner, S. (2004). Mineralogical and compositional changes in bones exposed on soil surfaces in Amboseli National Park, Kenya: Diagenetic mechanisms and the role of sediment pore fluids. Journal of Archaeological Science *31*, 721-739.
76. McFadyean, J. (1884). The Anatomy of the Horse: a Dissection Guide (Edinburgh, London: W. & A.K. Johnston).
77. Hackett, C.J. (1981). Microscopical focal destruction (tunnels) in exhumed human bones. Medicine, Science and the Law *21*, 243-265.
78. Bell, L.S. (1995). Post Mortem Microstructural Change to the Skeleton. PhD Thesis (London: University College London).
79. Pfretzschner, H.-U. (2004). Fossilization of Haversian bone in aquatic environments. Comptes Rendus Palevol *3*, 605–616.
80. Sander, P.M., and Andrassy, P. (2006). Lines of arrested growth and long bone histology in Pleistocene large mammals from Germany: What do they tell us about dinosaur physiology? Palaeontographica A *277*, 143-159.
81. Su, F.Y., Pang, S., Ling, Y.T.T., Shyu, P., Novitskaya, E., Seo, K., Lambert, S., Zarate, K., Graeve, O.A., Jasiuk, I., *et al.* (2018). Deproteinization of cortical bone: Effects of different treatments. Calcified Tissue International *103*, 554-566.

82. Shah, F.A., Zanghellini, E., Matic, A., Thomsen, P., and Palmquist, A. (2016). The orientation of nanoscale apatite platelets in relation to osteoblastic–osteocyte lacunae on trabecular bone surface. Calcified Tissue International *98*, 193-205.
83. Landis, W.J., Song, M.J., Leith, A., McEwen, L., and McEwen, B.F. (1993). Mineral and organic matrix interaction in normally calcifying tendon visualized in three dimensions by high-voltage electron microscopic tomography and graphic image reconstruction. Journal of Structural Biology *110*, 39-54.
84. Shah, F.A., Ruscsák, K., and Palmquist, A. (2020). Transformation of bone mineral morphology: From discrete marquise-shaped motifs to a continuous interwoven mesh. Bone Reports *13*, 100283.
85. Milovanovic, P., and Busse, B. (2020). Phenomenon of osteocyte lacunar mineralization: indicator of former osteocyte death and a novel marker of impaired bone quality? Endocrine Connections 9, R70-R80.
